# Not All Stroop-Type Tasks Are Alike: Assessing the Impact of Stimulus Material, Task Design, and Cognitive Demand via Meta-analyses Across Neuroimaging Studies

**DOI:** 10.1007/s11065-024-09647-1

**Published:** 2024-09-12

**Authors:** Veronika I. Müller, Edna C. Cieslik, Linda Ficco, Sandra Tyralla, Amir Ali Sepehry, Taraneh Aziz-Safaie, Chunliang Feng, Simon B. Eickhoff, Robert Langner

**Affiliations:** 1 Institute of Neuroscience and Medicine, INM-7, Research Centre Jülich, Jülich, Germany; 2 Institute of Systems Neuroscience, Medical Faculty and University Hospital Düsseldorf, Heinrich Heine University, Düsseldorf, Germany; 3 Department of General Psychology and Cognitive Neuroscience, Friedrich Schiller University, Jena, Germany; 4 Department of Linguistics and Cultural Evolution, International Max Planck Research School for the Science of Human History, Jena, Germany; 5 Institute for Experimental Psychology, Heinrich Heine University, Düsseldorf, Germany; 6 Clinical Psychology Program, Adler University (Vancouver Campus), Vancouver, Canada; 7 Key Laboratory of Brain, Cognition and Education Sciences, South China Normal University, Guangzhou, China

**Keywords:** Stroop, Cognitive control, Interference resolution, Neuroimaging meta-analysis, Activation likelihood estimation

## Abstract

The Stroop effect is one of the most often studied examples of cognitive conflict processing. Over time, many variants of the classic Stroop task were used, including versions with different stimulus material, control conditions, presentation design, and combinations with additional cognitive demands. The neural and behavioral impact of this experimental variety, however, has never been systematically assessed. We used activation likelihood meta-analysis to summarize neuroimaging findings with Stroop-type tasks and to investigate whether involvement of the multiple-demand network (anterior insula, lateral frontal cortex, intraparietal sulcus, superior/inferior parietal lobules, midcingulate cortex, and pre-supplementary motor area) can be attributed to resolving some higher-order conflict that all of the tasks have in common, or if aspects that vary between task versions lead to specialization within this network. Across 133 neuroimaging experiments, incongruence processing in the color-word Stroop variant consistently recruited regions of the multiple-demand network, with modulation of spatial convergence by task variants. In addition, the neural patterns related to solving Stroop-like interference differed between versions of the task that use different stimulus material, with the only overlap between color-word, emotional picture-word, and other types of stimulus material in the posterior medial frontal cortex and right anterior insula. Follow-up analyses on behavior reported in these studies (in total 164 effect sizes) revealed only little impact of task variations on the mean effect size of reaction time. These results suggest qualitative processing differences among the family of Stroop variants, despite similar task difficulty levels, and should carefully be considered when planning or interpreting Stroop-type neuroimaging experiments.

**Supplementary Information:**

The online version contains supplementary material available at 10.1007/s11065-024-09647-1.

## Interference Processing and the Stroop Paradigm

In everyday life, we are confronted with the need to regulate our behavior and override impulses and response tendencies that interfere with our overarching goals. Such interference often arises from the preferential processing of goal-irrelevant information because of habits, expectancy, attentional orientation, or memory-driven biases such as priming. Keeping behavior flexible and aligned with overarching goals requires cognitive control, which resolves interference by re-biasing the processing focus toward goal-relevant information (Diamond, 2013).

One of the best-known experimental tasks to elicit cognitive interference is the Stroop task. Introduced in 1935 by John Ridley Stroop (Stroop, 1935), the task requires participants to name the ink color of printed words naming semantically incongruent colors (e.g., the word “red” printed in blue ink) and of colored squares or crosses, which are used as a semantically non-interfering (“neutral”) control condition. Behaviorally, the Stroop interference effect manifests in healthy individuals as a delay in reaction time when naming the ink color of incongruent color-words, as compared to neutral conditions. Neuroimaging studies have shown that this effect goes along with increased activation in a widely distributed brain network including bilateral anterior insula, regions of lateral frontal gyrus and junction, intraparietal sulcus, and superior and inferior parietal lobules as well as the anterior midcingulate cortex and pre-supplementary motor area (Huang et al., 2020; MacLeod & MacDonald, 2000). These regions are often summarized as multiple-demand network (Duncan, 2010) and are generally recruited in tasks probing executive functioning.

Since its introduction, the Stroop task and Stroop-like phenomena have gained strong popularity in cognitive psychology and neuroscience as well as in clinical neuropsychological contexts. Stroop-like tests are widely adopted to assess executive impairments (Braga et al., 2022) and frontal lobe (dys)functioning (Cipolotti et al., 2016; Demakis, 2004), monitor drug effects (Pilli et al., 2013), and induce mental fatigue (Sun et al., 2021). Accordingly, the Stroop task is part of different psychometric test batteries, such as the Delis-Kaplan (Delis et al., 2001) or CANS-MCI (Tornatore et al., 2005) test battery, and different other test libraries (for example, PsyToolkit’s, https://www.psytoolkit.org/experiment-library/#exps; or the Psychology Experiment Building Language Test Battery, https://pebl.sourceforge.net; Mueller & Piper, 2014). The Stroop test is especially widely used in the clinical context to assess cognitive impairments in conditions such as attention-deficit disorder (Lansbergen et al., 2007), cardiovascular disease (Dintica et al., 2022; Shao et al., 2020), dementia and mild cognitive impairment (Rabi et al., 2020; Spieler et al., 1996), hepatic encephalopathy (Luo et al., 2020), obsessive–compulsive disorder (Gruner & Pittenger, 2017), or traumatic brain injury (Dimoska-Di Marco et al., 2011), to only name a few. A PubMed database query (on 11th of June, 2023) using the search string “stroop AND (clinic* OR patient* OR patholog* OR disease* OR disorder* OR syndrom* OR prodrom*)” yielded 5770 hits, further highlighting the outstanding relevance of this task in clinical and neuropsychological research.

## Variations of the Task

Originally, the Stroop task was presented in separate blocks of trials per condition, in which performance was measured as the total amount of time needed to verbally name the colors of words in a list. Since Stroop introduced the task in 1935, different variants of the task have evolved (Lezak, 2012; Macleod, 1991), all aiming to capture how the processing of one stimulus dimension interferes with the processing of another one when two stimulus dimensions of a multidimensional stimulus overlap (Kornblum & Lee, 1995). In the behavioral literature, Stroop-like experiments differ in many aspects with potential impact on interference, including manipulations of the experimental design and type of stimulus presentations, semantic variations of the irrelevant dimension, manipulations of the probability of conditions, size of the stimulus and response set, response modality, and variations in the control condition but also in the stimulus material (Macleod, 1991). Additionally, studies have investigated the effects of the list-wise proportion of incongruent items (Lindsay & Jacoby, 1994), thus manipulating the amount of proactive and reactive control (Braver et al., 2008), as well as proportion effects on the item-specific level (item-specific proportion congruent effect; Jacoby et al., 2003). In neuroimaging, the most common variations pertain to the use of different control conditions, the way the different conditions are presented (presentation design), and the inclusion of additional cognitive demands as well as various types of stimulus material. Given that this study focuses on effects observed in neuroimaging experiments, only these variations that are common in neuroimaging will be further considered.

Experiments using Stroop-like tasks differ, for example, in the kind of *control condition* used for comparison with the incongruent target condition. While the original experiment employed color patches (and crosses for the investigation of practice effects), later studies used strings of different colored characters (like XXX, %%%, $$$, ***) or neutral (color-unrelated) words as control condition (for overviews, see Macleod, 1991, 2005). In addition, 35 years after Stroop’s initial experiment, a “facilitation” condition was introduced, in which target and irrelevant stimulus dimensions were made congruent to each other, like the word “red” printed in red (Dalrymple-Alford & Budayer, 1966). Subsequently, more and more experiments directly compared incongruent with congruent conditions. Especially in neuroimaging research, examining the Stroop effect via contrasting an incongruent with a congruent condition has become very popular. The main reason for this preference might be the fact that in both incongruent and congruent conditions, the same stimuli (e.g., words) are used, and only the congruency is manipulated (MacLeod, 2005), therefore controlling for possible stimulus effects.

Another factor of variation between Stroop tasks lies in the *presentation design*. While Stroop originally presented lists of stimuli per condition (i.e., blocked design), neuroimaging studies have typically used trial-by-trial stimulus presentations, where incongruent, neutral, and/or congruent conditions are either presented in blocks of trials (similar to the original version but with trial-wise, rather than list-wise stimulus presentations) or by mixing conditions.

Further, there are task variants that impose *additional cognitive demands*, by which an additional cognitive process is required for performing the task. For example, this applies (i) if the Stroop task is presented in the form of a match-to-sample task (i.e., requiring participants to match the color of a word to the meaning of another presented word or the color of a cue held in working memory) (e.g., Schulte et al., 2009; Zysset et al., 2001), (ii) if there is no fixed response mapping (i.e., response mappings for the different colors change from trial to trial), or (iii) if the Stroop task is combined with another task (e.g., a stop-signal task: Basten et al., 2011), and participants therefore have to constantly switch between task requirements.

On top of these variations of control conditions, design, and demand, also, the specific type of task or *stimulus material* greatly varies across studies, too. Besides color-word versions, studies on Stroop-type conflict processing also used auditory, pictorial, spatial, numerical, dimensional, shape, or emotional stimuli to induce interference (for review, see Macleod, 1991). The most common types of these other Stroop-like tasks used in neuroimaging experiments are the numerical, counting, spatial, and face-word Stroop task. In addition, there are affective versions that use the processing of emotional words to induce interference with color naming/counting (Gotlib & McCann, 1984; Whalen et al., 2006) or with the naming of the emotion expressed in another stimulus dimension (De Houwer & Hermans, 1994; Stenberg et al., 1998). While in the former version (naming the color in which an emotional word is printed), the emotional word meaning does not directly interfere with the response regarding the task (as the target dimension is color), in the latter version (naming the emotion expressed in a face on which an emotional word is printed), the word meaning directly conflicts with the task-relevant dimension (identification of the emotional expression). Therefore, the emotional color-word interference task, which is traditionally called the emotional Stroop task, actually taps into a different phenomenon (Algom et al., 2004) with, importantly, no overlap between the different stimulus dimensions. This task is hence not within the focus of interest of the present work.

To conclude, even though all the aforementioned task variations are designed to investigate the interference of one stimulus dimension with another, they are still quite different. This might be due to adaptions of the task to the neuroimaging environment (e.g., Bush et al., 1998) and to the fact that different studies ask specific experimental questions. However, these differences in experimental setup and material might have an influence on the Stroop effect that is measured, both on the neuronal and also on the behavioral level.

## Effects of Task Variations on Behavior and Its Neural Mechanisms

There is preliminary evidence for an impact of the aforementioned task features on behavior and brain activity related to interference processing in Stroop-type tasks, but only a few studies have directly compared different variations and results are often inconsistent.

### Control Condition

#### Behavioral Level

From some previous studies, there is evidence that the type of control condition is crucial for the size of the Stroop effect (Macleod, 1991; Parris et al., 2022). It has been suggested that the Stroop effect involves conflict at multiple levels and reflects a combination of response, semantic (subsumed as informational conflict), and task conflict (Augustinova et al., 2019). Depending on the specific type of incongruent, congruent, and neutral stimuli used, different amounts of conflict might be captured. In particular, congruent conditions do not induce informational conflict and usually lead to facilitation, i.e., shorter reaction times compared to neutral conditions. Therefore, the comparison of incongruent to congruent conditions is a confluence of facilitation benefits from congruent stimuli (that is not induced by neutral stimuli) and conflict costs from incongruent stimuli (Macleod, 1991). Thus, the nature of the Stroop effect (i.e., its constituent cognitive processes) differs depending on the type of control condition chosen. To complicate things, while congruent stimuli do not lead to informational conflict (conflict between the information provided by different stimulus dimensions), they do, however, induce task conflict (Littman et al., 2019; MacLeod & MacDonald, 2000), as the two task sets of word reading and color naming are also in congruent conditions concurrently activated (Parris et al., 2022). Therefore, it is assumed that the comparisons against congruent conditions mainly reflect informational conflict, while those against neutral ones also involve task conflict (Shichel & Tzelgov, 2018).

#### Neural Level

Regarding variations of the control condition, neuroimaging studies have not directly investigated differences from contrasting incongruent with congruent or neutral conditions, respectively. However, indirect evidence can be derived from studies that have shown that congruent compared to neutral conditions also activate some regions of the so-called multiple-demand system (Bench et al., 1993; Carter et al., 1995; Zysset et al., 2001), that is, regions involved in processing incongruent Stroop stimuli. This therefore indirectly implies that the choice of the control condition affects neuroimaging results.

### Presentation Design

#### Behavioral Level

Regarding task design, blocking or mixing conditions affects cognition and processing requirements. Reaction times are usually longer in mixed compared to blocked designs. These so-called mixing costs, however, can differ for different experimental conditions (Los, 1996). Specifically, for the Stroop task, studies have indicated that the way experimental conditions are varied (i.e., between individual trials or blocks of trials) leads to differences in interference and facilitation effects (Boucart et al., 1999; Salo et al., 2001). Salo et al. (2001), for example, found facilitation effects (of congruent trials) in blocked but not in mixed designs, as well as stronger interference effects in blocked compared to mixed designs. Hasshim and Parris (2018) also showed larger interference effects when blocking conditions and additionally demonstrated that presentation design primarily affects response conflict effects. However, in contrast to Salo et al. (2001) and Hasshim and Parris (2018), Floden et al. (2011) reported stronger interference effects for mixed than for blocked presentations.

#### Neural Level

Only a few studies provide information on the neural effects of mixing or blocking experimental conditions in the Stroop task. Leung et al. (2000) indirectly compared the results of an event-related Stroop study with the results of a different study using a blocked design and found an overlap of 26% of voxels with more bilateral effects in the mixed presentation, whereas the blocked one revealed a more left-sided involvement. Additionally, sequence and adaptation effects have been observed in mixed designs (Egner, 2011; Egner & Hirsch, 2005), which suggest smaller interference effects when conditions are blocked. Furthermore, Floden et al. (2011) found reduced activation of posterior medial frontal regions in a blocked compared to a mixed design. Finally, besides Stroop-specific effects, there are well-known general effects of the way experimental conditions are varied in neuroimaging: blocked designs usually have higher detection power (Birn et al., 2002; Clark, 2012), are easier to implement, and are easier for participants to perform; on the other hand, they are confounded by anticipation and adaptation (Clark, 2012).

### Additional Cognitive Demands

#### Behavioral Level

Furthermore, additional cognitive demands can increase the size of the Stroop interference effect. Penner et al. (2012), for example, reported larger reaction time differences between incongruent and congruent conditions in a matching task, where an additional forced-choice comparison was included (via an instruction to indicate if the color of a presented word was the same as the word meaning of another word), as compared to when there was no matching requirement.

#### Neural Level

Similar to variations of the presentation design, there is only sparse evidence on the change of neural patterns with increasing cognitive demand in Stroop-type tasks. However, for cognitive-control tasks in general, it has been suggested that there is specialization in multiple-demand (MD) regions when demand is rather low (e.g., recruitment of left-sided regions for verbal tasks), but as cognitive load increases, more and more regions of the MD network are recruited (e.g., also recruitment of right-sided regions for verbal tasks) in a rather non-specific fashion (Shashidhara et al., 2019).

### Stimulus Material

#### Behavioral Level

Behaviorally, some studies have shown that the Stroop interference effect differs depending on the stimulus material used. For example, smaller Stroop effects for spatial compared to color-word versions have been reported (Banich, 2019; Capizzi et al., 2017; Hilbert et al., 2014) as well as stronger effects for emotional than non-emotional face-word versions (Chechko et al., 2013). In contrast, Mitchell (2005) and Zoccatelli et al. (2010) did not find differences in the Stroop interference effect between color-word, counting, and shape-word versions as well as between color-word and spatial versions.

#### Neural Level

Mitchell (2005) used different stimulus materials in the fMRI scanner and reported stronger Stroop effects in the left dorsolateral prefrontal cortex (dlPFC) for a color-word compared to a counting Stroop task. Zoccatelli et al. (2010) found overlaps between spatial and color-word Stroop versions in the anterior cingulate cortex (ACC), supplementary motor area (SMA), left inferior parietal lobe (IPL), right middle frontal gyrus, and cerebellum but in general stronger effects with larger activations for the color-word version. Banich et al. (2000) also compared spatial and color-word Stroop versions and found similar regions, but the location of activation varied between the different tasks. In line with behavioral findings, stronger Stroop effects in emotional compared to non-emotional task versions (Chechko et al., 2012, 2013) have been reported in inferior frontal gyrus, insula, SMA, cingulate cortex, and IPL, as well as visual and temporal areas.

## Co-recruitment, Relative Specialization, and the Multiple-Demand Network

As mentioned above, solving Stroop-like conflict has been associated with the activation of a distributed fronto-parietal network (Cieslik et al., 2015; Huang et al., 2020) in numerous studies. This network is not only engaged during Stroop-like tasks but in general during a broad set of different tasks and is therefore commonly called multiple-demand network (MDN, Duncan, 2010, 2013). It has been suggested that this network creates mental control programs by combining the required components for the task (Duncan, 2013; Shashidhara et al., 2019). This is, on the one hand, reflected by co-recruitment of regions of the MDN, especially in conditions of high cognitive demand with an increase in MDN activation in more difficult compared to easy tasks (Duncan, 2010; Fedorenko et al., 2013; Shashidhara et al., 2019). Shashidhara et al. (2019), for example, demonstrated progressive recruitment of the entire MDN when adding complexity, time pressure, and reward to a simple spatial maze task, reflecting an increased need of integration in more demanding and motivating tasks. Additionally, relative specialization within the MDN has on the other hand also been demonstrated (Assem et al., 2022; Shashidhara et al., 2019), especially when demand is low, potentially reflecting specific aspects of the tasks (instruction, rule, stimuli). Previous meta-analyses comparing different tasks (Stroop, spatial interference, Go/No-Go, Stop-Signal, etc.) of cognitive action control (Cieslik et al., 2015) support this notion, but there is little information on relative specialization within one specific task.

## Aim of the Current Study

In summary, Stroop-type interference phenomena are characterized by conflict that arises from two different stimulus dimensions (and their associated behavioral consequences) that show semantic overlap, leading to crosstalk and enhanced processing costs. A plethora of studies have used this paradigm to investigate interference processing, and previous neuroimaging meta-analyses have identified regions of the MD network to be involved when processing Stroop-like tasks (Chen et al., 2018a; Cieslik et al., 2015; Huang et al., 2020; Song et al., 2017). However, beyond sharing the common feature of interference between two stimulus dimensions, there are still differences in some task features like control condition, presentation design, and stimulus material. How co-recruitment and relative specialization within the MD network are reflected during Stroop-like interference processing has not been investigated in detail and systematically. In particular, there is the question of whether involvement of the multiple-demand network can be attributed to the higher-order conflict that all of the task variations have in common, or if aspects that vary between task versions play a modulating role. Based on the fact that Stroop-like effects share the common property of an overlap between two dimensions of a multidimensional stimulus, one would expect only little variations in the brain regions recruited. Neuroimaging evidence is, however, inconclusive regarding such commonalities and also some differences were found. Thus, by using meta-analyses (activation likelihood estimation and robust variance estimation) across neuroimaging experiments, we aimed to investigate this issue systematically. Given that the task is frequently and routinely used in clinical and cognitive neuropsychology, it is crucial to elucidate which task version affects brain activation in which way, as differences in regional recruitment between versions of the task may, in turn, influence the sensitivity of the given version to detect a particular cognitive deficit.

Our first aim was to assess the impact of variations of Stroop-like tasks and experimental setup on the neural level (i.e., variation of spatial location of convergence). Second and as a follow-up analysis, we aimed to investigate the effects of the same factors on behavior reported in those studies (i.e., impact of variation on effect size). In particular, we asked the question if the variation of the following factors, which vary across neuroimaging settings, influences the size of the behavioral Stroop effect and the regions recruited during processing Stroop-like tasks: (1) stimulus material (e.g., color-words, emotional face-word combinations, or other stimuli, (2) type of control condition in the color-word Stroop task (i.e., contrasting the incongruent condition with neutral vs. congruent conditions), (3) presentation design (blocking or mixing of conditions) in the color-word Stroop task, and (4) imposing additional cognitive demand in the color-word Stroop task.

Given that all task variations share common properties of interference of two stimulus dimensions, we expected to find a consistent behavioral Stroop effect as well as core regions of the multiple-demand network recruited across all task variations. However, given previous findings, we additionally expected some differences. With respect to the type of control condition chosen, we expected larger behavioral Stroop effects and regions of the MD system to be more consistently involved when contrasting incongruent with congruent, relative to contrasts with neutral control conditions, due to the added effects of facilitation and task conflict in congruent Stroop trials. Assuming that the presentation design would alter processing requirements, we also expected differences between blocked and mixed designs with respect to the average size of the Stroop effect and the spatial convergence of incongruency-related brain activity across studies. However, we have no specific hypothesis about the direction of the differences, as results in the literature are inconsistent, mixing costs are asymmetric, and mental and methodological effects operate differently. For experiments that impose additional cognitive demand during Stroop-type interference processing, we expected larger behavioral Stroop effects on average as well as a broader and more consistent recruitment of the MD network.

No study protocol was used.

## Methods

### Sample

As the primary goal of the current work was to investigate the neural correlates of the Stroop interference effect across various task implementations, literature search and experiment selection criteria were initially focused on neuroimaging studies. In a second step, all eligible experiments included in the neuroimaging meta-analysis were checked for eligibility for the behavioral meta-analyses. As a result, we also included studies in the neuroimaging meta-analysis that turned out to be not eligible for the behavioral meta-analysis (i.e., inclusion of studies that did not report all the necessary behavioral data for calculating effect sizes of behavioral effects). Thus, our behavioral results can only be generalized to the typical settings in neuroimaging experiments.

We did not seek to obtain an ethics vote for this study as our analyses did not include any individual participant data but were solely based on previously published aggregated data.

### Literature Search and Inclusion and Exclusion Criteria

This study was part of a larger project on the evaluation of Activation Likelihood Estimation (ALE) meta-analysis. Here, we built on previous meta-analyses of supervisory control (Cieslik et al., 2015) as well as emotional interference processing (Chen et al., 2018a). To extend our sample beyond the Stroop and Stroop-like experiments included in those previous studies, an additional literature search was carried out as part of three subprojects (not reported here), including experiments published before 30.05.2023. Three (LF, ST, TA) researchers conducted the literature search (conducted between 2020 and 2021) of experiments and extracted the relevant data (coordinates, space, sample size, and task variation for neuroimaging meta-analyses; reaction times, standard deviation/error, correlation, and task variation for behavioral meta-analysis). Another author (VM) double-checked the included experiments, extracted data, and updated the database in fall 2023 (which was again double-checked by a co-author). Disagreement in data extraction and inclusion was resolved by discussion between the respective two researchers who did the search and double-checked as well as by obtaining additional advice from other co-authors. Published neuroimaging experiments of Stroop tasks using functional magnetic resonance imaging (fMRI) or positron emission tomography (PET) were identified by queries of three databases: PubMed (https://pubmed.ncbi.nlm.nih.gov/https://pubmed.ncbi.nlm.nih.gov/), Google Scholar (https://scholar.google.de), and Web of Knowledge (https://apps.webofknowledge.com). The databases Scopus, the Networked Digital Library of Theses and Dissertations (NDLDT), and OADT.org were queried to identify pertinent theses, dissertations, and conference papers/posters.

In addition to the database queries, 96 authors were contacted and asked to provide result coordinates or images of contrasts of interest (see supplementary table S1 for information on studies for which additional information was provided) and/or information for effect size calculations if pertinent results were not reported in the publication (e.g., we asked authors of clinical studies for results of the healthy sample when only effects across patients and healthy controls were reported). Neuroimaging results provided by authors and shared as full images or coordinate tables were coded as three peaks per cluster to keep the number of peak coordinates similar to those usually reported in the literature. We aimed at a sample size as large as possible with a minimum of 17 experiments needed for calculating neuroimaging meta-analysis (Eickhoff et al., 2016; Frahm et al., 2022).

Due to the lack of a system for risk assessment of individual studies included in neuroimaging meta-analyses, quality assessment was implemented via the detailed coding of methodological features of each study and strict quality-related inclusion criteria, thereby excluding experiments not meeting those standards or not providing the relevant information for assessment.

In particular, the following inclusion/exclusion criteria were applied:

- We selected studies that investigated Stroop-like phenomena in a non-clinical adult sample and reported (or provided us upon request) coordinates of the results of contrasting the incongruent task condition with a congruent or neutral control condition.
- Only experiments were included that used two-dimensional stimuli with a task-relevant dimension that featured overlap with the irrelevant stimulus dimension, therefore using stimuli with a logical relationship between dimensions (Algom et al., 2022). We selected only those experiments where the two stimulus dimensions activate different processes (for example, *reading* versus *color naming*, *reading* versus *counting*), while experiments where the two stimulus dimensions activate the same process (e.g., global–local interference tasks; both task-irrelevant and task-relevant dimensions activate *letter reading*) were excluded.
- Furthermore, to avoid strongly imbalanced heterogeneity on the level of presentation modality, only experiments using visual stimuli were considered, whereas experiments using other stimulus modalities or cross-modal settings were excluded.
- Experiments with a Simon component were excluded, i.e., where stimulus position interferes with the response.
- From the subset of emotional Stroop tasks, we excluded classic color-emotion-word and emotional counting Stroop tasks because they do not induce a semantic or response conflict (Feng et al., 2018) but induce a delay in response times due to attentional capture by the emotionality of the target word. Additionally, they don’t meet the criteria of a logical relation between stimulus dimensions.
- In addition, we only included experiments where a conflict was induced at both stimulus and response levels, as it is questionable if semantic conflict alone can be measured reliably (Hasshim & Parris, 2014, 2015). In turn, we excluded contrasts that only reflected one type of conflict, such as experiments where conflict only occurred at the level of the stimulus (perceptual/semantic conflict), which is, for example, the case when the response to a conflicting color-word is mapped onto the same response button (for example Chen et al., 2013) and is not part of the response set (for example Kim, 2010) or when semantically associated words are used instead of color-words (for example frog written in red ink, Banich et al., 2001). Furthermore, contrasts between response and semantic conflicts were also excluded (for example Chen et al., 2013).
- Due to the limited number of experiments fulfilling all other inclusion criteria, we excluded spatial versions of the task (*n* = 2) as well as studies posing additional cognitive demands for tasks other than the color-word Stroop (*n* = 3).
- Studies that re-analyzed data from the same participants as used in a different, already included experiment were excluded (for example, Videbech et al. (2003), who re-used the data from Ravnkilde et al. (2002)).
- We only included studies that reported results of whole-brain group analyses as coordinates in a standard reference space (Talairach/Tournoux (TAL) or Montreal National Institute (MNI)). Thus, results obtained from region-of-interest analyses (ROI) were not considered. Reported coordinates resulting from neuroimaging analyses using the software SPM (Statistical Parametric Mapping) or FSL (FMRIB Software Library) were treated as MNI coordinates, unless a transformation into Talairach space was explicitly mentioned. Differences in coordinate space (MNI vs. Talairach space) were accounted for by transforming coordinates reported in Talairach space into MNI coordinates using a linear transformation (Lancaster et al., 2007).
- Results from studies with patients or children were excluded as were experiments reporting between-group effects (for example, age- or disease-related effects) or pharmacological interventions. However, we did include clinical or intervention studies that reported within-group effects separately for the (healthy adult) control group or effects at baseline, respectively.

**Fig. 1:**
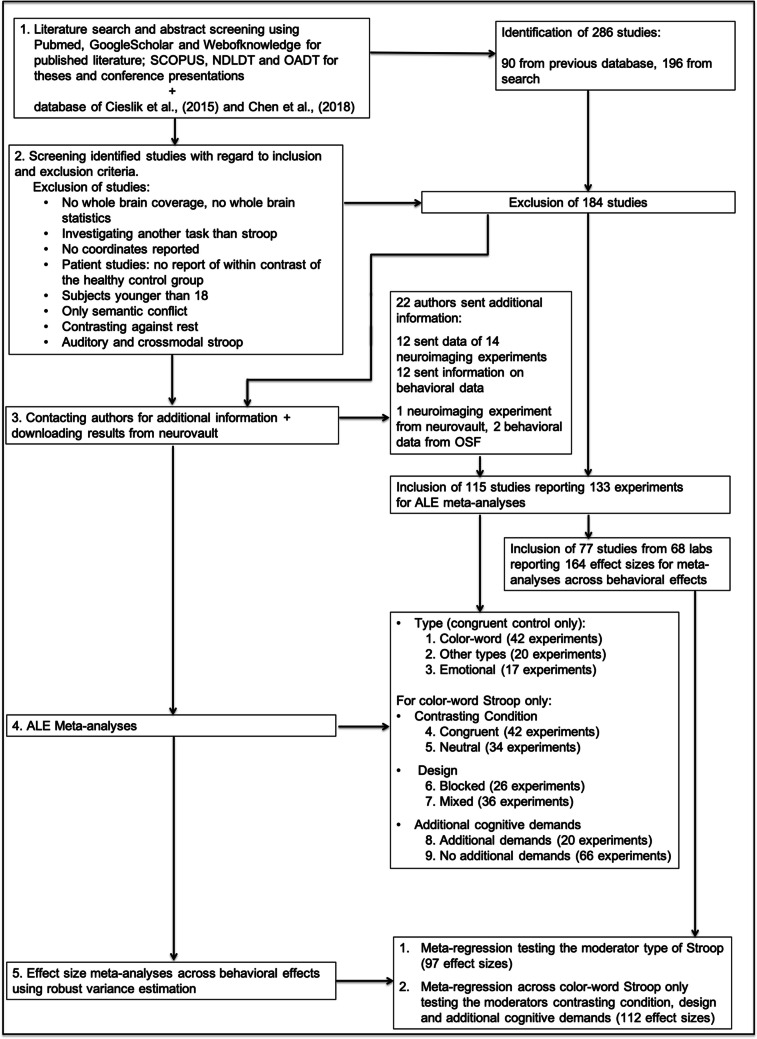
Illustration of the workflow of the meta-analytical study

These criteria led to the inclusion of 115 studies reporting 133 experiments (see supplementary table S3 for the checklist for neuroimaging meta-analyses and Fig. 1 for an illustration of the workflow of the current study). Of those studies, 77 studies from 68 independent labs reported (or provided) sufficient behavioral data used for calculating the effect size for the meta-analysis of the behavioral Stroop effect (see method description of the follow-up analysis for further details). Tables illustrating the data separately for the neuroimaging and effect size meta-analyses are presented in the supplement (table S1 and S2).

### Neuroimaging Meta-analyses

#### Coding of Experiments

##### Control Condition of Color-Word Stroop Tasks

Each color-word Stroop experiment was classified according to the condition to which the incongruent condition was compared (i.e., congruent or neutral). For either type of control condition, a separate meta-analysis was calculated: (1) congruent control (I > C, 42 experiments) and (2) neutral control (I > N, 34 experiments). Experiments that imposed additional cognitive demand were excluded from these analyses, as were experiments using different stimulus materials (i.e., other task types than the color-word Stroop version). Two supplementary meta-analyses were performed for which experiments reporting contrasts against a neutral control condition were separated into those using neutral words (18 experiments) and those using symbols/letters (17 experiments).

##### Presentation Design of Color-Word Stroop Tasks

Color-word Stroop experiments were further classified according to the way the experimental conditions were varied across trials as either blocked (trials of the same condition presented in blocks of trials) or mixed designs (random or pseudorandom mix of conditions across individual trials). We only classified experiments as a mixed design if also data analysis was done in an event-related manner. That is, experiments using a mixed presentation within blocks (i.e., congruent and incongruent blocks interspersed with neutral trials), which then compared different blocks with each other, were excluded from this analysis of design effects. For either type of design, a separate meta-analysis was calculated: (1) blocked (26 experiments) and (2) mixed designs (36 experiments). Again, experiments that imposed additional cognitive demand were excluded from these analyses, as were experiments using different stimulus materials. Studies reporting eligible results from the same participants in more than one experiment (e.g., studies reporting both incongruent > congruent and incongruent > neutral contrasts) were coded as one experiment to avoid biasing the meta-analysis by non-independent data (Müller et al., 2018a).

##### Additional Cognitive Demand in Color-Word Stroop Tasks

Another meta-analysis was calculated across color-word Stroop experiments that imposed some additional cognitive demand on top of the Stroop-type interference processing (20 experiments). These experiments included matching paradigms (for example, Zysset et al., 2001, introducing an additional forced-choice comparison where participants had to indicate if the color of a presented word is the same as the word meaning of another presented word) as well as combinations with different tasks (e.g., combined stop-signal and Stroop tasks). This meta-analysis was compared to an analysis across all color-word experiments without additional cognitive demands (across both kinds of control conditions and design, 66 experiments). Experiments using different stimulus materials were excluded, and studies reporting eligible results from the same participants in more than one experiment were treated as one experiment (cf. above).

##### Type of Stimulus Material

Finally, we performed a meta-analysis of other forms of Stroop tasks, that is, those that used other than color-word stimuli to induce Stroop-type interference. We categorized these experiments as follows: counting Stroop, numerical Stroop, face Stroop, spatial Stroop, or emotional face Stroop. We created three pools of experiments for separate meta-analyses, (1) emotional Stroop (17 experiments), (2) color-word Stroop (42 experiments), and (3) other versions (20 experiments), which were grouped together due to the limited number of experiments per stimulus material (counting, 8; numerical/size, 6; face, 6). Importantly, we only included experiments that used a congruent condition as control because none of the emotional face Stroop experiments and only very few of the other types of Stroop used a neutral control condition. Furthermore, we excluded experiments that imposed additional cognitive demand.

Please note that not all of the experiments could be classified with regard to all moderators of interest, and therefore, some experiments were included only in some but not all analyses (supplementary tables S1 and S2 show the coding of each experiment).

#### Activation Likelihood Estimation Algorithm

Standard analysis procedures were performed as used in previous ALE studies (cf. Caspers et al., 2010; Chase et al., 2015; Cieslik et al., 2015; Langner & Eickhoff, 2013). In brief, coordinate-based meta-analyses were performed to identify consistent activations across experiments by using the revised Activation Likelihood Estimation algorithm (Eickhoff et al., 2009, 2012; Turkeltaub et al., 2012) implemented as in-house Matrix Laboratory (MATLAB, 2019) tools. This algorithm aims to identify areas showing convergence of reported coordinates across experiments that are higher than expected for a random spatial association. Reported foci are not treated as single points but rather as centers for 3D Gaussian probability distributions capturing the spatial uncertainty associated with each focus. The width of these uncertainty functions is determined by the between-subject (uncertainty of spatial localizations between different subjects) and between-template (uncertainty of spatial localizations between different spatial normalization strategies) variance, which represents the main components of this uncertainty. Importantly, the between-subject variance is weighted by the number of participants per experiment, accommodating the notion that larger sample sizes should provide more precise approximations of the true activation effect and should therefore be modeled by smaller Gaussian distributions (Eickhoff et al., 2009). The probabilities of all foci reported in a given experiment were then aggregated for each voxel, resulting in a modeled activation (MA) map for that experiment (Turkeltaub et al., 2012). To ensure that results were not driven by studies reporting more than one eligible contrast obtained in the same group of participants (e.g., a study reporting both I > C and I > N contrasts), different contrasts included in one meta-analysis were coded as one experiment. Taking the union across the MA maps yielded voxel-wise ALE scores describing the convergence of results at each voxel of the brain. To distinguish true convergence across experiments from random overlap, ALE scores were compared to a null distribution (analytically derived, see Eickhoff et al., 2012) that reflects a random spatial association between experiments. Conceptually, the null distribution can be formulated as sampling a voxel at random from each of the MA maps and taking the union of these values in the same manner as done for the (spatially contingent) voxels in the true analysis. The *p*-value of an ALE score was then given by the proportion of equal or higher values obtained under the null distribution. The resulting non-parametric *p*-values for each meta-analysis were then thresholded at a cluster-level corrected threshold of *p* < 0.05 (cluster-forming threshold at voxel level, *p* < 0.001). Cluster-level family-wise error (FWE) correction was performed as suggested by Eickhoff et al. (2016) and Frahm et al. (2022) and described in detail in previous meta-analyses (Bzdok et al., 2012; Rottschy et al., 2012). First, the statistical image of the uncorrected voxel-wise *p*-values of the original analysis was thresholded at the cluster-forming threshold of *p* < 0.001. Then, the size of the clusters surviving this threshold was compared against a null distribution of cluster sizes. This null distribution of cluster sizes was derived by simulating 10,000 datasets of randomly distributed foci with identical properties (number of foci, uncertainty) as the original dataset. This distribution was then used to identify the cluster size that was only exceeded in 5% of all random simulations.

##### Individual Experiment Contributions

All clusters of significant convergence across experiments were further analyzed with regard to which experiments actually contributed to convergence. This was done by testing how much each included experiment contributed to the summarized ALE value. In particular, for each cluster and each experiment, the summarized ALE value of all voxels of the cluster with and without the experiment in question was calculated. If the summarized ALE value across all voxels of a cluster decreased when removing an experiment, that experiment was counted as contributing to the convergence of that cluster.

##### Contrasts and Conjunctions

To determine those voxels where a significant effect was present in two separate analyses, conjunctions were computed using the conservative minimum statistic (Nichols et al., 2005). That is, only regions significant on a corrected level in each individual ALE analysis were considered. To exclude smaller regions of presumably incidental overlap between the thresholded ALE maps of the individual analyses, an additional extent threshold of 25 voxels was applied (Langner & Eickhoff, 2013; Müller et al., 2018a, 2018b).

Differences between conditions were tested by computing contrast analyses as used in previous studies (cf. Cieslik et al., 2015; Rottschy et al., 2012). In particular, the difference between two ALEs was compared to a random distribution of differences under the null hypothesis of label exchangeability. First, the real difference between the two individual analyses was determined by computing the voxel-wise difference between the unthresholded ALE maps of each analysis (Eickhoff et al., 2011). Second, we determined a null distribution of differences. This was done by pooling all experiments contributing to either analysis and randomly dividing them into two groups of the same size as the two original sets of experiments. ALE scores for these two randomly assembled groups were calculated, and the difference between these ALE scores was recorded for each voxel in the brain. Repeating this process 25,000 times then yielded an expected distribution of ALE score differences under the assumption of label exchangeability. The observed difference in ALE scores was then tested against this null distribution yielding a posterior probability that the true difference was not due to random noise in an exchangeable set of labels, based on the proportion of lower differences in the random exchange. The resulting probability values were thresholded at *p* > 0.95 (95% chance for true difference) and inclusively masked by the respective main effects, i.e., the significant effects of the ALE analysis for the particular condition. In addition, an extent threshold of 25 voxels was applied.

### Follow-up Analysis: Meta-analyses Across Effect Sizes of the Behavioral Stroop Effect

Two authors screened all 115 studies included in the neuroimaging meta-analysis for eligibility for the behavioral meta-analysis. We only focused on the Stroop effect on response speed, as only some studies report data on accuracy. Studies were included if they reported sample size as well as mean reaction time (RT) and standard deviation or standard error of RT for the incongruent and congruent and/or neutral conditions. Numerical results presented in figures only were extracted with the web-based application of PlotDigitizer (Rohatki, 2021). Additionally, authors were contacted and asked to provide data and/or further information (see supplementary table S2 for information on which studies provided additional information). We included all available information in our analyses and thus allowed for multiple experiments (effect sizes) per study (see the “Robust Variance Estimation” section for detailed information on how we treated correlated effects). Disagreement on extracted data between authors was solved by discussion and with additional advice from co-authors. In total, 77 studies from 68 independent labs (studyIDs) reporting 164 experiments were included in the effect size meta-analyses of the behavioral Stroop effect.

The pool of data of the follow-up analysis features two different kinds of dependencies that need to be considered for the meta-analysis. First, the Stroop effect is generally obtained from within-subject designs, which requires considering the correlation between conditions (incongruent and congruent/neutral) for calculating the estimates for the effect size meta-analysis (Borenstein et al., 2009). Second, most studies reported multiple experiments per study leading to correlated effects within studies. To account for the former, we estimated the correlation between conditions (see detailed description below) by imputing a correlation coefficient from experiments where this information was available. The dependency structure resulting from including multiple experiments per study, in turn, was accounted for by robust variance estimation (see detailed description below).

#### Estimation of the Correlation Between the Incongruent and Congruent/Neutral Condition

For calculating effect sizes (ES) and standard errors (SE) for repeated measurements as obtained from within-subject designs typically employed in experiments on Stroop interference, not only mean RT and standard deviation (SD)/SE of the different conditions are necessary but also the correlation between RT scores of either condition. This correlation is, unfortunately, rarely reported. For experiments that provided a *t*-statistic between conditions, the standard deviation of differences, or figures with single-subject data, the Pearson correlation coefficient could be calculated from these values. In other cases, authors were contacted for information on the correlation coefficient or data from which the correlation could be derived. Of the 164 effect sizes included in total, we could obtain a correlation coefficient (or authors provided it on request) for 79 (of 35 studies) of them. From these 79 coefficients, we imputed a Pearson correlation coefficient by calculating an aggregated mean correlation coefficient using robust variance estimation (RVE) meta-analysis (see description of the method of RVE below), which was then applied for the remaining experiments where no correlation was available. The imputed correlation coefficient across all available correlations was 0.91. In order to test the impact of *r* on the estimates of the meta-analysis, a sensitivity analysis was performed. This was done by first calculating an intercept-only RVE random-effects meta-analysis across all experiments for which the real correlation was available and then repeating the same analysis but replacing the real correlation coefficient with a fixed one. In total, ten analyses were calculated (real *r*, *r* from 0 to 0.9 in steps of 0.1). The results of the sensitivity analysis revealed that estimates of the meta-analysis varied with changing correlation coefficients (table S4a). We therefore ran all behavioral meta-analyses three times: (1) using the observed correlation where available and the imputed correlation (0.91) for all experiments with a missing *r*, (2) using the observed correlation where available and a plausible minimum correlation coefficient for all other experiments (assuming a minimum coefficient of 0.6 given that for RT in similar tasks intercorrelations of *r* > 0.6 have been reported (see, e.g., Jensen & Reed, 1990; Larson et al., 1988), and (3) using the imputed correlation coefficient (0.91) for all experiments to keep this factor constant across experiments. Only the results of the first analysis approach will be presented, as there were no differences in results between the three approaches.

#### Calculation of Standardized Effect Sizes and Variance

For each experiment, standardized effect sizes and standard errors were calculated in R 4.1.1. (R Core Team, 2021) using the formula of Borenstein et al. (2009) for repeated measurements and correcting for small sample bias (Hedges g and SEg; Borenstein et al. (2009)). In detail, ES and SE were calculated based on RT means and SD/SE of the conditions as well as their corresponding Pearson correlation coefficient (*r*) for the 79 experiments where *r* was available; for all other experiments, ES and SE were based on the reported RT means and SD/SE as well as the imputed correlation coefficient of 0.91 (as described above). Effect sizes were calculated such that positive values reflect longer reaction times in the incongruent compared to the respective control condition (i.e., interference costs).

#### Robust Variance Estimation

As the inclusion of multiple effect sizes per study violates the assumption of independence of ES (Lipsey & Wilson, 2001), we adjusted our analysis for ES dependency by using robust variance estimation (RVE; Hedges et al., 2010; Tipton, 2013, 2015) using the robumeta package (version 2.0) in R (Fisher et al., 2017). Meta-analyses in general aggregate effect sizes by giving stronger weights to ES values with higher precision by inverse-variance weighting. In RVE, inverse-variance is also used, but additionally, the dependency structure between ES values is estimated and the weights adjusted accordingly (Hedges et al., 2010; Tanner-Smith & Tipton, 2016; Tipton, 2013). Based on the recommendation of Tanner-Smith and Tipton (2014), to determine the weighting scheme according to the most prevalent dependency structure, we used the correlated effect weighting scheme, as the highest amount of dependency resulted from having multiple effect sizes per study (only for ten studies, dependencies arose from hierarchical effects). For rho, we assumed a default of *ρ* = 0.8 as the within-study effect size correlation (Fisher & Tipton, 2015), necessary for RVE, as sensitivity analyses with varying *ρ* confirmed that estimates are not affected by *ρ* (table S4b).

#### Moderators of Interest and *Meta*-regression Models

An intercept-only random-effects RVE model across all effect sizes of the incongruence effect in reaction time was calculated for estimating an aggregated effect size across all experiments. As heterogeneity parameters (i.e., *I*^2^) estimated in RVE are not precise estimates of variance parameter estimates (see Tanner-Smith & Tipton, 2016), we did not test for heterogeneity. Then, two RVE mixed-effects meta-regression models were estimated, one testing different moderators for color-word Stroop tasks on the incongruence effect simultaneously and another one testing the single moderator “stimulus material.” For the first model, the moderators (i) control condition (congruent vs. neutral), (ii) presentation design (mixed vs. blocked presentation of conditions), and (iii) additional cognitive demand (yes vs. no) were included. In the second meta-regression model, only the moderator stimulus material (color-word/emotional/other) was included. Similar to what we did in the neuroimaging meta-analyses (see the “Coding of Experiments” section), we only included contrasts against congruent conditions in the second model as there were no contrasts against neutral control conditions for emotional Stroop and only very few for other types. Wald tests implemented in the clubSandwich package version 0.5.3 in R (Pustejovsky, 2021) were used as omnibus tests for categorical moderators with more than two categories.

Additional explorative analyses modeling the moderators type of neutral control condition, interaction between design and control condition, response modality, and a number of different colors can be found in the supplement (tables S5 and S6).

#### Analyses of Sample Bias, Outliers, and Robustness of Results

Sampling bias was examined by calculating an RVE meta-regression model with the standard error of Hedges *g* effect size (SEg) as a moderator (Rodgers & Pustejovsky, 2020), which is similar to Egger’s regression but adjusted for dependent effect sizes. The significance of the moderator was taken as an indicator of funnel plot asymmetry and therefore some sort of bias.

Additionally, influential ES values were identified as outliers by using case deletion diagnostics (Viechtbauer & Cheung, 2010) for both meta-regression models described above. Here, we fitted random-effects meta-analyses ignoring the dependency of effect sizes by using the influence function in the metafor package (3.4–0) in R (Viechtbauer, 2010).

### Transparency and Openness

We adhered to the Journal Article Reporting Standards for Quantitative Research in Psychology (Table 9 from Appelbaum et al., 2018) and the guidelines for neuroimaging meta-analyses (Müller et al., 2018a). We report how we determined our sample size, all data exclusion criteria, all manipulations, and all measures in the study. Analysis code of neuroimaging and behavioral meta-analyses can be found on the open science framework (OSF; https://osf.io/dt3kj/?view_only=995297bb53574583b1a0dda978f7f341); result files of the ALE meta-analysis are additionally available at ANIMA (https://anima.fz-juelich.de/; Reid et al., 2016). Behavioral data was analyzed using R, version R 4.1.1. (R Core Team, 2021); RStudio (RStudio Team, 2021); and the packages robumeta, version 2.0 (Fisher et al., 2017), metafor, version 3.4–0 (Viechtbauer, 2010), and clubSandwich, version 0.5.3 (Pustejovsky, 2021). Neuroimaging meta-analyses were analyzed with in-house Matlab, version 9.7.0.1471314 (MATLAB, 2019) tools. This meta-analytical project was not preregistered.

## Results

### Description of Included Experiments

We included 115 studies in total, reporting 133 experiments of neuroimaging results and 164 experiments of behavioral effects. The studies included a mean number of 29 participants with a mean age of 30 and on average an equal ratio of males and females. Eighty-five percent of the neuroimaging experiments required a manual response, and incongruent trials were presented with a probability of 42% on average. Seventy-two percent of the experiments used color-word stimuli. Of these color-word experiments, 57% reported contrasts against a congruent control condition, 41% presented conditions in blocks, and 21% included an additional component of demand. Experiments used on average 3.8 different colors as stimuli and 3.3 response alternatives (Table 1).

**Table 1:** Summary of included experiments describing the specific characteristics of experimental setups and participants

	All	Color-word only
% experiments using blocked design	34	41
% experiments using a congruent control	69	57
% experiment with no additional demand	85	79
% experiments color-word stimulus material	72	-
% experiments with manual response	85	80
Mean probability (SD) of incongruent trials	41.7 (10,6)	40.5 (11)
Mean (SD) number of participants	28.9 (26.8)	26.2 (21.5)
Mean (SD) age	30.1 (10.5)	29.8 (10.8)
Mean % (SD) of females	49.3 (23.1)	46.5 (24)
Mean (SD) number of response possibilities	3.1 (0.9)	3.3 (0.9)
Mean (SD) number of colors	-	3.8 (0.8)

### Neuroimaging Meta-analyses

Table 2, part B, provides an overview of all analyses and the results. For the meta-analyses across neuroimaging results, we first investigated the effects of control condition, presentation design, and additional cognitive demand across color-word Stroop experiments and then convergence across the different types of stimulus material used.

**Table 2:** Overview of all analyses and the main results

Analysis	Main results
*A. Meta-analyses of behavioral effects*	
1. Color-word Stroop only testing the moderators control condition, design, and additional cognitive demands	No significant effects for any moderator
2. Stroop and Stroop-like phenomena testing the moderator stimulus material	Significant effect of stimulus material: Larger effects for color-word Stroop compared to emotional Stroop Larger effects for color-word Stroop compared to other types of Stroop (when removing outliers)
*B. Neuroimaging meta-analyses*	
*Color-word Stroop:*	
Control condition	
1. Congruent control	Convergence in mostly bilateral multiple-demand system (bilateral anterior insula, lateral prefrontal cortex (PFC), left intraparietal sulcus (IPS), left dorsal premotor cortex, and posterior medial frontal cortex (pmFC))
2. Neutral control	Convergence in bilateral multiple-demand system (bilateral anterior insula, lateral PFC and IPS, pmFC)
Contrast analysis control condition	Slightly larger clusters for neutral compared to congruent control condition
Design	
3. Blocked	Convergence in left-sided and medial multiple-demand regions (anterior insula, lateral PFC, IPS, pmFC) and right orbitofrontal cortex
4. Mixed	Convergence in bilateral multiple-demand system (bilateral anterior insula, lateral PFC and IPS, pmFC, left dorsal premotor cortex (dPMC)) and left fusiform gyrus
Contrast analysis design	Stronger and more bilateral convergence in most multiple-demand regions as well as fusiform gyrus when conditions are mixed Stronger convergence in the right orbitofrontal cortex for blocked designs Differentiation within pmFC with stronger convergence for mixed presentation in pre-supplementary motor area (pre-SMA), more consistent activation for blocked designs in a more anterior cluster and convergence found for both analyses located between those two clusters
Additional cognitive demands	
5. Additional demand	Convergence in bilateral multiple-demand system (bilateral anterior insula, lateral PFC and IPS, pmFC)
6. No additional demand	Convergence in bilateral multiple-demand system (bilateral anterior insula, lateral PFC and IPS, pmFC)
*Stroop and Stroop-like phenomena:*	
Stimulus material	
7. Color-word Stroop	Convergence in mostly bilateral multiple-demand system (bilateral anterior insula, lateral PFC, left IPS, left dorsal premotor cortex, and pmFC)
8. Emotional Stroop	Convergence in regions of the multiple-demand system (bilateral lateral prefrontal cortex, left IPS, left ventral dorsal premotor cortex, right anterior insula, pmFC)
9. Other types	Convergence in pmFC, right anterior insula, and left anterior IPS
Conjunction across all stimulus material	Consistent recruitment of pmFC (pre-SMA and aMCC) and right anterior insula
Contrast analyses	Specific association of emotional Stroop with ventral dPMC (stronger convergence compared to color-word and other types) Specific association of color-word Stroop with lateral prefrontal cortex (stronger convergence compared to emotional and other types) other types of Stroop showed in general less convergence and only compared to color-word version stronger convergence in aIPS

#### Color-Word Stroop Task

##### Control Condition

**Fig. 2:**
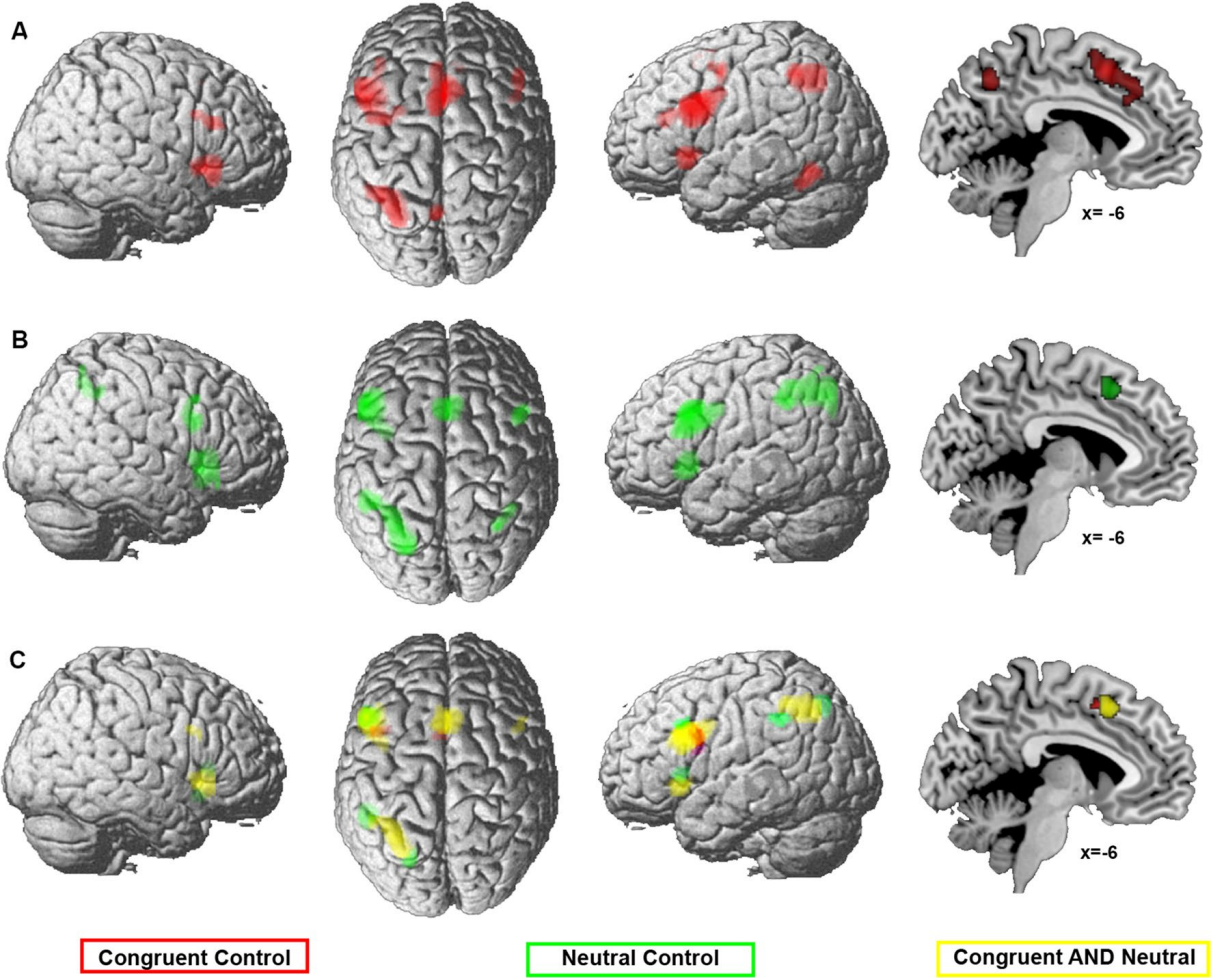
Meta-analysis across experiments using congruent or neutral conditions as control. This figure illustrates the results of the meta-analyses across color-word Stroop experiments comparing incongruent with **A** congruent (42 experiments) or **B** neutral control (34 experiments) conditions as well as the conjunction (**C** in yellow) and contrasts (**C**, red, stronger convergence for I > C; green, stronger convergence for I > N) between both analyses. The color intensity of the renderings (the first three columns) reflects the distance from the brain surface (lower intensity denoting further distance from the surface)

Two meta-analyses were calculated, one across all color-word experiments that contrasted against a congruent (I > C) and one across experiments with a neutral control condition (I > N). The two meta-analyses revealed similar regions of convergence (Fig. 2A and B, supplementary table S7 for main effects). Conjunction analysis, testing for regions that are significant in both meta-analyses, revealed convergence of both analyses in bilateral inferior/middle frontal gyrus/junction and anterior insula (aINS), left intraparietal sulcus (IPS), and posteromedial frontal cortex (pmFC; see Fig. 2C, shown in yellow for the conjunction).

To test where the convergence of results of the two meta-analyses significantly differed, a contrast analysis was calculated. This contrast between the two sets of results revealed stronger convergence for I > C in the left inferior frontal junction and the pre-supplementary motor area (pre-SMA) (Fig. 4C shown in red) and for I > N in the left inferior parietal lobe and posterior IPS, left middle frontal gyrus, and bilateral aINS (Fig. 2C, shown in green).

Results of supplementary analyses for which I > N experiments were separated depending on whether they used words or non-words (letters or symbols) as neutral control conditions are shown in supplementary Figure S1. Results are similar to the effects found for the combined analysis, with the only difference that convergence in the left aINS seems to be mainly driven by experiments using symbols and letters, while convergence in the right inferior/middle frontal gyrus/junction was only found in the analysis across experiments using neutral words.

In summary, the use of congruent but also neutral control conditions consistently involved regions of the multiple-demand system (bilateral aINS, dorsolateral prefrontal cortex, left IPS, and pmFC), with a slightly larger extent of most clusters when contrasting against a neutral control condition.

##### Blocked Versus Mixed Presentation of Experimental Conditions

**Fig. 3:**
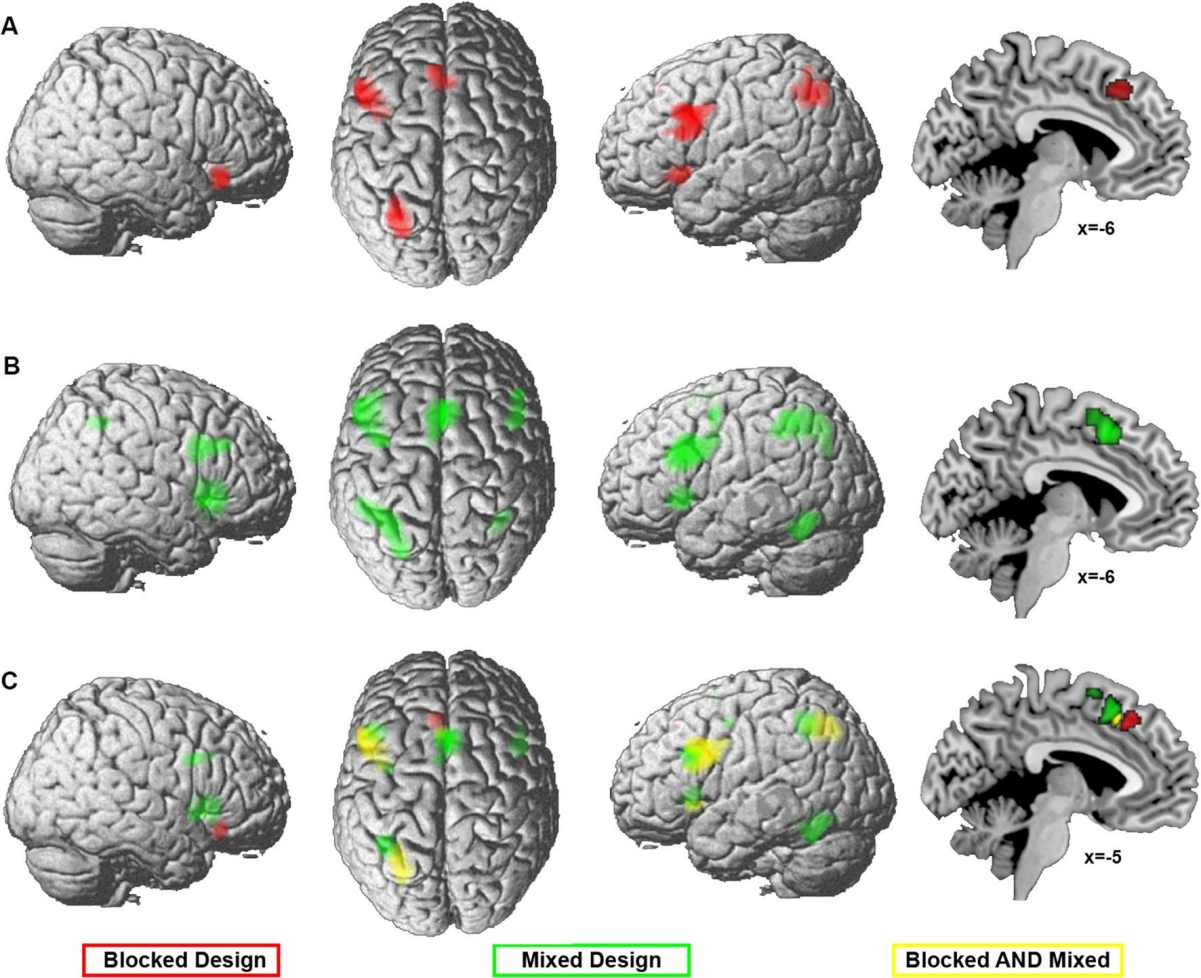
Meta-analyses across color-word Stroop experiments using **A** blocked (26 experiments) or **B** mixed modes (36 experiments) of presenting incongruent and congruent/neutral stimuli. **C** The results of the conjunction (yellow) and contrast (red/green: stronger convergence for blocked/mixed designs respectively)between the meta-analyses across experiments of either design. The color intensity of the renderings (the first three columns) reflects the distance from the brain surface (lower intensity denoting further distance from the surface)

Two meta-analyses were calculated: one across experiments that presented the different Stroop task conditions in blocks, and one across experiments implementing a mixed design. Figure 3A and B and supplementary table S8 present the main effects of blocked and mixed presentation designs, respectively. The conjunction between the meta-analyses for either type of presentation design revealed common convergence in the left inferior frontal gyrus (IFG), left IPS, and left aINS as well as pmFC (Fig. 3C, shown in yellow for the conjunction).

Contrast analyses that directly compared the results between both meta-analyses showed stronger convergence in more anterior pmFC and right orbitofrontal cortex for color-word Stroop experiments with a blocked design (Fig. 3C in red), whereas pre-SMA, bilateral aINS/frontal operculum, left middle intraparietal sulcus (mIPS), left fusiform gyrus, left precentral gyrus, and bilateral middle frontal gyrus were more consistently found for experiments using a mixed design (Fig. 3C in green).

In summary, our analyses of the impact of presentation design on the neural correlates of the Stroop effect point to a mainly left-sided convergence of interference-related brain activity in blocked designs and stronger and more bilateral convergence when task conditions are mixed. Additionally, a differentiation within the pmFC was found, with stronger convergence for mixed presentation designs in pre-SMA, more consistent activation for blocked designs in a more anterior cluster, and common convergence for both types of design located between these two clusters.

##### Additional Cognitive Demand

**Fig. 4:**
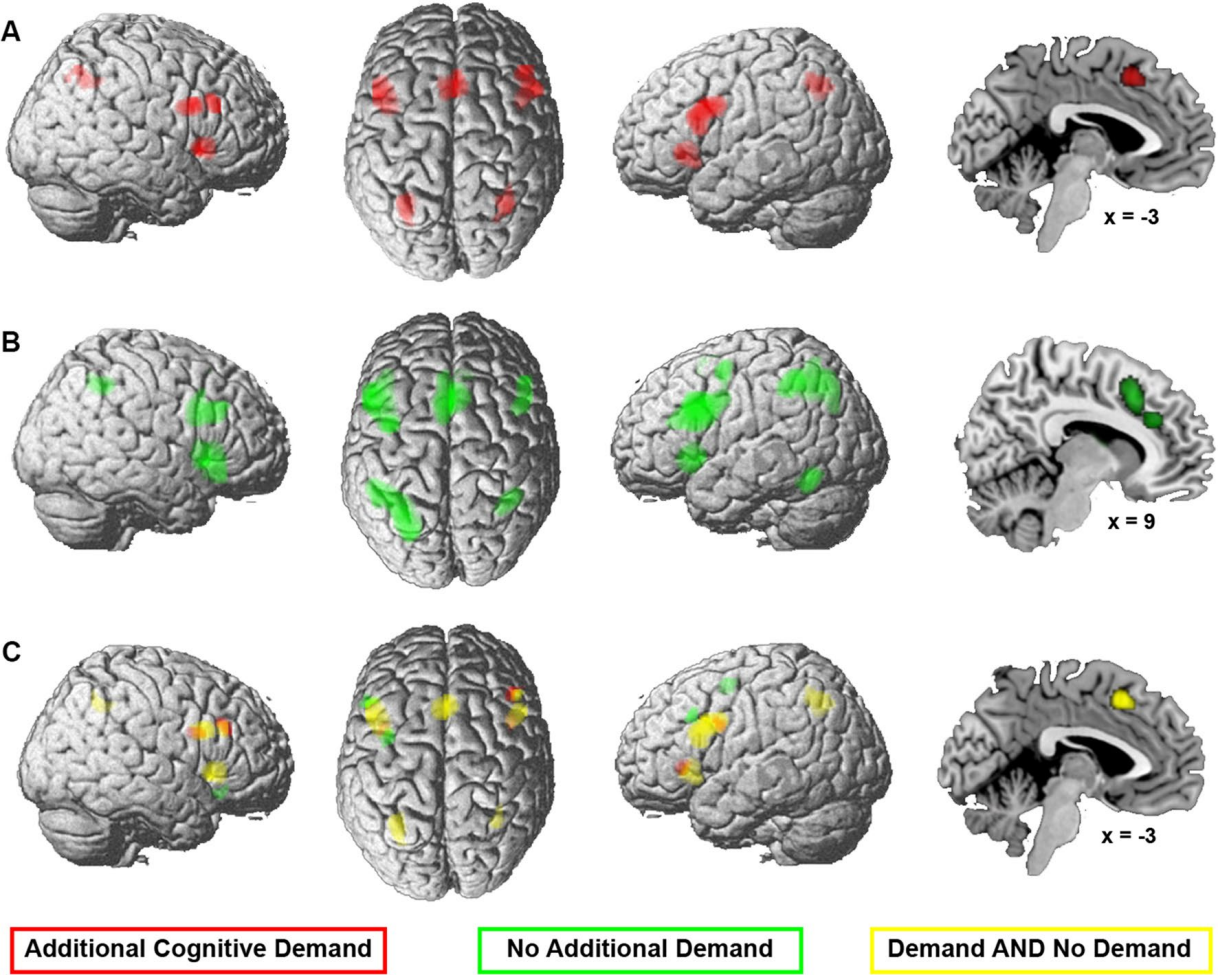
Meta-analyses across color-word Stroop experiments **A** with an additional cognitive demand component (20 experiments) and **B** without additional demand (66 experiments). **C** The conjunction (yellow) and contrast (red, additional cognitive demand; green, no additional cognitive demand) between both analyses. The color intensity of the renderings (the first three columns) reflects the distance from the brain surface (lower intensity denoting further distance from the surface)

Two meta-analyses were calculated to investigate the effect of additional cognitive demands on convergence; one analysis was calculated across experiments where an additional process was required for performing the task (matching, switching between task requirements, or response mapping) and one across experiments without an additional demand. Figure 4A and B and supplementary table S9 present the results of the main effects of the two meta-analyses of experiments with or without additional cognitive demand. The conjunction across both revealed common convergence for both analyses in bilateral aINS, bilateral middle/inferior frontal gyrus, bilateral IPS, and pmFC (Fig. 4C denoted in yellow).

Contrast analyses revealed stronger convergence for experiments with additional cognitive demand in left aINS, bilateral inferior frontal junction (IFJ), and right middle frontal gyrus (Fig. 4C in red) and for experiments without additional cognitive demand in the right orbitofrontal cortex, left middle frontal gyrus, and dorsal premotor cortex (dPMC, Fig. 4C denoted in green).

In summary, additional cognitive demand largely recruited the same network as without demand.

#### Different Types of Stimulus Material

**Fig. 5:**
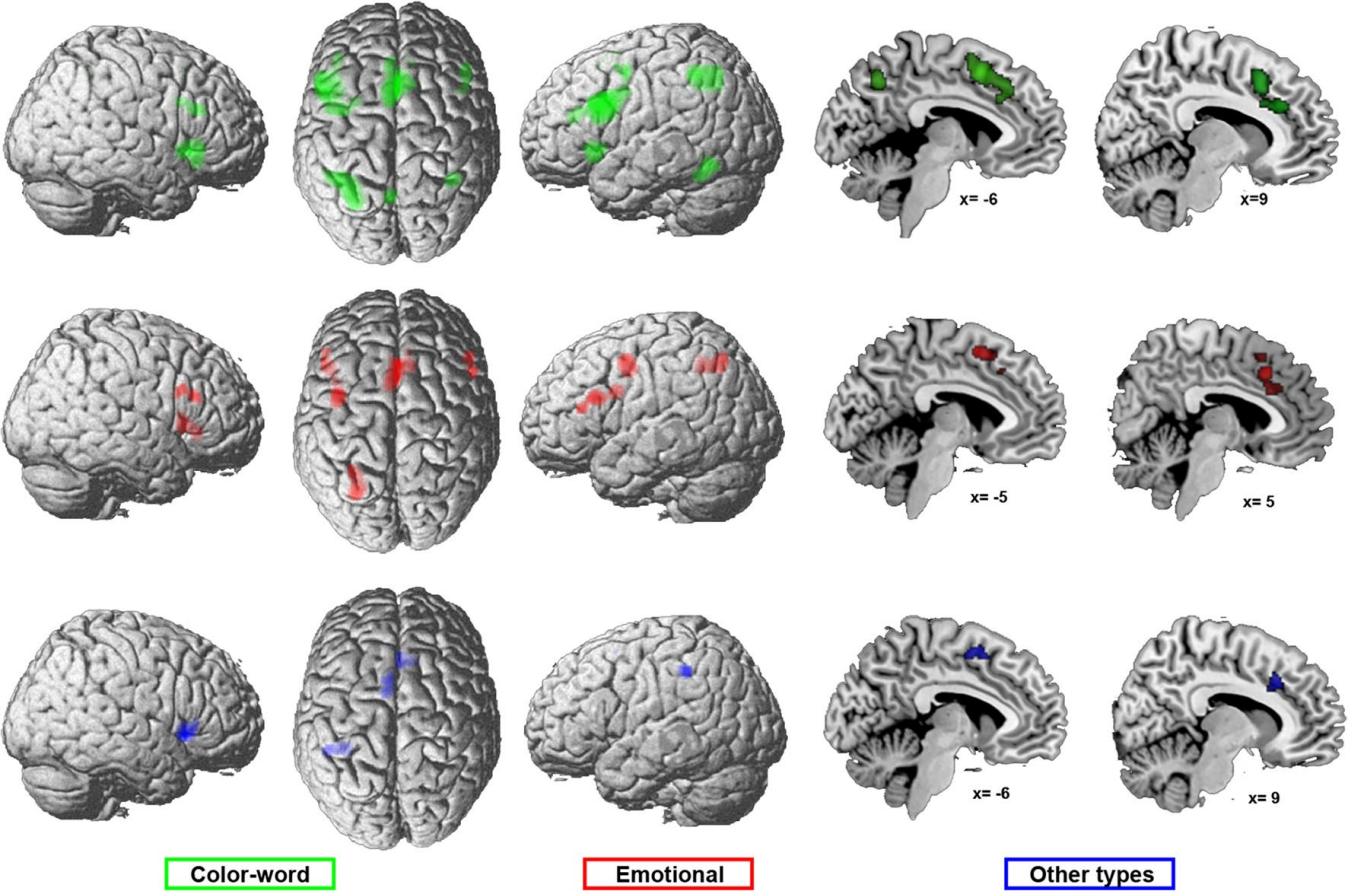
Main effects of the different types of stimulus material: meta-analyses across experiments using color-word Stroop (green, 42 experiments), emotional (red, 17 experiments), or other types of Stroop tasks (blue, 20 experiments). The color intensity of the renderings (the first three columns) reflects the distance from the brain surface (lower intensity denoting further distance from the surface)

We calculated three meta-analyses to investigate the influence of stimulus material: one meta-analysis across color-word Stroop experiments, one across emotional picture-word experiments, and one across other types of Stroop (including numerical, counting, and non-emotional picture-word Stroop varieties). The results of the three meta-analyses are shown in Fig. 5 and supplementary table S10. The convergences observed in the three meta-analyses were then compared to each other by a three-way conjunction as well as conjunctions and contrasts between all pairs of stimulus material.

**Fig. 6:**
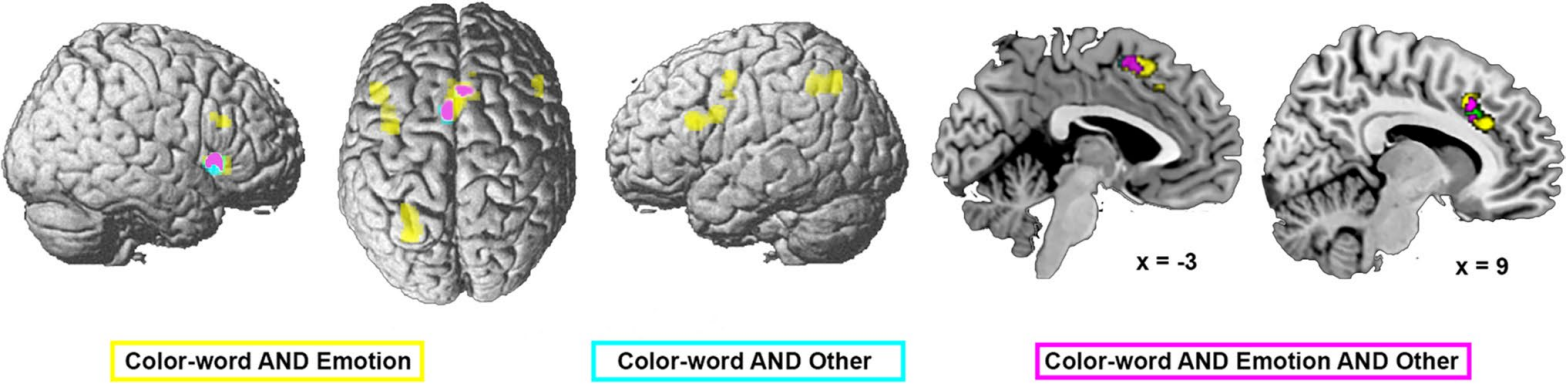
Conjunctions across the three meta-analyses of the different stimulus materials used, revealing overlap between all three types of material in the posterior medial frontal cortex (pre-SMA and aMCC) and right aINS (shown in pink). The color intensity of the renderings (the first three columns) reflects the distance from the brain surface (lower intensity denoting further distance from the surface)

All three meta-analyses (revealed via conjunction) showed common convergence only in pmFC (pre-SMA and anterior midcingulate cortex) and right aINS (Fig. 6 in pink).

**Fig. 7:**
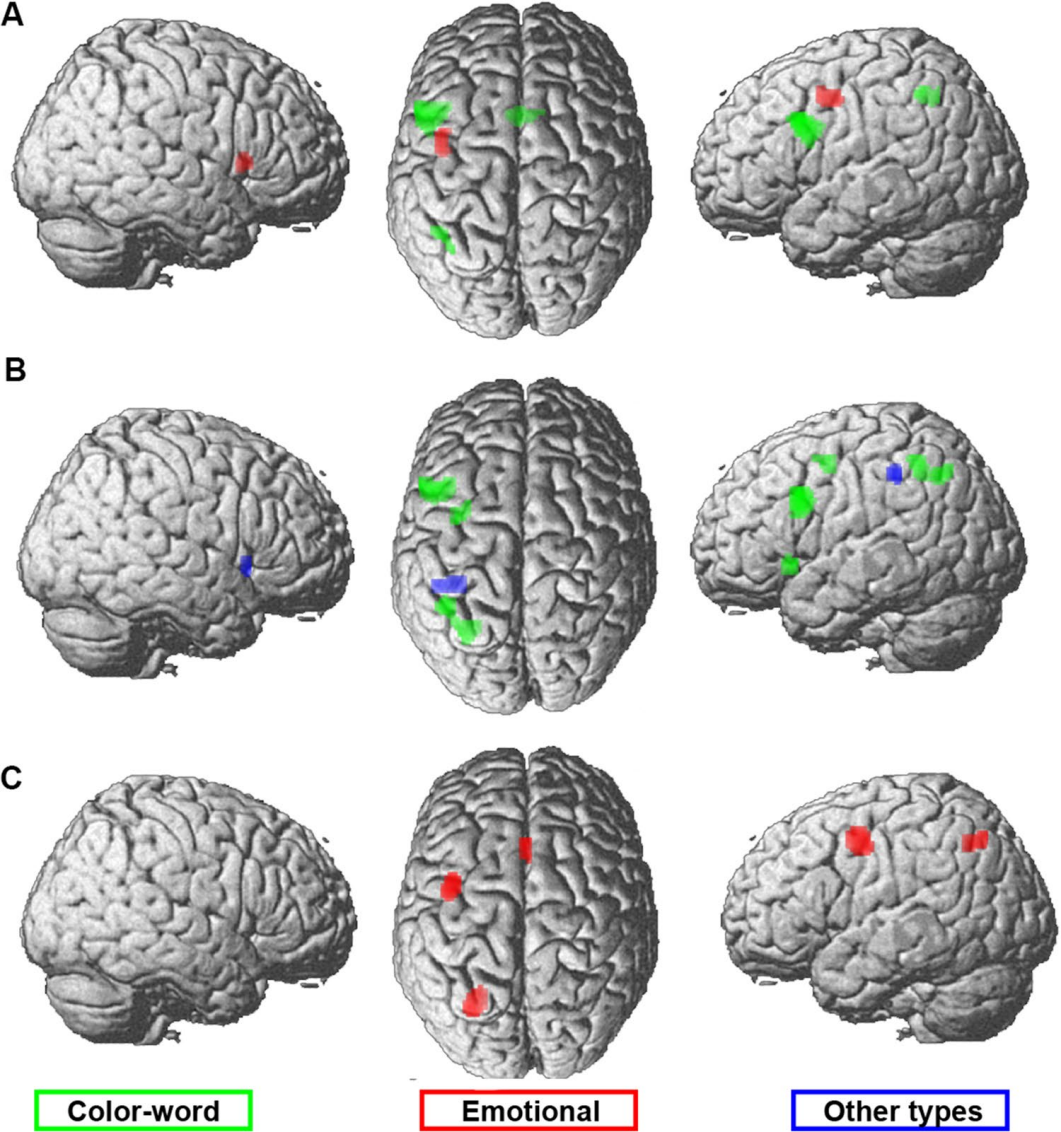
Differences in convergence between the three stimulus material-specific meta-analyses. Contrast analyses between **A** color-word and emotional task versions, **B** color-word and other Stroop versions and **C **emotional and other Stroop versions. Stronger convergence for color-word versions is shown in green, for emotional versions in red, and for other Stroop versions in blue. The color intensity of the renderings (the first three columns) reflects the distance from the brain surface (lower intensity denoting further distance from the surface)

The conjunction analysis across color-word and emotional Stroop tasks revealed that both recruit bilateral inferior/middle frontal gyrus, right aINS, left mIPS, dorsal premotor cortex (dPMC), and pmFC (Fig. 6 in yellow). Emotional Stroop showed stronger convergence in the left ventral dorsal premotor cortex (dPMC) and right aINS (Fig. 7A in red), while color-word revealed more consistent recruitment of left middle frontal gyrus, pre-SMA, and left mIPS (hIPS1, Fig. 7A in green).

The conjunction analysis across color-word and other types (Fig. 6 in cyan) of Stroop did not reveal any additional overlap beyond that found in the three-way conjunction. For color-word Stroop tasks, stronger convergence was found in the left inferior/middle frontal gyrus and IFJ, left mIPS (hIP3), left dPMC, and left aINS/orbitofrontal cortex (Fig. 7B in green), while other types of Stroop showed stronger convergence in left aIPS/IPL and right aINS (Fig. 7B in blue).

Emotional and other types of Stroop tasks did not reveal any additional overlap beyond that found in the three-way conjunction. Emotional Stroop tasks more consistently recruited left mIPS (hIP3) and left ventral dPMC (Fig. 7C, in red), whereas no stronger convergence was found for other types of Stroop tasks.

In summary, all three types of stimulus material elicited consistent interference-related activity in regions of the salience network, in particular the pmFC and the right aINS. Emotional Stroop was found to be specifically associated with ventral dPMC and color-word Stroop with the left middle frontal cortex, while other types of Stroop showed generally less convergence as well as stronger convergence in aIPS only in comparison to the color-word version.

### Follow-up Analysis: Meta-analyses of the Behavioral Stroop Effect

Table 2, part A, provides an overview of all analyses and the results. For the analyses of the behavioral Stroop effect, we first estimated a mean effect size across all experiments. Then, two meta-regression models were calculated, one testing the effects of control condition, presentation design, and additional cognitive demand for color-word Stroop only and one testing the moderator stimulus material for experiments using a congruent condition as control and without additional cognitive demand. At last, sample biases and outlier analyses were performed and all analyses were repeated without outliers.

#### Intercept-Only Model

To estimate the aggregated effect size (ES) estimate across all experiments (77 studies with 68 independent studyIDs and 164 ES), an intercept-only model was calculated, yielding $\overline{g}$=0.64 (SE = 0.04; 95% confidence interval (CI) = 0.56–0.73; degrees of freedom (dfs) = 65.7, *p* < 0.0001).

#### Meta-regression Across Color-Word Stroop Experiments

**Fig. 8:**
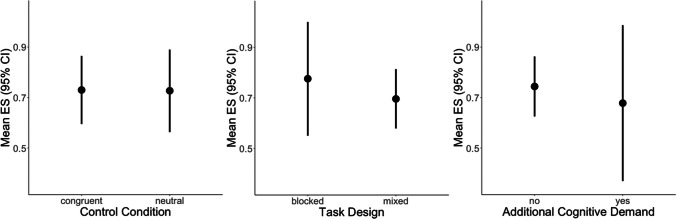
Illustration of the effect sizes of the color-word Stroop task for each (level of) the moderators control condition, presentation design, and additional cognitive demand. No significant effects were found for any of the moderators

The meta-regression model testing the moderators control condition, presentation design, and additional demand indicated that none of the moderators had a significant effect (39 studyIDs with 112 ES; Table 3; see also Fig. 8 for mean effect sizes for each condition).

**Table 3:** Results of the meta-regression across color-word Stroop tasks revealing no significant effect of the moderators control condition, presentation design, and additional cognitive demand

	Estimate	SE	*t*	df	*p*	95% CI
Intercept	0.81	0.12	6.7	15.5	< 0.0001	0.56–1.07
Control condition	− 0.02	0.09	− 0.25	28.6	0.805	− 0.20–0.15
Design	− 0.09	0.13	− 0.74	28.6	0.465	− 0.35–0.17
Demand	− 0.09	0.15	0.57	12.9	0.581	− 0.42–0.24

Additional analyses that additionally model the moderators type of neutral control condition, interaction between design and control condition, response modality, and number of different colors can be found in the supplement (tables S5 and S6). These analyses did not reveal any significant effects.

#### Meta-regression Testing for Effects of the Stimulus Material

**Fig. 9:**
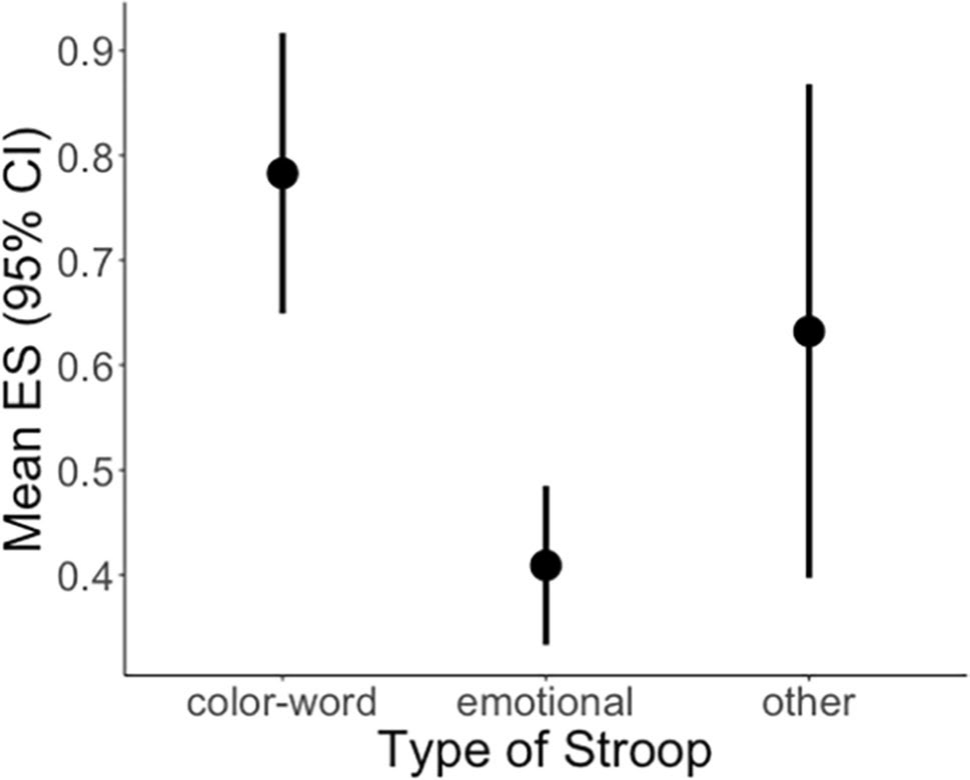
Illustration of the effect sizes of Stroop interference for the different types of Stroop stimulus material, with a smaller effect for emotional than color-word material

The meta-regression (53 studyIDs with 97 ES) testing the moderator “stimulus material” revealed a significant effect (Wald test for testing the overall effect: *F*_2, 31.7_ = 13.5, *p* < 0.001; Fig. 9 for mean effect sizes of the levels of the moderator). Emotional Stroop tasks exhibited a significantly smaller mean effect size than color-word versions ($\overline{gEMO}$=0.41, $\overline{gCW}$= 0.78; *t*_26.4_ =  − 5.1, *p* < 0.001) but not other types of Stroop tasks ($\overline{gOther}$=0.63, *t*_26.4_ = 1.92, *p* = 0.065). A model using the color-word version as a baseline revealed that color-word and other types of Stroop tasks did not differ significantly (*t*_32.3_ = 1.16, *p* = 0.26).

#### Sample Bias

Testing for sampling bias by calculating a meta-regression including SE as a moderator revealed a significant effect for SE (beta = 4.50; SE = 0.86, *t*_19.1_ = 5.21, *p* < 0.001), indicating bias in the data. Outlier analyses revealed influential cases for both models (color-word Stroop model: effect size from Kim et al., 2014, and one effect size from Bench et al., 1993; stimulus material model: two effect sizes from Matthews et al., 2004). Removing those cases did not change any effects of the moderators for color-word Stroop only. When removing outliers from the meta-regression testing for the type of stimulus material, not only emotional Stroop tasks (*t*_26.4_ =  − 5.2, *p* < 0.001) but also other types (*t*_30_ =  − 2.2, *p* = 0.03) differed significantly from color-word stimulus material.

Summarizing the results of the meta-analyses across behavioral Stroop effects, none of the factors investigated (control condition, design, additional demand) had an effect on the size of the incongruence effect during the color-word Stroop task. However, the type of stimulus material significantly modulated the size of the effect, with emotional and other Stroop-type tasks (after removing outliers) leading to smaller interference than observed for the color-word Stroop task version.

## Discussion

We performed meta-analyses to investigate the modulatory effects of different task variations on Stroop-type interference as reflected in performance and brain activation. When we investigated the classic color-word Stroop task only, we observed no significant behavioral effects of any variations. However, when comparing to other Stroop-like tasks, we found significant modulations of the behavioral Stroop effect by stimulus material, with the strongest behavioral interference effect observed for the classic color-word Stroop version. On the neural level, left-sided regions of the multiple-demand (MD) network were consistently recruited across variations of the color-word Stroop tasks, with some differences in convergence for right-sided MD regions as well as the fusiform gyrus and orbitofrontal cortex. In addition, when looking at stimulus material other than that used in the color-word and emotional Stroop variants, the neural correlates of the interference effect differed. These results lead to different conclusions about the behavioral and neural interference effect with different neural mechanisms being associated with variations in experimental setup which, however, lead to similar behavior. The current meta-analytic study thus provides crucial insights for clinical and cognitive neuroscience as differences in neural mechanisms may affect the sensitivity of a specific task version in detecting a particular cognitive (dis)ability and highlights the notion that it is quite difficult to generalize effects beyond the specific task version used.

### The Behavioral Stroop Interference Effect

Aggregation of effect sizes of the behavioral interference effect revealed a medium effect size across all conditions for Stroop tasks conducted in neuroimaging environments. Importantly, this effect was quite consistent (i.e., similarly strong) across different variations of color-word Stroop tasks, as effect sizes were independent of the control conditions, presentation design, and the presence of additional cognitive demand. These results side with the idea that the exclusive driver of the behavioral Stroop effect is the higher-order conflict that is common to all Stroop-type tasks, which arises when two semantically related stimulus dimensions are incongruent to each other. The influence of variations of the classic color-word version on Stroop-type interference has been rarely studied systematically, and conclusions could often only be drawn indirectly. For example, we assumed that the type of control condition (congruent vs. neutral) would affect the interference effect size, as benefits for reaction time (RT) from facilitation by congruent (but not neutral) stimuli seem intuitive and have been previously reported. However, it has also been argued that the facilitation effect is much smaller than the interference effect (Macleod, 1991). Our results now add to this discussion by indicating that the presumably small effects of facilitation per se do not consistently lead to a significantly stronger Stroop interference effect when incongruent conditions are compared to congruent rather than neutral conditions.

For blocked versus mixed designs, previous studies reported inconsistent results, with some pointing to stronger interference effects for mixed (Floden et al., 2011) and others for blocked presentations (Hasshim & Parris, 2018; Salo et al., 2001). Our results, summarizing effects found across many studies, now indicate that none of the previously reported effects reported for design and control condition is generalizable and that the kind of control condition, presentation design, and cognitive demand has no impact on the size of the Stroop effect for color-word tasks in the neuroimaging settings.

The only variation that did make a difference was the type of stimulus material used, with the interference effects of emotional face Stroop tasks being smaller than that of color-word ones. Additionally, when removing outliers, the analysis also revealed a difference between other types and color-word Stroop, with smaller effects observed for the former. This contrasts with Chechko et al. (2013), who reported stronger effects for emotional than non-emotional face-word versions of the task. We assume that the effect reported in Chechko et al. (2013) cannot easily be generalized to other task types but is rather specific to the comparison of emotional to non-emotional face-word Stroop task versions. Unfortunately, we only had a very limited amount of non-emotional face Stroop experiments in our sample and thus included non-emotional face experiments in the mixed category “other types,” precluding separate analysis. In general, the results of the few previous studies that compare effects between tasks are quite inconsistent, with some reporting differences (Banich et al., 2000; Capizzi et al., 2017; Chechko et al., 2013; Hilbert et al., 2014), but others not (Mitchell, 2005; Zoccatelli et al., 2010). Importantly, those previous studies differ in the specific tasks that have been compared as well as the settings (neuroimaging vs. outside of the scanner setting). By aggregating the pertinent neuroimaging literature, our findings further our understanding of which task variations do or do not influence the Stroop interference effect when performed inside the scanner, indicating that different variations of the color-word Stroop task lead to comparable behavioral effects, while the emotional and other Stroop-type tasks should be treated differently.

### The Multiple-Demand (MD) Network During Stroop Interference Processing

The MD network, consisting of the lateral frontal cortex, anterior insula (aINS)/frontal operculum, and intraparietal sulcus (IPS) as well as pre-supplementary motor area (pre-SMA) and dorsal anterior cingulate cortex (hereafter jointly labeled pmFC), has been found to be associated with various cognitive challenging tasks. It is therefore assumed to play a major role in establishing general mental control programs across a wide range of tasks with demand for top-down control (Duncan, 2010, 2013). Accordingly, we would have assumed that the higher-order conflict that all Stroop-type tasks have in common, i.e., the overlap of the two dimensions of the stimulus (Kornblum & Stevens, 2002), is reflected by a consistent involvement of this system. In line with this reasoning and with previous meta-analytic findings on interference processing (Chen et al., 2018a; Cieslik et al., 2015; Huang et al., 2020), the present study found convergence in regions of this brain system across various experiments of the large family of color-word and emotional picture-word Stroop tasks. However, while some of these regions were quite consistently found in (almost) all analyses, there were other regions of the MD network that were modulated by control condition and especially by presentation design and stimulus material. Thus, our study provides a more fine-grained differentiation of the MD system, with some regions being more tightly associated with higher-order conflict that all included experiments have in common (resulting from the overlap of two stimulus dimensions) and others being more related to conflict specific to some task variations.

### Influence of the Control Condition

The regions revealed by the two meta-analyses across I > C and I > N contrasts, respectively, are rather similar, which is confirmed by the conjunction analysis showing convergence in bilateral aINS and lPFC, left IPS, and pmFC. The only difference between the two resulting networks was that most of the MD regions of convergence for I > N were, in general, larger.

The more extended and stronger convergence of MD regions for experiments that contrast interference processing against a neutral condition might point to task conflict (MacLeod & MacDonald, 2000) in congruent conditions that are absent/reduced from neutral ones, effectively reducing the difference between the congruent and the target conflict condition (i.e., the incongruent one). Task conflict refers to the simultaneous activation of two or more different task sets, i.e., besides preparation for the task set of color identification, also, the task set of word reading is activated. This concurrent preparation of the two different task sets occurs not only in incongruent conditions but also in congruent ones (Littman et al., 2019; MacLeod & MacDonald, 2000; Parris, 2014). In contrast, neutral conditions using non-words activate the task set of word reading to a lesser extent than congruent and incongruent words (Keha & Kalanthroff, 2023; Parris, 2014). Therefore, task conflict in congruent conditions might potentially also recruit MD regions to some extent, resulting in smaller differences in recruitment of those regions between incongruent and congruent conditions. However, Parris et al. (2019) did not find any evidence for regions associated with task conflict. Additionally, while others have linked task conflict in color-word Stroop versions especially to the pmFC region (in particular cingulate parts; MacLeod & MacDonald, 2000), our results indicate that rather the whole MD network might be involved. Importantly, when separating the experiments of I > N into those that used non-words as neutral conditions (letters or symbols—therefore, little task conflict) and those that used neutral words (including task conflict), results were similar to when combining all neutral control conditions. Only the left aINS was selectively found in the analysis across neutral control conditions using symbols/letters (vs. neutral words). Thus, task conflict can potentially explain stronger convergence in the left aINS when using a neutral control condition compared to a congruent one but cannot fully explain the broader recruitment of other regions of the MD system when controlling against a neutral condition.

In summary, our results provide some evidence for potentially different mechanisms involved in processing congruent versus neutral conditions, leading to a generally stronger difference in the recruitment of MD regions between incongruent and neutral conditions, as compared to contrasts between incongruent and congruent conditions, which cannot, however, be fully attributed to task conflict.

### Influence of Presentation Design

Looking at the results of the two meta-analyses of experiments presenting conditions in blocked or mixed fashion, respectively, reveals that the Stroop effect is associated with convergence in more brain regions when mixed compared to blocked designs are used. Both analyses showed convergence in left lPFC, IPS, aINS, and pmFC, but experiments with mixed (vs. blocked) presentation showed additional convergence in right lPFC, right aINS, and left FFG as well as a generally stronger convergence of all MD regions (with the exception of more anterior pmFC showing stronger convergence in blocked designs). This result is in line with a previous study that found stronger effects for mixing (vs. blocking) conditions in the MD network in Stroop (Floden et al., 2011) as well as flanker tasks (Marini et al., 2016) and more left-sided involvement in a Stroop task with blocked presentation of the Stroop (albeit without not direct comparison to a mixed version, Leung et al., 2000).

The more bilateral involvement for mixed designs might be explained by difficulty and mental load, with increasing recruitment of the MD system as well as involvement of regions of the less dominant (i.e., right for verbal tasks like color-word Stroop) hemisphere as load increases (Shashidhara et al., 2019). Thus, right-lateralized regions might be co-recruited in more challenging conditions, as it is when conditions are mixed, to overcome increased interference. Interestingly, this seems to lead to similar behavioral interference effects in blocked and mixed designs, despite greater difficulty in the latter. It might be that the recruitment of right-sided regions compensates for mixing costs.

In general, blocked and mixed designs differ in the amount and type of carry-over effects, with blocked designs exhibiting stronger adaptation effects and proactive control (Hasshim & Parris, 2018). For solving color-word Stroop trials in a blocked design, interference can be anticipated and appropriate control settings can be implemented in advance and maintained across the whole block, which is beneficial. Additionally, Kalanthroff et al. (2018) suggested that task conflict is particularly apparent when proactive control (maintenance of attention/control) is low. Stronger (and more bilateral) convergence in the MD system in mixed designs (see Fig. 5) in the present study might therefore be due to stronger task conflict in mixed designs (as proactive control is lower compared to blocked presentation). However, as discussed before, our results regarding the type of control condition do not support the effects of task conflict on most regions of the MD system, except for the left aINS. Interestingly, left aINS was also found to show stronger convergence in mixed than in blocked designs. Thus, together with the results of the control condition, the findings of blocked versus mixed design point to the left aINS in playing a major role in Stroop-type task conflicts.

Another explanation for the differences between blocked and mixed designs might be the modeling of the hemodynamic response. While for mixed presentations, analysis is event-related where every experimental event is convolved with the hemodynamic response model, the analysis of blocked conditions convolves the whole boxcar time course. It might be the case that some regions show an early activation and a late deactivation, which could lead to a cancellation of the response in blocked designs, while event-related analysis might not capture the late response (i.e., the deactivation) and thus activation is predominantly found (Meltzer et al., 2008). However, why this should be especially the case for right-sided regions remains an open question for now.

The pattern of left-sided vs. more bilateral involvement in blocked versus mixed designs, respectively, might explain divergent findings in clinical studies, in which right-sided aberrations are more likely to be found when using a mixed compared to a blocked design. Thus, blocked designs might be particularly sensitive for left-sided impairments, while right-hemispheric dysfunctions might only become apparent when conditions are presented in a mixed format. This is in line with the findings of human lesion studies pointing to especially lesions of lPFC on the left side being associated with Stroop performance decline (Cipolotti et al., 2016; Glascher et al., 2012; Perret, 1974) and the fact that clinical studies primarily use original (clinical) forms of the Stroop task in which word stimuli are presented in a blocked, list-wise format.

In addition to the generally stronger and more bilateral convergence in the MD for mixed (vs. blocked) presentation designs, we found that the left FFG was more consistently reported when conditions were mixed. The cluster in the FFG corresponds to the fusiform word area (Cohen & Dehaene, 2004; Cohen et al., 2000, 2002; Lorenz et al., 2017), a region involved in word reading (Cohen & Dehaene, 2004; Cohen et al., 2000, 2002). During the performance of the Stroop task, one would expect neural suppression of this region to inhibit word reading (Polk et al., 2008). The fact that we did not find convergence for blocked designs could indicate that suppression worked and word reading was successfully inhibited when the same conditions were presented in a row. However, in mixed designs, suppression might be more difficult, especially in conditions in which word meaning and ink color do not match.

Additionally, we found stronger convergence for blocked designs in the right orbitofrontal cortex, often referred to as part of the ventrolateral prefrontal cortex (vlPFC). Importantly, a study by Egner (2011) suggested that the right vlPFC plays a key role in conflict adaptation by showing that the strength of the behavioral conflict adaptation effects increases with more activation in this region. However, the vlPFC was only found when using an interindividual differences approach, while analyses across groups point to the dorsolateral prefrontal cortex (dlPFC) (Egner & Hirsch, 2005; Egner et al., 2008). The vlPFC might therefore be the main source of regulation processes, while (right) dlPFC is only recruited additionally if adaptation does not work or is difficult (i.e., in poor performers; Egner, 2011). This assumption could explain why vlPFC, but not right dlPFC, is found for blocked designs: in a blocked presentation mode, behavioral adaptation is easier, and after a few trials, even participants with difficulties in adapting to the conflict may manage to adjust, and therefore, no further recruitment of right dlPFC is needed. The results of the current meta-analysis might therefore reflect carry-over effects and, in particular, adaptation induced by blocking conditions, and they additionally indicate that blocked designs may be more sensitive to adaptation effects that are only apparent in mixed designs when using individual-differences approaches.

Furthermore, contrast analyses revealed a differentiation within the pmFC, with stronger convergence in pre-SMA for mixed presentation designs, whereas, for blocked designs, more consistent activation was found in a more anterior cluster. pmFC convergence found in the conjunction was located between those two clusters. This differentiation in pmFC is quite interesting, as it has been previously found that proactive (anticipation and prevention of interference before it occurs) and reactive (transient detection of interference after it occurred) controls were associated with different parts within the pmFC, with a more anterior region linked to proactive and a more posterior one to reactive control (Burgess & Braver, 2010). Assuming that proactive and reactive controls differ in blocked and mixed designs, with stronger proactive control in blocked and reactive one in mixed designs, this differentiation in the pmFC might therefore reflect this distinction between these two control mechanisms.

In summary, our results show that context alters interference-related processing in the Stroop task, as reflected in the differential recruitment of the MD network during blocked versus mixed presentation modes, potentially leading to a similar behavioral Stroop effect for both designs. Stronger and bilateral recruitment of the MD network in mixed designs might reflect increased cognitive load, while right vlPFC convergence for blocked designs could be the result of adaptation. Additionally, together with the results on the effects of the type of control condition, our findings point to the left aINS as playing a role in task conflict. Finally, a differentiation within pmFC between the two task designs most likely reflects differences in the exertion of pro- and reactive control.

### Influence of Stimulus Material

This meta-analytic study showed that the stimulus material used to induce Stroop-type conflict (i.e., color-word, emotional, or other Stroop-like tasks) has some impact on which interference-related regions are consistently found across experiments. The only overlap between all three categories of Stroop tasks employed in this study was found in the pmFC (pre-SMA and anterior midcingulate cortex) and right aINS. Both regions are part of the MDN but have in particular been described as forming the salience network (Seeley et al., 2007), playing a major role in detecting salient stimuli and implementing and maintaining the appropriate task set via initiating switches between task-relevant and task-irrelevant networks (Dosenbach et al., 2006; Menon & Uddin, 2010; Sridharan et al., 2008). Consistent involvement of the salience network across all three material-specific categories of the Stroop tasks therefore indicates that the detection of (salient) stimulus conflict and the (re)activation of the instructed task set to overcome inadequate response tendencies are implemented in a similar, material-independent way.

Interestingly, our results point to some distinctions within left IPS with regard to stimulus material. In particular, color-word and emotional Stroop experiments primarily converged in caudal parts of the anterior IPS (in particular hIP1, hIP3, and hIP6; Choi et al., 2006; Scheperjans et al., 2008a, 2008b), which we name “middle IPS” (mIPS). In contrast, for other types of Stroop tasks, convergence was mainly and more consistently found in the most anterior part of IPS (hIP2; Choi et al., 2006), as compared to color-word Stroop versions. Studies that investigate the regions associated with numerical processing often reported regions that overlap with the cluster found in our meta-analyses for other types of Stroop tasks. Zago et al. (2008) reported a stronger role of anterior IPS in working memory tasks with numerical stimuli, as compared to verbal syllables, and Vogel et al. (2017) also pointed to anterior IPS as involved in symbolic number processing across modalities. In general, IPS is held to play a strong role in attentional shifting and stimulus–response mapping (Cieslik et al., 2015; Worringer et al., 2019). During the Stroop task, IPS involvement therefore might reflect the allocation of attention to, and/or the selection of specific stimulus characteristics (Cieslik et al., 2015) like color, location, or numerical quantity of a stimulus. Our results now indicate that within IPS, this attentional shifting might be implemented in a material-specific way. Importantly, when looking at the experiments that contribute to the aIPS cluster in the meta-analysis across other types of stimulus material, all but one experiment used a numerical or counting Stroop task version. Thus, the differentiation within left IPS may point to a shift of recruitment from middle IPS for allocation of attention to color and emotional expression of the stimulus, to more anterior IPS for attentional shifts to magnitude and quantitative properties (like numerical magnitude or font size).

When compared to the other two types of stimulus material, stronger convergence was found in the ventral dorsal premotor cortex (dPMC) for emotional picture-word Stroop tasks and in lPFC for color-word Stroop. In terms of the Stroop task, it has been shown that left dPMC is specifically associated with the regulation of perceptual conflict, while the lateral frontal cortex is more involved in conflict detection and response conflict (Kim et al., 2012). Given the stronger consistency of dPMC across experiments in emotional interference, our results might therefore indicate that resolving perceptual conflict is more relevant in emotional than color-word or other Stroop variants. In turn, stronger convergence for color-word Stroop versions in lPFC indicates that for this task variant, resolution of response conflict may play a stronger role than for the other two categories of stimulus material.

Additionally, it has been suggested that the lateral prefrontal cortex is especially involved when there are differences in the automaticity of the two competing dimensions (Banich, 2019). Importantly, for the color-word Stroop tasks, interference effects only arise when the word meaning is the task-irrelevant dimension, but not when the color of the ink must be ignored (Stroop, 1935). Therefore, the automaticity of the two competing dimensions is unbalanced, leading to a stronger recruitment of the lateral prefrontal cortex. In contrast, for some other Stroop versions but also emotional Stroop, this difference in automaticity of the two competing dimensions is less pronounced. In the numerical Stroop version, for example, behavioral interference effects are observed when either the physical size or the numerical magnitude is the task-irrelevant dimension (Huang et al., 2012; Tang et al., 2006). Similarly, with emotional Stroop tasks, interference effects are observed for both the identification of facial expression and word reading (Bayer et al., 2018). Thus, the stronger convergence observed in lPFC for color-word Stroop tasks might be due to the fact that there is more competition between the automaticity of the two competing dimensions and, therefore, more need for top-down control.

In summary, our meta-analytic results regarding the impact of different types of stimulus material on the neural correlates of Stroop-like interference processing highlight the salience network as a core system for dealing with such conflicts. Additionally, recruitment of the MD network is to some extent material-specific, suggesting that partially different control mechanisms are recruited for different Stroop variants.

### Limitations and Outlook

It must be noted that the behavioral effects reported here can only be generalized to settings typical of neuroimaging experiments. Previous work points to differences in reaction time for studies performed in and outside the scanner (Koch et al., 2003; Koten et al., 2013; van Maanen et al., 2016). Therefore, outside the scanner environment, these effects might look different, and a next step would be to test the influence of task variations on the behavioral Stroop effect in standard laboratory and clinical routine settings.

Furthermore, it should be mentioned that the “other” category of stimulus material comprised more than one Stroop variant. This heterogeneity was due to the limited number of available fMRI experiments for each version, which did not allow for forming more material-specific categories. Therefore, some effects (or their absence) observed for this mixed category of other types of Stroop tasks might be driven by only a specific subgroup of tasks. For the brain regions where convergence was found, our analysis of study contributions indicated that there was not a specific type of task driving the results. However, for regions where no convergence was found in the category of other types of Stroop (e.g., dlPFC), it is unclear if this is potentially due to heterogeneity or one specific type of Stroop. Nevertheless, if the Stroop effect was independent of stimulus material, we would still expect convergence in the same regions as observed for color-word and emotional Stroop versions. Therefore, future studies (single fMRI studies directly comparing different task variants or meta-analyses performed once more experiments have become available) should specifically compare different task types now subsumed in this mixed group.

Furthermore, the Eggers regression test indicated that there is a sampling bias in the reported effect sizes of the behavioral Stroop effect. However, this is not surprising as we pre-selected the ES by focusing on published neuroimaging studies. Additionally, our focus was not on determining the overall effect size. Rather, we were mainly interested in examining whether and how different moderators influence the size of the Stroop interference effect in task versions implemented in neuroimaging settings. We therefore already assessed factors that might explain the heterogeneity of effects and sampling bias.

Additionally, it has to be acknowledged that brain signals can be confounded by reaction time variations (Mumford et al., 2023) and that presumably conflict-related activations can be explained by the time spent on the task. Thus, some neural effects of task variations found in our meta-analysis could potentially not necessarily reflect differences in conflict processing per se but rather be an effect of reaction time.

Besides the stimulus material, presentation design, control condition, and additional cognitive demand, there also are other factors (like the probability of different conditions, stimulus set size, response modality) that might influence the size of the Stroop effect as well as its neural correlates (for review, see Macleod, 1991). However, we only investigated task and experimental variations that were used in a sufficient number of studies.

## Conclusion

Overall, our results suggest that different neural mechanisms are associated with variations in the experimental setup of Stroop-like tasks, which, however, lead to similar interference effects on the behavioral level. In line with the view of a “many-to-one mapping” (Westlin et al., 2023), this suggests that the seemingly unitary behavioral costs of Stroop-type conflicts may arise from partly different processing mechanisms, depending on contextual factors. This is especially true for differences in presentation design, where mixed presentations (as compared to blocked designs) recruit the MD network more strongly and more bilaterally, as well as for variations in stimulus material, which differ in the recruitment of parietal, lateral frontal, and dorsal premotor cortex. This therefore highlights that Stroop-type neuroimaging experiments as well as applications of Stroop-like tasks in clinical, occupational, or other diagnostic settings should be carefully planned and interpreted, as task variations influence the set of brain regions recruited. Although behavioral interference effects were, at the level of group averages, hardly affected by task variations, the differences in neural mechanisms may make a given task version more or less sensitive to particular cognitive ability differences. Information on differences in the brain regions recruited for different task versions is thus of high relevance for clinical neuropsychology, as the version used should recruit the brain region of interest (e.g., mixed presentation mode is recommended when not only left-sided dysfunctions are to be detected). In cognitive neuroscience, in turn, the divergence of behavioral and neuroimaging effects might render it particularly difficult to find brain-behavior relationships that are generalizable across different task versions. Ultimately, our results question the meaningfulness of using “Stroop task” in neuroimaging research and applied settings as an umbrella term for such a wide variety of flavors.

## Supplementary Information

Below is the link to the electronic supplementary material.

Supplementary file1 (DOCX 32 KB)

Supplementary file2 (DOCX 25 KB)

Supplementary file3 (PDF 1.63 MB)

## Data Availability

Analysis code of neuroimaging and behavioral meta-analyses can be found on the open science framework (OSF; https://osf.io/dt3kj/?view_only=Scheperjans97bb53574583b1a0dda978f7f341); result files of the ALE meta-analysis are available at ANIMA (https://anima.fz-juelich.de/).
