# Supplementary material for: Not All Stroop-Type Tasks Are Alike: Assessing the Impact of Stimulus Material, Task Design, and Cognitive Demand via Meta-analyses Across Neuroimaging Studies
Source: Neuropsychol Rev. 2024 Sep 12;35(3):449–82. doi: 10.1007/s11065-024-09647-1 (PMC12602573; doi:10.1007/s11065-024-09647-1)
Supplement: Supplementary file 3 — Supplementary file3 (PDF 1.63 MB) [file 11065_2024_9647_MOESM3_ESM.pdf]

Supplemental material:

Not all Stroop-type tasks are alike: Assessing the impact of stimulus material task design and cognitive demand via meta-analyses across neuroimaging studies

Veronika I. Müller [1,2,*], Edna C. Cieslik [2,1], Linda Ficco[3,4], Sandra Tyralla[5], Amir Ali Sepehry[6], Taraneh Aziz-Safaie [2,1], Chunliang Feng[7], Simon B. Eickhoff [1,2], Robert Langner [2,1]

1 Institute of Neuroscience and Medicine, INM-7, Research Centre Jülich, Germany; 2 Institute of Systems Neuroscience, Medical Faculty, Heinrich Heine University, Düsseldorf, Germany; 3 Department of General Psychology and Cognitive Neuroscience, Friedrich Schiller University, Jena, Germany; 4 Department of Linguistics and Cultural Evolution, International Max Planck ResearchSchool for the Science of Human History, Jena, Germany; 5 Institute for Experimental Psychology, Heinrich Heine University, Düsseldorf, Germany; 6 Counseling and Clinical Psychology Programs, Adler University (Vancouver Campus), Vancouver, Canada; 7 Key Laboratory of Brain, Cognition and Education Sciences, South China Normal University, Guangzhou, China; *corresponding author: v.mueller@fz-juelich.de

Table S1

Description of the studies and the respective experiments selected for inclusion in the neuroimaging meta-analyses

Study	N	Stimulus Material	Control Condition	Design	Additional demand	Space	Source of Coordinates	Meta control	Meta design	Meta demands	Meta Material
Agostini et. al. 2017	17	CW	Congruent	Blocked	Yes	MNI	Author sent result table	-	-	D	-
Almdahl et al., 2021	60	Emotional	Congruent	Mixed	No	MNI	Author sent result image	-	-	-	E
Ansari et al., 2006	14	Other - Numerical	Congruent	Mixed	No	TAL	Table 2, main effect of congruency	-	-	-	O
Adleman et al., 2002	11	CW	Neutral	Blocked	No	TAL	Table 2, Young adult group average	N	B	ND	-
Bang et al.., 2016	43	Emotional	Congruent	Mixed	No	MNI	Table S1, incongruent> congruent	-	-	-	E

Banich et al., 2001	14	CW	Neutral	NA	No	TAL	Text p. 9, standard incongruent color-word versus neutral word	N	-	ND	-
Barkley-Levenson et al., 2018 A	105	CW	Congruent	Mixed	No	MNI	Table 3, incongruent > congruent	C	M*	ND*	CW
B	105	CW	Neutral	Mixed	No	MNI	Table 3, incongruent > neutral	N	M*	ND*	-
Basten et al., 2011	46	CW	Congruent	Mixed	Yes	MNI	Table 3	-	-	D	-
Bayer et al., 2018	15	Emotional	Congruent	Mixed	No	MNI	Table 1, attend face congruent vs incongruent	-	-	-	E
Becker et al., 2008	17	CW	Congruent	Mixed	No	TAL	Table 2, controls	C	M	ND	CW
Bench et al., 1993 A	6	CW	Neutral	Blocked	No	TAL	Table 2, Experiment 1, Stroop vs crosses; stroop vs. neutral	N	B	ND	-
B	6	CW	Neutral	Blocked	No	TAL	Table 2, Experiment 2, Stroop vs crosses	N	B	ND	-
Bohle, 2016	20	Other-picture	Congruent	Mixed	No	MNI	Table 6.4, Stroop effect (related >same)	-	-	-	O
Brass et al., 2005	10	CW	Congruent	Mixed	No	TAL	Table 1	C	M	ND	CW
Carter et al., 1995							Table 3, increase				

A	14	CW	Congruent	NA	No	TAL		C	-	ND*	CW
B	14	CW	Neutral	NA	No	TAL	Table 1, increase	N	-	ND*	-
Chechko et al., 2009	18	Emotional	Congruent	Mixed	No	MNI	Table S1, positive Bold response	-	-	-	E
Chechko et al., 2012 A	24	Other-picture	Congruent	Mixed	No	MNI	Table 6	-	-	-	O
B	24	Emotional	Congruent	Mixed	No	MNI	Table 5	-	-	-	E
Chechko et al., 2013 A	18	Emotional	Congruent	Mixed	No	MNI	Table 2	-	-	-	E
B	18	Other-picture	Congruent	Mixed	No	MNI	Table 5	-	-	-	O
Chen et al., 2018	36	CW	Congruent	Mixed	No	MNI	Table 2, Stroop	C	M	ND	CW
Chen et al., 2023	30	Emotional	Congruent	Blocked	No	MNI	Supplementary table 1, Control	-	-	-	E
Chen, Meng et al., 2023	41	CW	Congruent	Mixed	No	MNI	Table 2	C	M	ND	CW
Coderre et al., 2008 A	9	CW	Congruent	Blocked	No	TAL	Table 2, coordinates with positive t value	C	B*	ND*	CW
B	9	CW	Neutral	Blocked	No	TAL	Table 4, coordinates with positive t value	N	B*	ND*	-
Coderre et al., 2013 A	14	CW	Congruent	Mixed	No	MNI	Table 1, 0 ms SOA, Stroop effect	C	M*	ND*	CW
B	14	CW	Neutral	Mixed	No	MNI	Table 1, 0 ms SOA, Interference effect	N	M*	ND*	-

DeVito et al., 2012	12	CW	Congruent	Mixed	No	MNI	Supplement table 1, HC Session 1	C	M	ND	CW
Fan et al., 2003	12	CW	Congruent	Mixed	No	MNI	Table 2	C	M	ND	CW
Fan et al., 2017	12	Other-Counting	Congruent	Mixed	No	MNI	Table VI, Healthy control group	-	-	-	O
Fechir et al., 2010	16	CW	Congruent	Blocked	No	MNI	Table 2, Interference > Congruent	C	B	ND	CW
Fedeli et al., 2022	42	Other-Counting	Congruent	MIxed	No	MNI	Table S1, Stroop Incongruent > Congruent	-	-	-	O
Fleury et al., 2014	12	Emotional	Congruent	Mixed	No	MNI	Table 3	-	-	-	E
George et al., 1994	21	CW	Neutral	Blocked	No	TAL	Table I, standard stroop	N	B	ND	-
Ghavidel et al., 2020	18	CW	Congruent	Blocked	No	MNI	Coordinates sent by the authors	C	B	ND	CW
Godinez et al., 2016	9	Emotional	Congruent	Blocked	No	MNI	Table 5, control co-twins	-	-	-	E
Gianaros et al., 2008	32	CW	Congruent	Blocked	Yes	MNI	Supplemental table 2	-	-	D	-
Grandjean et al. 2012 – A	25	CW	Neutral	Mixed	No	MNI	Table 2	N	M*	ND*	-
Grandjean et al., 2013- B (same subjects as 2012)	25	CW	Congruent	Mixed	No	MNI	Table 2	C	M*	ND*	CW
Hart et al., 2010	14	Other-Counting	Congruent	Mixed	No	MNI	Table 2, Incongruent > Congruent	-	-	-	O
Hassel et al., 2020	36	Emotional	Congruent	Mixed	No	MNI	Supplementary Table 1,	-	-	-	E

							Incongruent minus congruent, coordinates with p<0.001				
Hinault et al., 2019	22	Other-Numerical	Congruent	Mixed	No	MNI	Table 2	-	-	-	O
Hoogeveen et al., 2020	193	Other-picture	Congruent	Mixed	No	MNI	Downloaded from neurovault	-	-	-	O
Hough et al., 2016	24	CW	Congruent	Mixed	No	MNI	Supplemental Results, Stroop Incongruent > Congruent	C	M	ND	CW
Huang et al., 2012	30	Other-Numerical	Congruent	Mixed	No	MNI	Table 3	-	-	-	O
Huang et al., 2017	33	CW	Neutral	Mixed	No	MNI	Table 3	N	M	ND	-
Jarcho et al., 2013	35	Emotional	Congruent	Mixed	No	MNI	Table 2, main effect conflict monitoring	-	-	-	E
Jaspar et al., 2014	45	CW	Neutral	Mixed	No	MNI	Table S1	N	M	ND	-
Kaufmann et al., 2005	14	Other-Numerical	Congruent	Mixed	No	MNI	Table 4, both tasks	-	-	-	O
Kerns et al., 2005	13	CW	Congruent	Mixed	No	TAL	Table 1, conflict related activity	C	M	ND	CW
Kim et al., 2011	13	CW	Neutral	Mixed	Yes	MNI	Table 1, WResp	-	-	D	-
Kim et al., 2014	18	CW	Congruent	Mixed	Yes	MNI	Table 1	-	-	D	-
Kohn et al., 2020	114	Emotional	Congruent	Mixed	No	MNI	Author sent result table	-	-	-	E
Köhler et al. 2016	45	CW	Congruent	Mixed	Yes	MNI	Supplementar y Data	-	-	D	-
Kozasa et al., 2018	33	CW	Congruent	Blocked	No	MNI	Author sent result image	C	B	ND	CW

Krönke et al., 2018	118	Other-Counting	Congruent	Mixed	No	MNI	Table 7, Incongruent correct > congruent correct	-	-	-	O
Kronhaus et al., 2006	11	CW	Neutral	Blocked	No	TAL	Table 1, controls, stroop	N	B	ND	-
Krug et al., 2012	42	Emotional	Congruent	Mixed	No	MNI	Table 2, I-C, LE task	-	-	-	E
Kühn et al., 2016	19	CW	Congruent	Mixed	No	MNI	Text, fMRI data, p. 2544	C	M	ND	CW
Lesh et al., 2013	54	CW	Congruent	Mixed	No	MNI	Table 2, Stoop I-C contrast control	C	M	ND	CW
Li et al., 2019	20	CW	Congruent	Mixed	No	MNI	Table 1	C	M	ND	CW
Löffler et al., 2019	22	Emotional	Congruent	Mixed	No	MNI	Author sent result table	-	-	-	E
Manard et al., 2017	40	CW	Neutral	Mixed	No	MNI	Table S1	N	M	ND	-
Mathis et al. 2009 A	12	CW	Neutral	Blocked	Yes	MNI	Table 2, Young, Incongruent vs neutral	-	-	D	-
B	12	CW	Congruent, Neutral	Blocked	Yes	MNI	Table 2, middle-aged, Incongruent vs neutral and Incongruent vs. Congruent	-	-	D	-
C	12	CW	Neutral	Blocked	Yes	MNI	Table 2, elderly, Incongruent vs neutral	-	-	D	-
Matthews et al., 2004	18	Other-Counting	Congruent	Blocked	No	MNI	Table 2, congruency effect	-	-	-	O

Mead et al., 2002 A	18	CW	Congruent	Blocked	No	TAL	Table 1, Incongruent > Congruent	C	B*	ND*	CW
B	18	CW	Neutral	Blocked	No	TAL	Table 1, Incongruent > Neutral	N	B*	ND*	-
Milham et al. 2001	16	CW	Neutral	Mixed	No	TAL	Table 1	N	M	ND	-
Mitchell, 2005	13	CW	Neutral	Blocked	No	TAL	Table 3, Interference contrast, Colour modality	N	B	ND	-
Morgenroth et al., 2019	39	CW	Congruent	Mixed	No	MNI	Table s2	C	M	ND	CW
Nakao et al., 2005	14	CW	Congruent	Blocked	No	TAL	Table 4	C	B	ND	CW
Ning et al., 2021	19	CW	Congruent	Mixed	No	MNI	Table S2; sChI-sChC	C	M	ND	CW
Norris et al., 2002	7	CW	Neutral	Blocked	Yes	TAL	Table 1, SE-EPI, coordinates with negative z excluded	-	-	D	-
Ovaysikia et al., 2011	10	Emotional	Congruent	Mixed	No	TAL	Table 1	-	-	-	E
Overbeek et al. 2019	21	CW	Congruent	Mixed	No	MNI	Table S1, Stroop effect	C	M	ND	CW
Papalini et al., 2019 A	58	CW	Congruent	Mixed	No	MNI	Author sent result image	C	M	ND	CW
B	58	Emotional	Congruent	Mixed	No	MNI	Author sent result image	-	-	-	E
Pardo et al., 1990	8	CW	Congruent	Blocked	No	TAL	Table 1, x coordinate flipped	C	B	ND	CW
Park et al., 2008	14	Emotional	Congruent	Mixed	No	MNI	Table 1, normal	-	-	-	E

							controls, relative activation				
Peven et al., 2019<br>A	50	CW	Congruent	Mixed	No	MNI	Author sent result table	C	M*	ND*	CW
B	50	CW	Neutral	Mixed	No	MNI	Author sent result table	N	M*	ND*	-
Piai et al., 2013<br>A	23	CW	Congruent	Mixed	No	MNI	Table 6, whole brain analysis, Incongruent versus congruent	C	M*	ND*	CW
B	23	CW	Neutral	Mixed	No	MNI	Table 6, whole brain analysis, Incongruent versus neutral	N	M*	ND*	-
Pinel et al., 2004	15	Other-Numerical	Congruent	Mixed	No	MNI	Table 2, number/size interference (numerical task), number/size interference (size task)	-	-	-	O
Polk et al., 2008	14	CW	Neutral	Blocked	No	TAL	Table 1	N	B	ND	-
Pompei et al., 2011	48	CW	Neutral	Blocked	No	TAL	Supplemental Material - Task Related Activation during the SCWT, Healthy controls	N	B	ND	-
Portes et al., 2019<br>A	21	CW	Congruent	Blocked	No	MNI	Table 3	C	B	ND	CW
B	19	CW	Congruent	Blocked	No	MNI	Table 4	C	B	ND	CW

Potenza et al., 2003	11	CW	Congruent	Mixed	No	TAL	Table 1, comparison subjects, increased activity, x coordinate flipped	C	M	ND	CW
Prakash et al., 2009											
A	25	CW	Neutral	Mixed	No	MNI	Table 2 A	N	M	ND	-
B	25	CW	Neutral	Mixed	No	MNI	Table 2 B	N	M	ND	-
Puente et al., 2014	26	CW	Congruent	Mixed	No	MNI	Table 6, Normal	C	M	ND	CW
Purmann & Pollmann, 2015	18	CW	Congruent	Mixed	No	MNI	Table 2	C	M	ND	CW
Ramm et al., 2021	19	Other-picture	Congruent	Mixed	No	MNI	Table 1	-	-	-	O
Ravnkilde et al., 2002	46	CW	Congruent	Blocked	No	TAL	Table 1	C	B	ND	CW
Roberts & Hall 2008	16	CW	Neutral	Blocked	No	MNI	Table 3, visual stroop	N	B	ND	-
Robertson et al., 2015	16	Other-Numerical	Congruent	Mixed	No	MNI	Table 3, incongruent >congruent	-	-	-	O
Roth et al., 2006	11	Other-Counting	Congruent	Blocked	No	TAL	Table 2, healthy comparison	-	-	-	O
Ruff et al. 2001	12	CW	Neutral	Mixed	No	MNI	Table 2, Repeated color naming	N	M	ND	-
Salgado-Pineda et al., 2002	11	CW	Congruent	Blocked	No	MNI	Tabla II	C	B	ND	CW
Salgado-Pineda et al., 2021	61	Other-Counting	Congruent	Mixed	No	MNI	Table S1, healthy controls	-	-	-	O
Schmidt et al. 2012							Table S3				
A	31	CW	Congruent	Mixed	No	MNI		C	M*	ND*	CW

B	31	CW	Neutral	Mixed	No	MNI	Author sent result image	N	M*	ND*	-
Schulte et al., 2009	24	CW	Congruent	Blocked	Yes	MNI	Table 1, INC>CON	-	-	D	-
Schulte et al., 2012	17	CW	Congruent	Blocked	Yes	MNI	Table S1, CTL, INC>CON	-	-	D	-
Soek Jeong et al., 2005	10	CW	Congruent	Mixed	Yes	MNI	Table 1, main effect of stroop, healthy controls	-	-	D	-
Shashidhara et al., (2020)	18	CW	Congruent	Blocked	Yes	MNI	Author sent result image	-	-	D	-
Sheu et al., 2012	138	CW	Congruent	Blocked	Yes	MNI	Table S1 (e)	-	-	D	-
Shin & Kim, 2010	43	CW	Congruent	Mixed	Yes	MNI	Table 1	-	-	D	-
Silton et al. 2010	30	CW	Congruent	NA	No	MNI	Table 1	C	-	ND	CW
Song et al. 2015	20	CW	Neutral	Blocked	No	MNI	Table 1	N	B	ND	-
Steel et al., 2001	7	CW	Neutral	Blocked	No	TAL	Table 1	N	B	ND	-
Taylor et al., 1997											
A	12	CW	Neutral	Blocked	No	TAL	Table 1, activations, exclusion of coordinates in parentheses, x coordinate flipped	N	B	ND	-
B	6	CW	Neutral	NA	No	TAL	Table 2, stroop minus symbols, x coordinate flipped	N	-	ND	-
Terry et al., 2012	20	CW	Congruent	Blocked	No	MNI	Table 2, Control group,	C	B	ND	CW

							Stroop Incongruent > Congruent				
Van de Meerendonk et al. 2013	24	CW	Neutral	Mixed	No	MNI	Table 2, Stroop eligible incongruent > eligible neutral	N	M	ND	-
Van't Ent et al., 2014	45	CW	Congruent	Blocked	No	MNI	Table 2, without IBI regressors	C	B	ND	CW
Verdolini et al., 2023	48	Other-Counting	Congruent	Blocked	No	MNI	Table 2	-	-	-	O
Veroude et al., 2013	74	CW	Neutral	Mixed	No	MNI	Table 2, Cognitive Interference	N	M	ND	-
Verstynen, 2014	28	CW	Neutral	Mixed	No	MNI	Table 1, coordinates with negative t score excluded	N	M	ND	-
Wagner et al., 2015	40	CW	Congruent	Mixed	Yes	MNI	Author sent result image	-	-	D	-
Wallentin et al., 2015	49	CW	Congruent	Mixed	Yes	MNI	Table 1	-	-	D	-
Wang et al., 2023	50	Other-picture	Congruent	Mixed	No	MNI	Table S7, Social incongruent > congruent	-	-	-	O
Ye et al. 2009 A	19	CW	Congruent	Mixed	No	MNI	Table 3, Stroop	C	M*	ND*	CW
B	19	CW	Neutral	Mixed	No	MNI	Author sent result image	N	M*	ND*	-
Zhu et al. 2013	26	CW	Congruent	Mixed	No	MNI	Table 1, Stroop Incon vs. Con	C	M	ND	CW
Zoccatelli et al. 2010	10	CW	Congruent	Blocked	No	TAL	Table 1	C	B	ND	CW

Zysset et al., 2001	9	CW	Neutral/ Congruent	Blocked	Yes	TAL	Table 1, Incongruent vs neutral and Incongruent vs congruent	-	-	D	-
Zysset et al., 2007	47	CW	Neutral	Blocked	Yes	TAL	Table 1, coordinates with a negative z value excluded	-	-	D	-
Total 115 studies with 133 experiments								34 Neutral 42 Congruent	26 blocked 36 mixed	20 demand 66 no demand	20 Other 17 Emotional, 42 CW

Note. Meta control: inclusion of the experiment in the meta-analyses I>C (C) or I>N (N); Meta design: inclusion in the meta-analyses across designs of blocking (B) or mixing (M) conditions; Meta demand: inclusion in the meta-analysis with (D) or without (ND) additional cognitive demand; Meta type: inclusion in the meta-analysis across color-word (CW), emotional (E) or other types (O) of Stroop. Letters A, B and C in the study column denote different experiments reported for the same study *Coordinates of contrasts of A and B of the same study merged as one experiment

Table S2

Description and effect sizes of the experiments included in the meta-analyses of the behavioral Stroop effect.

Study	Study ID	N	Stimulus Material	Control Condition	Design	Additional Demand	Source of behavioral data	Hedges g	SE g	r
Agostini et al. (2017)	1	17	CW	congruent	blocked	yes	Table 2, controls	0,27	0,10	
Almdahl et al. (2021)	2	60	emotional	congruent	mixed	no	Data sent by author	0,33	0,03	0,97
Ansari et al. (2006)	3	14	Other - Numerical	congruent	mixed	no	Table 1, small distance	0,20	0,11	
Ansari et al. (2006)	3	14	Other - Numerical	congruent	mixed	no	Table 1, large distance	0,22	0,11	
Bang et al. (2016)	4	43	emotional	congruent	mixed	no	Text, 3.2. behavioral results	0,63	0,07	

Basten et al. (2011)	5	46	CW	congruent	mixed	yes	Table 1, whole sample	0,75	0,09	0,84
Bayer et al. (2018)	6	15	emotional	congruent	mixed	no	Data sent by author	0,51	0,04	0,99
Bayer et al. (2018)	6	15	emotional	congruent	mixed	no	Data sent by author	0,13	0,03	0,99
Becker et al. (2008)	7	17	CW	congruent	mixed	no	Text, p. 2621, behavioral performance	0,54	0,11	
Bench et al. (1993)	8	6	CW	neutral	blocked	no	Table 1, Experiment 1, stroop, crosses	0,77	0,17	
Bench et al. (1993)	8	6	CW	neutral	blocked	no	Table 1, Experiment 1, stroop, neutral	1,03	0,19	
Bench et al. (1993)	8	6	CW	congruent	blocked	no	Table 1, Experiment 2, stroop, crosses	2,95	0,39	
Bench et al. (1993)	8	6	CW	neutral	blocked	no	Table 1, Experiment 2, stroop, congruent	1,53	0,24	
Bohle et al. (2016)	9	20	Other-picture	congruent	mixed	no	Table in Figure 6.7, experiment related and same conditions	0,46	0,1	
Carter et al. (1995)	10	9	CW	congruent	NA	no	Text p. 267, Stroop task performance	0,85	0,15	
Carter et al. (1995)	10	9	CW	neutral	NA	no	Text p. 267, Stroop task performance	0,38	0,13	
Chechko et al. (2009)	11	18	emotional	congruent	mixed	no	Figure 2A, previous trial congruent	0,82	0,11	
Chechko et al. (2009)	11	18	emotional	congruent	mixed	no	Figure 2A, previous trial incongruent	0,17	0,10	
Chechko et al. (2012)	12	24	Other-picture	congruent	mixed	no	Text, p. e38155	0,48	0,09	

Chechko et al. (2012)	12	24	emotional	congruent	mixed	no	Table 2, fearful	0,41	0,09	
Chechko et al. (2012)	12	24	emotional	congruent	mixed	no	Table 2, happy	0,73	0,09	
Chechko et al. (2012)	12	24	emotional	congruent	mixed	no	Table 2, sad	0,62	0,09	
Chechko et al. (2013)	12	18	emotional	congruent	mixed	no	Text, p. 139, data from controls	0,81	0,23	0,63
Chechko et al. (2013)	12	18	Other-picture	congruent	mixed	no	Text, p. 139, data from controls	0,34	0,12	0,87
Chen et al. (2023)	13	30	emotional	congruent	Blocked	no	Text, p.6	0,16	0,08	
Coderre & van Heuven (2013)	14	14	CW	congruent	mixed	no	Figure 2a and 2c (for r calculation), 0 SOA	0,38	0,14	0,85
Coderre & van Heuven (2013)	14	14	CW	neutral	mixed	no	Figure 2a and 2c (for r calculation), 0 SOA	0,38	0,14	0,85
Fan et al. (2017)	15	12	Other-counting	congruent	mixed	no	Table IV, healthy control	0,23	0,12	
Fechir et al. (2010)	16	16	CW	congruent	blocked	no	Table 1, Response time, 30 wpm, author sent correlation between conditions	0,74	0,16	0,83
Fechir et al. (2010)	16	16	CW	congruent	blocked	no	Table 1, Response time, 50 wpm, author sent correlation between conditions	0,67	0,15	0,83
Fedeli et al. (2022)	17	42	Other-counting	Congruent	Mixed	no	Text p.6, numerical stroop	0,28	0,02	0,99
Fleury et al. (2014)	18	11	emotional	congruent	mixed	no	Figure 3, negative faces	0,30	0,13	0,9
Fleury et al. (2014)	18	11	emotional	congruent	mixed	no	Figure 3, positive faces	0,48	0,13	0,9
Godinez et al. (2016)	19	14	emotional	congruent	NA	no	Table 2, RT data of control co-twins	0,36	0,10	0,92

Grandjean et al. (2012)	20	25	CW	congruent	mixed	no	Figure 2, MI context, Author sent correlation between conditions	0,77	0,11	0,88
Grandjean et al. (2012)	20	25	CW	neutral	mixed	no	Figure 2, MI context, Author sent correlation between conditions	0,79	0,11	0,89
Grandjean et al. (2012)	20	25	CW	congruent	mixed	no	Figure 2, MC context, Author sent correlation between conditions	1,13	0,13	0,86
Grandjean et al. (2012)	20	25	CW	neutral	mixed	no	Figure 2, MC context, Author sent correlation between conditions	0,92	0,13	0,84
Grandjean et al. (2012)	20	25	CW	congruent	mixed	no	Figure 2, MN context, Author sent correlation between conditions	1,34	0,13	0,89
Grandjean et al. (2012)	20	25	CW	neutral	mixed	no	Figure 2, MN context, Author sent correlation between conditions	1,21	0,14	0,85
Hart et al. (2010)	21	14	Other- Counting	congruent	mixed	no	Table 1, Reaction time average	0,94	0,13	

Hinault et al. (2019)	22	22	Other- Numerical	congruent	mixed	no	Figure 1, previous trial congruent	0,51	0,09	
Hinault et al. (2019)	22	22	Other- Numerical	congruent	mixed	no	Figure 1, previous trial incongruent	0,21	0,09	
Hoogeveen et al. (2020)	23	193	Other- face	congruent	mixed	no	Downloaded from OSF	0,31	0,03	0,94
Hough et al. (2016)	24	16	CW	congruent	mixed	no	Table 3, Stroop response time, healthy controls, correlation coefficient between conditions sent by author	0,77	0,13	0,89
Huang et al. (2012)	25	15	Other- Numerical	congruent	mixed	no	Table 2, RTs, young, physical size task	0,85	0,12	
Huang et al. (2012)	25	15	Other- Numerical	congruent	mixed	no	Table 2, RTs, young, numerical magnitude task	1,20	0,14	
Huang et al. (2012)	25	15	Other- Numerical	congruent	mixed	no	Table 2, RTs, older, physical size task	0,82	0,12	
Huang et al. (2012)	25	15	Other- Numerical	congruent	mixed	no	Table 2, RTs, older, numerical magnitude task	1,39	0,15	
Huang et al. (2017)	26	33	Other- Numerical	neutral	mixed	no	Table 1, Response time, Incongruent and neutral trials in I blocks	0,53	0,06	0,94
Jarcho et al. (2013)	27	35	emotional	congruent	mixed	no	Table 1, Response time, Conflict Detection	0,40	0,07	0,91
Jaspar et al. (2014)	20	45	CW	congruent	mixed	no	Fig 2A, MI context, correlation between	0,88	0,07	0,93

							conditions sent by author			
Jaspar et al. (2014)	20	45	CW	neutral	mixed	no	Fig. 2A, MI context, correlation between conditions sent by author	0,78	0,06	0,93
Jaspar et al. (2014)	20	45	CW	congruent	mixed	no	Fig. 2A, MC context, correlation between conditions sent by author	1,01	0,08	0,9
Jaspar et al. (2014)	20	45	CW	neutral	mixed	no	Fig 2A, MC context, correlation between conditions sent by author	0,68	0,07	0,91
Jaspar et al. (2014)	20	45	CW	congruent	mixed	no	Fig 2A, MN context, correlation between conditions sent by author	1,17	0,09	0,89
Jaspar et al. (2014)	20	45	CW	neutral	mixed	no	Fig. 2A, MN context, correlation between conditions sent by author	0,96	0,08	0,89
Kaufmann et al. (2005)	28	14	Other- Numerical	congruent	mixed	no	Table 1, numerical comparison Distance 1	0,80	0,12	
Kaufmann et al. (2005)	28	14	Other- Numerical	congruent	mixed	no	Table 1, physical comparison, Distance 1	1,14	0,14	

Kaufmann et al. (2005)	28	14	Other- Numerical	congruent	mixed	no	Table 1, numerical comparison, distance 4	0,46	0,11	
Kaufmann et al. (2005)	28	14	Other- Numerical	congruent	mixed	no	Table 1, physical comparison, distance 4	0,18	0,11	
Kerns et al., 2005	29	13	CW	congruent	mixed	no	text, results section p 1835, healthy comparisons	0,46	0,12	
Kim et al. (2011)	30	13	CW	neutral	mixed	yes	Fig. 3, WR	0,54	0,12	
Kim et al. (2014)	31	18	CW	congruent	mixed	yes	Text, behavioral results, p. 79	1,87	0,16	
Kohn & Fernandez (2020)	32	119	emotional	congruent	mixed	no	Data sent by authors	0,29	0,02	0,98
Kozasa et al. (2018)	33	18	CW	congruent	blocked	no	Table 2, Time reaction, pre, meditators	0,70	0,11	
Kozasa et al. (2018)	33	13	CW	congruent	blocked	no	Table 2, time reaction, Pre, non-meditators	0,80	0,13	
Kozasa et al. (2018)	33	18	CW	neutral	blocked	no	Table 2, time reaction, meditators	0,55	0,10	
Kozasa et al. (2018)	33	13	CW	neutral	blocked	no	Table 2, time reaction, non-meditators	0,62	0,12	
Krönke et al. (2018)	34	118	Other- Counting	congruent	mixed	no	Table 1, RT	0,85	0,04	0,93
Kronhaus et al. (2006)	35	6	CW	neutral	blocked	no	Text, results, p. 32	0,98	0,19	
Krug et al. (2012)	36	42	emotional	congruent	mixed	no	Table 1, RT, high expectancy	0,20	0,03	0,98
Krug et al. (2012)	36	42	emotional	congruent	mixed	no	Table 1, RT, low expectancy	0,56	0,05	0,95
Lesh et al. (2013)	37	54	CW	congruent	mixed	no	Table 1, stroop reaction time, healthy controls	0,72	0,06	

Loeffler et al. (2019)	38	24	emotional	congruent	mixed	no	Data sent by author	0,41	0,07	0,94
Manard et al. (2017)	20	20	CW	congruent	mixed	no	Table 2, response times, young, MI, correlation between conditions sent by author	0,43	0,12	0,87
Manard et al. (2017)	20	20	CW	neutral	mixed	no	Table 2, response times, young, MI, correlation between conditions sent by author	0,56	0,10	0,91
Manard et al. (2017)	20	20	CW	congruent	mixed	no	Table 2, response times, young, MC, correlation between conditions sent by author	0,88	0,16	0,8
Manard et al. (2017)	20	20	CW	neutral	mixed	no	Table 2, response times, young, MC, correlation between conditions sent by author	0,68	0,17	0,76
Manard et al. (2017)	20	20	CW	congruent	mixed	no	Table 2, response times, young, MN, correlation between conditions sent by author	0,92	0,15	0,83
Manard et al. (2017)	20	20	CW	neutral	mixed	no	Table 2, response times, young, MN, correlation between conditions sent by author	0,82	0,13	0,87

Manard et al. (2017)	20	20	CW	congruent	mixed	no	Table 2, response times, older, MI, correlation between conditions sent by author	0,66	0,08	0,94
Manard et al. (2017)	20	20	CW	neutral	mixed	no	Table 2, response times, older, MI, correlation between conditions sent by author	0,71	0,09	0,92
Manard et al. (2017)	20	20	CW	congruent	mixed	no	Table 2, response times, older, MC, correlation between conditions sent by author	0,85	0,09	0,94
Manard et al. (2017)	20	20	CW	neutral	mixed	no	Table 2, response times, older, MC, correlation between conditions sent by author	0,55	0,09	0,92
Manard et al. (2017)	20	20	CW	congruent	mixed	no	Table 2, response times, older, MN, correlation between conditions sent by author	1,08	0,11	0,92
Manard et al. (2017)	20	20	CW	neutral	mixed	no	Table 2, response times, older, MN, correlation between conditions sent by author	0,83	0,09	0,93
Mathis et al. (2009)	39	12	CW	congruent	blocked	yes	Table 1, RT, young	0,54	0,12	

Mathis et al. (2009)	39	12	CW	neutral	blocked	yes	Table 1, RT, young	0,62	0,13	
Mathis et al. (2009)	39	12	CW	congruent	blocked	yes	Table 1, RT, middle-aged	0,51	0,12	
Mathis et al. (2009)	39	12	CW	neutral	blocked	yes	Table 1, RT, middle-aged	0,72	0,13	
Mathis et al. (2009)	39	12	CW	congruent	blocked	yes	Table 1, RT, elderly	1,25	0,16	
Mathis et al. (2009)	39	12	CW	neutral	blocked	yes	Table 1, RT, elderly	1,00	0,14	
Matthews et al. (2004)	40	18	Other- Counting	congruent	blocked	no	Table 1, 1,5 s trials	2,27	0,19	
Matthews et al. (2004)	40	18	Other- Counting	congruent	blocked	no	Table 1, 2 s trials	2,05	0,17	
Mead et al. (2002)	41	18	CW	congruent	blocked	no	Text, behavioral findings, p. 738	0,76	0,11	
Mead et al. (2002)	41	18	CW	neutral	blocked	no	Text, behavioral findings, p. 738	0,43	0,10	
Mitchell et al. (2005)	42	13	CW	congruent	blocked	no	Table 2, reaction time, Colour modality	0,30	0,11	
Mitchell et al. (2005)	42	13	CW	neutral	blocked	no	Table 2, reaction time, Colour modality	0,14	0,11	
Mitchell et al. (2005)	42	13	Other-Counting	congruent	blocked	no	Table 2, reaction time, Number modality	1,68	0,18	
Morgenroth et al. (2019)	43	39	CW	congruent	mixed	no	Individual response logfiles from downloaded from OSF	0,68	0,07	0,91
Ovaysikia et al. (2011)	44	10	emotional	congruent	mixed	no	Figure 4C	0,50	0,26	0,66
Overbeek et al. (2019)	45	21	CW	congruent	mixed	no	Table 1; HCs, Reaction time	0,62	0,12	0,86
Papaline et al. (2018)	32	29	CW	congruent	mixed	no	Table S3, color-word stroop, placebo pre	0,49	0,08	

Papalini et al. (2019)	32	29	CW	congruent	mixed	no	Table S3, color-word stroop, probiotics pre	0,40	0,08	
Papalini et al. (2019)	32	29	emotional	congruent	mixed	no	Table S3, emotional face-word stroop, placebo pre	0,31	0,08	
Papalini et al. (2019)	32	29	emotional	congruent	mixed	no	Table S3, emotional face-word stroop, probiotics pre	0,27	0,08	
Park et al. (2008)	46	14	emotional	congruent	mixed	no	Text, p. 121, median and interquartile range for control group, mean and SD estimate from median and IQR (Wan et al., 2014)	0,54	0,12	
Peven et al. (2019)	47	50	CW	congruent	mixed	no	Text, p.61	1,15	0,15	0,67
Peven et al. (2019)	47	50	CW	neutral	mixed	no	Text, p.61	0,80	0,12	0,74
Piai et al. (2013)	48	17	CW	congruent	mixed	no	Table 1, Table 4 Stroop, data to calculate correlation sent by author	0,54	0,07	0,96
Piai et al. (2013)	48	17	CW	neutral	mixed	no	Table 1, Table 4 Stroop, data to calculate correlation sent by author	0,32	0,05	0,98
Pompei et al. (2011)	49	48	CW	neutral	blocked	no	Table 1, Behavioural performance in the Stroop Colour Word Test, Reaction time, controls	0,50	0,06	

Portes et al. (2019)	33	19	CW	congruent	blocked	no	Raw data sent by author	0,50	0,10	0,91
Portes et al. (2019)	33	21	CW	congruent	blocked	no	Raw data sent by author	0,83	0,17	0,77
Portes et al. (2019)	33	19	CW	neutral	blocked	no	Raw data sent by author	0,37	0,09	0,93
Portes et al. (2019)	33	21	CW	neutral	blocked	no	Raw data sent by author	0,61	0,15	0,78
Prakash et al. (2009)	50	25	CW	congruent	mixed	no	Table 1, Reaction time old	0,78	0,09	
Prakash et al. (2009)	50	25	CW	neutral	mixed	no	Table 1, Reaction time old	0,81	0,10	
Prakash et al. (2009)	50	25	CW	congruent	mixed	no	Table 1, Reaction time young	0,59	0,09	
Prakash et al. (2009)	50	25	CW	neutral	mixed	no	Table 1, Reaction time young	0,46	0,09	
Puente et al. (2014)	51	43	CW	congruent	mixed	no	Table 3	1,24	0,09	
Puente et al. (2014)	51	43	CW	neutral	mixed	no	Table 3	0,98	0,08	
Ramm et al. (2021)	52	19	Other-picture	congruent	mixed	no	Data sent by author	0,64	0,13	0,85
Robertson et al. (2015)	53	16	Other- Numerical	congruent	mixed	no	Table 1	0,68	0,09	0,94
Roth et al. (2006)	54	10	Other- Counting	congruent	blocked	no	Table 1, Healthy adult	0,37	0,22	0,74
Ruff et al. (2001)	55	12	CW	neutral	mixed	no	Table 1, Repeated response, Reaction time	1,07	0,14	0,91
Schmidt et al. (2012)	20	16	CW	congruent	mixed	no	Table 1, Reaction time, morning session, morning type, correlation sent by author	1,21	0,25	0,69
Schmidt et al. (2012)	20	15	CW	congruent	mixed	no	Table 1, Reaction time, morning session, evening type, correlation sent by author	0,46	0,06	0,97

Schmidt et al. (2012)	20	16	CW	congruent	mixed	no	Table 1, Reaction time, evening session, morning type, correlation sent by author	1,30	0,28	0,64
Schmidt et al. (2012)	20	15	CW	congruent	mixed	no	Table 1, Reaction time, evening session, evening type, correlation sent by author	0,51	0,10	0,92
Schmidt et al. (2012)	20	16	CW	neutral	mixed	no	Table 1, Reaction time, morning session, morning type, correlation sent by author	1,45	0,32	0,59
Schmidt et al. (2012)	20	15	CW	neutral	mixed	no	Table 1, Reaction time, morning session, evening type, correlation sent by author	0,65	0,12	0,91
Schmidt et al. (2012)	20	16	CW	neutral	mixed	no	Table 1, Reaction time, evening session, morning type, correlation sent by author	1,24	0,22	0,77
Schmidt et al. (2012)	20	15	CW	neutral	mixed	no	Table 1, Reaction time, evening session, evening type, correlation sent by author	0,38	0,06	0,97
Schulte et al. (2009)	56	24	CW	congruent	blocked	yes	Data sent by author	0,25	0,09	
Schulte et al. (2009)	56	24	CW	congruent	blocked	yes	Data sent by author	0,37	0,09	
Schulte et al. (2009)	56	24	CW	congruent	blocked	yes	Data sent by author	0,12	0,08	
Schulte et al. (2009)	56	24	CW	congruent	blocked	yes	Data sent by author	0,15	0,08	

Schulte et al. (2012)	56	17	CW	congruent	blocked	yes	Figure 2 CTL, match, response repetition	0,47	0,10	
Schulte et al. (2012)	56	17	CW	congruent	blocked	yes	Figure 2, CTL, nonmatch, response repetition	-0,06	0,10	
Schulte et al. (2012)	56	17	CW	congruent	blocked	yes	Figure 2 CTL, match, response switching	0,33	0,10	
Schulte et al. (2012)	56	17	CW	congruent	blocked	yes	Figure 2, CTL, nonmatch, response switching	0,31	0,10	
Shashidhara et al. (2020)	57	18	CW	congruent	blocked	yes	Table 1, Stroop, RT	1,35	0,19	0,82
Shin & Kim (2015)	31	43	CW	congruent	mixed	yes	Text, behavioral results, RT on current trial, p.80	0,92	0,08	
Song et al. (2015)	58	20	CW	neutral	blocked	no	Fig 3 B, Test 1 and Test 2	1,21	0,12	
Taylor et al. (1997)	59	12	CW	neutral	blocked	no	Text, results task performance, RT, p. 85	2,00	0,36	0,73
Terry et al. (2012)	60	20	CW	congruent	blocked	no	Table 1, Controls, Stroop task, RT	0,45	0,10	0,9
van't Ent et al. (2013)	61	46	CW	congruent	blocked	no	Table 1, Stroop, Reaction time, Test sample	0,91	0,13	0,72
van't Ent et al. (2013)	61	45	CW	congruent	blocked	no	Table 1, Stroop, Reaction time, Repetition sample	1,17	0,16	0,67
Veroude et al. (2013)	62	74	CW	congruent	mixed	no	Table 1, Reaction times for the non-emotional conditions	0,74	0,06	
Veroude et al. (2013)	62	74	CW	neutral	mixed	no	Table 1, Reaction times for the non-	0,61	0,05	0,91

							emotional conditions			
Verstynen (2014)	63	28	CW	congruent	mixed	no	Figure 1A	1,18	0,10	
Verstynen (2014)	63	28	CW	neutral	mixed	no	Figure 1A	0,78	0,09	0,91
Wallentin et al. (2015)	64	49	CW	congruent	mixed	yes	Figure 2A, low frequency VS and VNS	0,37	0,06	
Wallentin et al. (2015)	64	49	CW	congruent	mixed	yes	Figure 2A, high frequency VS and VNS	0,50	0,06	
Wang et al. (2023)	65	50	Other-picture	congruent	mixed	no	Table S3, social	0,33	0,06	
Ye & Zhou (2009)	66	19	CW	congruent	mixed	no	Text, behavioral results, RT Stroop p. 284, 285	0,47	0,10	
Ye & Zhou (2009)	66	19	CW	neutral	mixed	no	Text, behavioral results, RT Stroop p. 284, 285	0,31	0,10	
Zoccatelli et al. (2010)	67	10	CW	congruent	blocked	no	Figure 2, color-word	1,44	0,18	
Zysset et al. (2001)	68	9	CW	congruent	blocked	yes	Text, Behavioral results, p.32, RT	0,54	0,14	
Zysset et al. (2001)	68	9	CW	neutral	blocked	yes	Text, Behavioral results, p.32, RT	0,67	0,14	
Zysset et al. (2007)	68	24	CW	neutral	blocked	yes	Data sent by author	0,53	0,08	0,93
Zysset et al. (2007)	68	23	CW	neutral	blocked	yes	Data sent by author	0,48	0,07	0,94

Note. Hedges g: effect size of the difference in reaction time between incongruent and congruent or neutral condition, SEg: standard error of the effect size, r: correlation between conditions (incongruent – congruent or incongruent-neutral).

Table S3

Checklist for neuroimaging meta-analysis

The research question is specifically defined	YES, commonalities and differences between different forms of the Stroop task, with 8 different meta-analyses<br>1) Color-Word Stroop (=Congruent Control)<br>2) Emotional Stroop<br>3) Other types of Stroop<br>4) Neutral Control<br>5) Blocked Design<br>6) Mixed Design<br>7) With additional cognitive demands<br>8) Without additional cognitive demands
The literature seach was systematic	YES, it included the following keywords in the following databases:<br><br>1) "fMRI", "PET", "neuroimaging", "Stroop", "Inhibition"; "interference".<br>2) Databases: PubMed (https://www.ncbi.nlm.nih.gov/pubmed/), Google Scholar (http://scholar.google.de), web-of-knowledge (https://apps.webofknowledge.com) for journal publications; SCOPUS, NDLDT and OADT for theses, dissertations and conference posters/presentations<br><br>Authors were additionally contacted to provide additional information
Detailed inclusion and exclusion criteria are included	YES, inclusion of:<br><br>- fMRI and PET studies, which reported the coordinates in a standard reference space (Montreal Neurological Institute (MNI) or Talairach-Tournoux system (TAL))<br>- Healthy participants over the age of 18 and without any pharmacological manipulations<br>- Activation data<br>- Whole-brain data<br>- No correlation or interaction with other variables (e.g., performance measures)

	- Only tasks that induce response and semantic conflict<br>- No re-analyzation of data of the same subjects<br>- Task using visual stimuli<br>- Contrast of incongruent against congruent or neutral condition<br>- 2-dimensional stimuli with overlap in stimulus dimensions activating different processes<br>- At least one semantic stimulus dimension
Sample overlap was taken into account	YES, using the following method:<br><br>- If a study reported several experiments eligible for inclusion, the reported coordinates were pooled to constitute a single experiment<br>- Exclusion of studies that re-analyzed data of the same subjects of a previous study<br>- If a study separately reported two or more subject groups, e.g., young and old participants with separate results, the coordinates were not pooled
All experiments use the same search coverage (state how brain coverage is assessed and how small volume corrections and conjunctions are taken into account)	YES, the search coverage is the following:<br><br>- Only whole-brain coverage<br>- Exclusion of ROI studies<br>- Exclusion of partial brain coverage
Studies are converted to a common reference space	YES, using the following conversion(s):<br><br>- Coordinates reported in Talairach space were converted to MNI space (Lancaster et al., 2007).
Data extraction have been conducted by two investigators (ideal case) or double checked by the same investigator (state how double- checking was performed)	YES:<br><br>- Veronika Müller, Linda Ficco, Sandra Tyralla, Taraneh Aziz-Safaie checked inclusion criteria<br>- Veronika Müller and Linda Ficco checked existing database of Cieslik et al., (2015) and Chen et al., (2018)<br>- Linda Ficco, Sandra Tyralla and Taraneh Aziz-Safaie conducted literature research

	- Linda Ficco, Sandra Tyralla and Taraneh Aziz-Safaie extracted coordinates from studies of the literature research<br>- Linda Ficco, Sandra Tyralla, Taraneh Aziz-Safaie extracted other info: Number of subjects included, task type, control condition, space<br>- Veronika Müller double-checked the following data: Coordinates extracted, number of subjects included, task type, control condition, space<br>- Veronika Müller extracted other info: mixing or blocking conditions, additional cognitive demand<br>- Veronika Müller updated the literature search in fall 2023, checked inclusion criteria and extracted data<br>- Edna Cieslik double-checked the newly extracted data
The paper includes a table with at least the references, basic study description (e.g. for fMRI tasks, stimuli), contrasts and basic sample descriptions (e.g. size, mean age and gender distribution, specific characteristics) of the included studies, source of information (e.g. contact with authors), reference space	YES, the table includes the following information:<br><br>- First author and year<br>- Number of subjects<br>- Space<br>- Control Condition<br>- Presentation Design<br>- Additional cognitive demand<br>- Stimulus material<br>- Source of coordinates<br>- If further information was received by the authors<br>- Inclusion of experiments in the different meta-analyses
All analyses were planned beforehand, including the methods and parameters used for inference, correction for multiple testing, etc.	YES for neuroimaging meta-analyses; effect size meta-analyses were planned after neuroimaging meta-analyses<br>The neuroimaging meta-analysis used the default methods and parameters of our group as suggested in Eickhoff et al. (2016) and Frahm et al. (2022)
The meta-analysis includes diagnostics	Contributions are provided for each meta-analysis performed

Table S4a

Sensitivity analyses for correlation between conditions

	0	0.1	0.2	0.3	0.4	0.5	0.6	0.7	0.8	0.9	real
Estimate	0.592	0.596	0.599	0.603	0.606	0.609	0.612	0.613	0.612	0.598	0.594
SE	0.061	0.060	0.060	0.059	0.058	0.058	0.058	0.058	0.058	0.057	0.056
Tau	0.052	0.058	0.064	0.070	0.076	0.082	0.087	0.092	0.095	0.093	0.162

Note. Sensitivity analyses varying the correlation between incongruent and congruent/neutral conditions from 0 to 0.9 in steps of 0.1 showing that the aggregated effects size (estimate), SE and Tau varied with different correlations between conditions.

Table S4b

Sensitivity analyses for within-study effect size correlations

	0	0.2	0.4	0.6	0.8	1
Estimate	0.594	0.594	0.594	0.594	0.594	0.594
SE	0.056	0.056	0.056	0.056	0.056	0.056
Tau	0.162	0.162	0.162	0.162	0.162	0.162

Note. Sensitivity analyses varying the within-study effect size correlation (rho) from 0 to 1 in steps of 0.2 revealed that the aggregated effects size (estimate), SE and Tau are not affected by variations of rho.

Table S5
Additional analysis across color-word Stroop experiments modeling in addition to control condition, design and demand also type of neutral control condition. No significant effect was found.

	Estimate	SE	t	df	p	95 % CI
**Intercept**	0.81	0.12	6.5	14.8	<0.0001	0.54 – 1.07
**Control Condition neutral symbols**	0.04	0.12	0.34	14	0.74	-0.21 – 0.29
**Control Condition neutral words**	-0.1	0.12	-0.8	14.6	0.44	-0.37 – 0.17
**Design**	-0.08	0.13	-0.57	25.7	0.57	-0.35 – 0.2
**Demand**	-0.1	0.16	-0.65	14.1	0.53	-0.44 – 0.27

Table S6
Additional analysis across color-word Stroop experiments modeling in addition to control condition, design and demand, the response modality, number of responses, number of colors as well as the interaction between design and control condition. No significant effect was found. Please

note that only 38 studies with 110 effect sizes were part of the analyses as one study did not report the number of responses and colors and thus had to be excluded from this subanalysis.

	Estimate	SE	t	df	p	95 % CI
**Intercept**	0.59	0.62	0.96	7.82	0.37	-0.84 – 2.03
**Control Condition**	-0.07	0.20	-0.36	11.13	0.73	-0.51 – 0.36
**Design**	-0.10	0.17	-0.58	17.23	0.57	-0.46 – 0.26
**Demand**	-0.26	0.25	-1.06	6.63	0.33	-0.86 – 0.33
**Response modality**	0.23	0.31	0.74	5.37	0.49	-0.55 – 1
**Number of Responses**	-0.11	0.14	-0.79	7.27	0.46	-0.43 – 0.21
**Number of Colours**	0.16	0.10	1.56	4.81	0.18	-0.11 – 0.42
**Interaction ControlxDesign**	-0.03	0.23	-0.12	21.43	0.91	-0.50 – 0.45

Table S7

Clusters of significant convergence in the meta-analyses across experiments contrasting against a congruent (Incongruent >Congruent or Neutral (Incongruent>Neutral) control condition, their center of mass coordinate, histological assignments as well as experiments contributing.

Brain Structure	MNI Coordinates	Histological Assignments	Contributions
**Incongruent > Congruent**			
**Cluster 1 (k=1111)**			36 experiments (Kerns et al., 2005; Pardo et al., 1990; Ye et al., 2005; Brass et al., 2005; Becker et al., 2008; Grandjean et al., 2012/2013; Zhu et al., 2013; Fan et al., 2003; Coderre et al., 2008; Silton et al, 2010; Ravnkilde et al., 2002; Nakao et al., 2005; Zoccatelli et al., 2010; Mead et al., 2002; Chen et al., 2018; Fechir et al., 2010; Lesh et al., 2013; Schmidt et al., 2012; Van't Ent et al., 2014; Barkley-Levenson et al., 2018; Kühn et al., 2016; Morgenroth et al., 2019; Overbeek et al., 2019; Portes et al., 2019A; Portes et al., 2019B; Purmann & Pollmann, 2015; Peven et al., 2019; Kozasa et al, 2018; Ghavidel et al., 2020; Papalini et al., 2019; Coderre et al., 2013; Li et al., 2019; Ning et al., 2021; Salgado-Pineda et al., 2002; Puente et al., 2014; Chen, Meng et al., 2023)
L inferior/middle frontal gyrus	-44 14 28	44	

**Cluster 2 (k=1007)**			32 experiments (Kerns et al., 2005; Pardo et al., 1990; Carter et al., 1995; Ye et al., 2005; Brass et al., 2005; Becker et al., 2008; Zhu et al., 2003; Fan et al., 2003; Coderre et al., 2008; Silton et al, 2010; Ravnkilde et al., 2002; Potenza et al., 2003; Nakao et al., 2005; Zoccatelli et al., 2010; Chen et al., 2018; DeVito et al., 2012; Fechir et al., 2010; Lesh et al., 2013; Schmidt et al., 2012; Barkley-Levenson et al., 2018; Kühn et al., 2016; Morgenroth et al., 2019; Overbeek et al., 2019; Portes et al., 2019A; Purmann & Pollmann, 2015; Ghavidel et al., 2020; Papalini et al., 2019; Coderre et al., 2013; Li et al., 2019; Ning et al., 2021; Puente et al., 2014; Chen,Meng et al., 2023)
Posterior medial frontal cortex	0 18 46	6mr	
**Cluster 3 (k=637)**			30 experiments (Carter et al. 1995; Ye et al., 2009; Becker et al., 2008; Grandjean et al., 2012/2013; Zhu et al., 2013; Fan et al., 2003; Coderre et al., 2008; Silton et al, 2010; Potenza et al., 2003; Nakao et al., 2005; Zoccatelli et al., 2010; Chen et al., 2018; DeVito et al., 2012; Fechir et al., 2010; Lesh et al., 2013; Schmidt et al., 2012; Terry et al., 2012; Van't Ent et al., 2014; Barkley-Levenson et al., 2018; Overbeek et al., 2019; Portes et al., 2019A; Purmann & Pollmann, 2015; Kozasa et al, 2018; Papalini et al., 2019; Coderre et al., 2013; Li et al., 2019; Ning et al., 2021, Salgado-Pineda et al., 2002; Puente et al., 2014; Chen, Meng et al., 2023)
L intraparietal sulcus	-32 -56 46	hIP3, hIP1, hIP6	
**Cluster 4 (k=390)**			19 experiments (Ye et al., 2009; Brass et al., 2005; Zhu et al., 2013; Silton et al., 2010; Potenza et al., 2003; Nakao et al., 2005; Zoccatelli et al., 2010; Chen et al., 2018; DeVito et al., 2012; Hough et al.. 2016; Piai et al., 2013; Schmidt et al., 2012; Barkley-Levenson et al., 2018; Kühn et al., 2016; Overbeek et al., 2019; Portes et al., 2019B; Purmann & Pollmann, 2015; Li et al., 2019; Ning et al., 2021)
R anterior insula	38 22 -3	Id7, Id6	
**Cluster 5 (k=266)**			15 experiments (Pardo et al., 1990; Ye et al., 2009; Becker et al., 2008; Silton et al., 2010; Ravnkilde et al., 2002; Potenza et al., 2003; Zoccatelli et al., 2010; DeVito et al., 2012; Hough et al., 2016; Schmidt et al., 2012; Barkley-Levenson et al., 2018; Morgenroth et al., 2019; Purmann & Pollmann, 2015; Li et al., 2019; Ning et al., 2021)
L anterior insula	-34 20 -3	Id7, Id6	
**Cluster 6 (k=218)**			12 experiments (Ye et al., 2009; Brass et al., 2005; Grandjean et al., 2012/2013; Zhu et al, 2013; Silton et al., 2010; Hough et al., 2016; Schmidt et al., 2012; Van't Ent et al., 2014; Peven et al., 2018; Papalini et al., 2019; Ning et al., 2021; Chen, Meng et al., 2023)
L fusiform gyrus	-48 -56 -16	FG4, FG2	
**Cluster 7 (k=107)**			

R inferior/middle frontal gyrus	48 22 26		12 experiments (Kerns et al., 2005; Brass et al., 2005; Coderre et al., 2008; Potenza et al., 2003; Nakao et al., 2003; Zoccatelli et al., 2010; Lesh et al., 2013; Schmidt et al., 2012; Van't Ent et al., 2014; Barkley-Levenson et al., 2018; Overbeek et al., 2019; Purmann & Pollmann, 2015)
**Cluster 8 (k =98)**			7 experiments (Fan et al., 2003; Silton et al., 2010; Fechir et al., 2010, Terry et al., 2012; Purmann & Pollmann, 2015; Papalini et al., 2019; Puente et al., 2014)
Precuneus	-4 -60 45		
**Incongruent > Neutral**			
**Cluster 1 (k=950)**			25 experiments (Bench et al., 1993; Banich et al., 2001; Adleman et al., 2002; Ye et al., 2009; Polk et al., 2008; Grandjean et al., 2012/2013; Mitchell, 2005; Prakash et al., 2009A; Prakash et al., 2009B; Coderre et al., 2008, Taylor et al., 1997A; George et al., 1994; Milham et al., 2001; Ruff et al., 2001; Manard et al., 2017; Pompei et al., 2011; Schmidt et al., 2012; Song et al, 2015; Veroude et al., 2013; Verstynen, 2014; Barkley-Levenson et al., 2018; Jaspar et al., 2014; Peven et al., 2019; Roberts & Hall, 2008; van de Meerendonk et al., 2013)
L intraparietal sulcus/superior parietal lobe	-32 -58 44	hIP3, hIP6, hIP1, hIP2, hIP5	
**Cluster 2 (k=775)**			24 experiments (Banich et al., 2001; Adleman et al., 2002; Ye et al., 2009; Polk et al., 2008; Grandjean et al., 2012/2013; Prakash et al., 2009A; Prakash et al., 2009B; Coderre et al. 2008; Taylor et al., 1997A; Taylor et al., 1997B; Milham et al., 2001; Ruff et al., 2001; Mead et al., 2002; Manard et al., 2017; Pompei et al., 2011; Song et al, 2015; Veroude et al., 2013; Verstynen, 2014; Barkley-Levenson et al., 2018; Jaspar et al., 2014; Peven et al., 2019; Roberts & Hall, 2008; van de Meerendonk et al., 2013; Coderre et al., 2013)
L inferior/middle frontal gyrus	-46 16 30		
**Cluster 3 (k=490)**			17 experiments (Bench et al., 1993; Ye et al., 2009; Polk et al., 2008; Grandjean et al., 2012/2013; Prakash et al., 2009B; Taylor et al., 1997A; George et al., 1994; Steel et al., 2001; Manard et al., 2017; Piai et al., 2013; Pompei et al., 2011; Schmidt et al., 2012, Veorude et al., 2013; Verstynen, 2014; Barkley-Levenson et al., 2018; Jaspar et al., 2014; Peven et al., 2019)
R anterior insula	34 20 -2	Id7, Id6	
**Cluster 4 (k=389)**			16 experiments (Banich et al., 2001; Ye et al., 2009; Polk et al., 2008; Grandjean et al., 2012/2013; Coderre et al., 2008; Milham et al., 2001; Ruff et al., 2001; Piai et al., 2013; Schmidt et al., 2012; Veroude et al., 2013;
Posterior medial frontal cortex	0 16 48	6mr	

			Verstynen, 2014; Barkley-Levenson et al., 2018; Jaspar et al., 2014; Peven et al., 2019; Roberts & Hall, 2008; Coderre et al., 2013)
**Cluster 5 (k=298)**			15 experiments (Banich et al., 2001; Ye et al., 2009; Polk et al., 2008; Grandjean et al., 2012/2013; Mitchell, 2005; Prakash et al., 2009B; Taylor et al., 1997A; George et al., 1994; Steel et al., 2001; Manard et al., 2017; Schmidt et al., 2012; Veroude et al., 2013; Verstynen, 2014; Barkley-Levenson et al., 2018; Jaspar et al., 2014)
L anterior insula	-33 20 0	Id7, Id6	
**Cluster 6 (k=184)**			11 experiments (Ye et al., 2009; Prakash et al., 2009B; Taylor et al., 1997; Milham et al., 2001; Huang et al., 2017; Schmidt et al., 2012; Veroude et al., 2013; Verstynen, 2014; Jaspar et al., 2014; Peven et al., 2019; Roberts & Hall, 2008)
R inferior/middle frontal gyrus	46 12 30		
**Cluster 7 (k=119)**			10 experiments (Bench et al., 1993; Ye et al., 2008; Polk et al., 2008; Grandjean et al., 2012/2013; Coderre et al., 2008; Manard et al., 2017; Pompei et al., 2011; Schmidt et al., 2012; Veroude et al., 2013; Peven et al., 2019)
R intraparietal sulcus	34 -54 48	hIP1, hIP3	

Table S8
Clusters of significant convergence in the meta-analyses across experiments that block or mix incongruent, congruent and/neutral conditions, their center of mass coordinate, histological assignments as well as experiments contributing.

Brain Structure	MNI Coordinates	Histological Assignments	Contributions
**Blocked Design**			
**Cluster 1 (k=667)**			18 experiments (Pardo et al., 1990; Polk et al., 2008; Coderre et al., 2008; Taylor et al., 1997; Ravnkilde et al., 2002; Nakao et al., 2005; Zoccatelli et al., 2010; Mead et al., 2002; Fechir et al., 2010; Pompei et al., 2011; Song et al., 2015; Van’t Ent et al., 2014; Portes et al., 2019A; Portes et al., 2019B; Kozasa et al., 2018; Ghavidel et al., 2020; Robert & Hall, 2008, Salgado-Pineda et al., 2002)
L inferior/middle frontal gyrus	-46 12 28	44	
**Cluster 2 (k=488)**			17 experiments (Bench et al., 1993; Adleman et al., 2002; Polk et al., 2008; Mitchell, 2005; Taylor et al., 1997; George et al., 1994; Nakao et al., 2005; Zoccatelli et al., 2010; Fechir et al., 2010; Pompei et al., 2011; Song et al.,
L intraparietal sulcus/superior parietal lobe	-28 -62 46	hIP3, hIP6, hIP5	

			2015; Terry et al., 2012; Van't Ent et al., 2014; Portes et al., 2019A; Kozasa et al., 2018; Roberts & Hall, 2008; Salgado-Pineda et al., 2002)
**Cluster 3 (k=160)**			7 experiments (Bench et al., 1993; Polk et al., 2008; Taylor et al., 1997; Nakao et al., 2005; Zoccatelli et al., 2010; Pompei et al., 2011; Roberts & Hall, 2008)
R orbitofrontal cortex	40 28 -12		
**Cluster 4 (k=157)**			8 experiments (Pardo et al., 1990; Coderre et al., 2008; Ravnkilde et al., 2002; Nakao et al, 2005; Zoccatellli et al., 2010; Fechir et al., 2010; Ghavidel et al., 2020; Roberts & Hall, 2008)
Posterior medial frontal cortex	-6 26 46		
**Cluster 5 (k=117)**			7 experiments (Pardo et al., 1990; Polk et al., 2008; Taylor et al., 1997; George et al., 1994; Steel et al., 2001; Ravnkilde et al., 2002; Zoccatelli et al., 2010)
L anterior insula	-32 20 -6		
**Mixed Design**			
**Cluster 1 (k=1066)**			30 experiments (Kerns et al., 2005; Ye et al., 2009; Brass et al., 2005; Becker et al., 2008; Grandjean et al., 2012/2013; Prakash et al., 2009A, Prakash et al., 2009B; Fan et al., 2003; Milham et al., 2001; Ruff et al., 2001; Chen et al., 2018; Lesh et al., 2013; Manard et al., 2017; Schmidt et al., 2012; Veroude et al., 2013; Verstynen, 2014; Coderre et al., 2013; Barkley-Levenson et al., 2018; Kühn et al., 2016; Jaspar et al., 2014; Morgenroth et al., 2019; Overbeek et al., 2019; Purmann & Pollmann, 2015; Peven et al., 2019; Papalini et al., 2019; van de Meerendonk et al., 2013; Ning et al., 2021; Li et al., 2019, Puente et al., 2014; Chen, Meng et al., 2023)
L inferior/middle frontal gyrus	-44 14 30	44	
**Cluster 2 (k=938)**			29 experiments (Ye et al., 2009; Becker et al., 2008; Grandjean et al., 2012/2013; Prakash et al., 2009A, Prakash et al., 2009B; Zhu et al., 2013; Fan et al., 2003; Milham et al., 2001; Ruff et al., 2001; Potenza et al., 2003; Chen et al., 2018; DeVito et al., 2012; Lesh et al., 2013; Manard et al., 2017; Schmidt et al., 2012; Veroude et al., 2013; Verstynen, 2014; Coderre et al., 2013; Barkley-Levenson et al., 2018; Jaspar et al., 2014; Overbeek et al., 2019; Purmann & Pollmann, 2015; Peven et al., 2019; Papalini et al., 2019;
L intraparietal sulcus	-32 -56 46	hIP3, hIP1, hIP2, hIP6	

			van de Meerendonk et al., 2013; Ning et al., 2021; Li et al., 2019, Puente et al., 2014; Chen, Meng et al., 2023)
**Cluster 3 (k=763)**			28 experiments (Kerns et al., 2005; Ye et al., 2009; Brass et al., 2005; Becker et al., 2008; Grandjean et al., 2012/2013; Zhu et al., 2013; Milham et al., 2001; Ruff et al., 2001; Potenza et al., 2003; Chen et al., 2018; DeVito et al., 2012; Lesh et al., 2013; Piai et al., 2013; Schmidt et al., 2012; Veroude et al., 2013; Verstynen, 2014; Coderre et al., 2013; Barkley-Levenson et al., 2018; Kühn et al., 2016; Jaspar et al., 2014; Morgenroth et al., 2019; Purmann & Pollmann, 2015; Peven et al., 2019; Papalini et al., 2019; Ning et al., 2021; Li et al., 2019, Puente et al., 2014; Chen, Meng et al., 2023)
Posterior medial frontal cortex	0 14 50	6mr	
**Cluster 4 (k=620)**			22 experiments (Ye et al., 2009; Brass et al., 2005; Grandjean et al., 2012/2013; Prakash et al., 2009B; Zhu et al., 2013; Potenza et al., 2003; Chen et al., 2018; DeVito et al., 2012; Hough et al., 2016; Manard et al., 2017; Piai et al., 2013; Schmidt et al., 2012; Veroude et al., 2013; Verstynen, 2014; Barkley-Levenson et al., 2018; Kühn et al., 2016; Jaspar et al., 2014; Overbeek et al., 2019; Purmann & Pollmann, 2015; Peven et al., 2019; Ning et al., 2021; Li et al., 2019)
R anterior insula/orbitofrontal cortex	36 20 0	Id7, Id7	
**Cluster 5 (k=338)**			13 experiments (Ye et al., 2009; Brass et al., 2005; Grandjean et al., 2012/2013; Zhu et al., 2013; Hough et al., 2016; Manard et al., 2017; Schmidt et al., 2012; Coderre et al., 2013; Jaspar et al., 2014; Peven et al., 2019; Papalini et al., 2019; Ning et al., 2021, Chen, Meng et al., 2023)
L fusiform gyrus	-44 -56 -16	FG4, FG2	
**Cluster 6 (k=326)**			18 experiments (Ye et al., 2009; Becker et al., 2008; Grandjean et al., 2012/2013; Prakash et al., 2009B; Fan et al., 2003; Potenza et al., 2003; DeVito et al., 2012; Hough et al., 2016; Manard et al., 2017; Schmidt et al., 2012; Veroude et al., 2013; Verstynen, 2014; Barkley-Levenson et al., 2018; Jaspar et al., 2014; Morgenroth et al., 2019; Purmann & Pollmann, 2015; Ning et al., 2021; Li et al., 2019)
L anterior insula	-34 20 0	Id7, Id6	
**Cluster 7 (k=275)**			16 experiments (Kerns et al., 2005; Ye et al., 2009; Brass et al., 2005; Prakash et al., 2009B; Milham et al., 2001; Potenza et al., 2003; Huang et al, 2017; Lesh et al., 2013; Schmidt et al., 2012; Veroude et al., 2013; Verstynen, 2014; Barkley-Levenson et al., 2018; Jaspar et al., 2014; Overbeek et al., 2019; Purmann & Pollmann, 2015; Peven et al., 2019)
R inferior/middle frontal gyrus	46 14 30		
**Cluster 8 (k=116)**			10 experiments (Ye et al., 2009; Grandjean et al., 2012/2013; Potenza et al., 2003; DeVito et al., 2012; Manard et al., 2017; Schmidt et al., 2012; Veroude et al., 2013; Coderre et al., 2013; Peven et al., 2019; Li et al., 2019)
R intraparietal sulcus	36 -52 44	hIP1, hIP3	

Table S9

Clusters of significant convergence in the meta-analyses across experiments with and without additional cognitive demand, their center of mass coordinate, histological assignments as well as experiments contributing.

Brain Structure	MNI Coordinates	Histological Assignments	Contributions
**Additional demand**			
**Cluster 1 (k=518)**			16 experiments (Zysset et al., 2001; Basten et al., 2011; Kim et al., 2011; Zysset et al., 2007; Norris et al., 2002; Kim et al., 2014; Köhler et al., 2016; Mathis et al., 2009A; Mathis et al., 2009B; Mathis et al., 2009C; Schulte et al., 2012; Wallentin et al., 2015; Shin & Kim, 2015; Agostini et al., 2017; Shashidhara et al., 2010; Gianaros et al., 2008)
L inferior/middle frontal gyrus	-44 14 28	44	
**Cluster 2 (k=269)**			13 experiments (Zysset et al., 2001; Basten et al., 2011; Zysset et al., 2007; Norris et al., 2002; Kim et al., 2014; Köhler et al., 2016; Mathis et al., 2009B; Mathis et al., 2009C; Shin & Kim 2015; Agostini et al., 2017; Wagner et al., 2015; Sheu et al., 2012; Gianaros et al., 2008)
Posterior medial frontal cortex	0 18 48		
**Cluster 3 (k=242)**			11 experiments (Basten et al., 2011; Kim et al., 2011; Zysset et al., 2007; Kim et al., 2014; Köhler et al., 2016; Mathis et al., 2009C; Shin & Kim, 2015; Wagner et al., 2015; Shashidhara et al., 2020; Sheu et al., 2012; Gianaros et al., 2008)
R anterior insula/orbitofrontal cortex	36 21 0	Id7, Id6	
**Cluster 4 (k=235)**			11 experiments (Zysset et a., 2001; Zysset et al., 2007; Kim et al., 2014; Köhler et al., 2016; Mathis et al., 2009B; Mathis et al., 20009C; Wallentin et al., 2015; Shin & Kim, 2015; Wagner et al., 2015; Sheu et al., 2012; Gianaros et al., 2008)
L anterior insula	-32 24 0	Id7	
**Cluster 5 (k=216)**			14 experiments (Zysset et al., 2001; Schulte et al., 2009; Basten et al., 2011; Kim et al., 2011; Zysset et al., 2007; Norris et al., 2002; Kim et al., 2014; Mathis et al., 2009A; Mathis et al., 2009B; Mathis et al., 2009C; Shin & Kim, 2015; Wagner et al., 2015; Shashidhara et al., 2010; Sheu et al., 2012)
R inferior frontal gyrus	46 14 28		
**Cluster 6 (k=201)**			12 experiments (Basten et al., 2011; Kim et al., 2011; Zysset et al., 2007; Norris et al., 2002; Mathis et al., 2009A; Mathis et al., 2009B; Schulte et al., 2012; Wallentin et al., 2015; Shin & Kim, 2015; Agostini et al., 2017; Wagner et al., 2015; Sheu et al., 2012)
L intraparietal sulcus	-30 -58 44	hIP3, hIP6	

**Cluster 7 (k=126)**			11 experiments (Zysset et al., 2001; Schulte et al., 2009; Basten et al. 2011; Kim et al., 2011; Zysset et al., 2007; Norris et al., 2002; Mathis et al., 2009A; Mathis et al., 2009B; Wallentin et al., 2015; Agostini et al., 2017: Shashidhara et al., 2020)
R intraparietal sulcus	34 -56 46	hIP3, hIP6, hIP1	
**Cluster 8 (k=108)**			
R middle frontal gyrus	44 28 28		10 experiments (Zysset et al., 2001; Schulte et al., 2009; Kim et al., 2011; Zysset et al., 2007; Norris et al., 2002; Mathis et al., 2009A; Mathis et al., 2009B; Mathis et al., 2009C; Wallentin et al., 2015; Wagner et al., 2016)
**No additional demand**			
**Cluster 1 (k=1479)**			53 experiments (Kerns et al., 2005; Pardo et al., 1990; Banich et al., 2001; Adleman et al., 2002; Ye et al., 2009; Brass et al., 2005; Polk et al., 2008; Becker et al., 2008; Grandjean et al., 2012/2013; Prakash et al., 2009A, Prakash et al., 2009B; Zhu et al., 2013; Fan et al., 2003; Coderre et al., 2008; Silton et al., 2010; Taylor et al., 1997A; Taylor et al., 1997B; Milham et al., 2001; Ruff et al., 2001; Ravnkilde et al., 2002; Nakao et al., 2005; Zoccatelli et al., 2010; Mead et al., 2002; Chen et al., 2018; Fechir et al., 2010; Lesh et al., 2013; Manard et al., 2017; Pompei et al., 2011; Schmidt et al., 2012; Song et al., 2015; Van't Ent et al., 2014; Veroude et al., 2013; Verstynen, 2014; Coderre et al., 2013; Barkley-Levenson et al., 2018; Kühn et al., 2016; Jaspar et al., 2014; Morgenroth et al., 2019; Overbeek et al., 2019; Portes et al., 2019A; Portes et al., 2019B; Purmann & Pollmann, 2015; Peven et al., 2019; Kozasa et al., 2018; Ghavidel et al., 2020; Roberts & Hall, 2008; Papalini et al., 2019; van de Meerendonk et al., 2013; Ning et al., 2021; Li et al., 2019; Salgado-Pineda et al., 2002; Puente et al., 2014; Chen Meng et al., 2023)
L inferior/middle frontal gyrus	-44 14 30	44	
**Cluster 2 (k=1343)**			50 experiments (Bench et al., 1993; Banich et al., 2001; Adleman et al., 2002; Carter et al., 1995; Ye et al., 2009; Polk et al., 2008; Becker et al., 2008; Grandjean et al., 2012/2013; Mitchell, 2005; Prakash et al., 2009A, Prakash et al., 2009B; Zhu et al., 2013; Fan et al., 2003; Coderre et al., 2008; Silton et al., 2010; Taylor et al., 1997; George et al., 1994; Milham et al., 2001; Ruff et al., 2001; Potenza et al., 2003; Nakao et al., 2005; Zoccatelli et al., 2010; Chen et al., 2018; DeVito et al., 2012; Fechir et al., 2010; Lesh et al., 2013; Manard et al., 2017; Pompei et al., 2011; Schmidt et al., 2012; Song et al.,
L intraparietal sulcus	-32 -56 46	hIP3, hIp1, hIP6, hIP2	

			2015; Terry et al., 2012; Van't Ent et al., 2014; Veroude et al., 2013; Verstynen, 2014; Coderre et al., 2013; Barkley-Levenson et al., 2018; Jaspar et al., 2014; Overbeek et al., 2019; Portes et al., 2019A; Purmann & Pollmann, 2015; Peven et al., 2019; Kozasa et al., 2018; Roberts & Hall, 2008; Papalini et al., 2019; van de Meerendonk et al., 2013; Ning et al., 2021; Li et al., 2019; Salgado-Pineda et al., 2002; Puente et al., 2014; Chen, Meng et al., 2013)
**Cluster 3 (k=1262)**			43 experiments (Kerns et al., 2005; Pardo et al., 1990; Banich et al., 2001; Carter et al., 1995; Ye et al., 2009; Brass et al., 2005; Polk et al., 2008; Becker et al., 2008; Grandjean et al., 2012/2013; Zhu et al., 2013; Fan et al., 2003; Coderre et al., 2008; Silton et al., 2010; Milham et al., 2001; Ruff et al., 2001; Steel et al., 2001; Ravnkilde et al., 2002; Potenza et al., 2003; Nakao et al., 2005; Zoccatelli et al., 2010; Chen et al., 2018; DeVito et al., 2012; Fechir et al., 2010; Lesh et al., 2013; Piai et al., 2013; Schmidt et al., 2012; Veroude et al., 2013; Verstynen, 2014; Coderre et al., 2013; Barkley-Levenson et al., 2018; Kühn et al., 2016; Jaspar et al., 2014; Morgenroth et al., 2019; Overbeek et al., 2019; Purmann & Pollmann, 2015; Peven et al., 2019; Ghavidel et al., 2020; Roberts & Hall, 2008; Papalini et al., 2019; Ning et al., 2021; Li et al., 2019; Puente et al., 2014; Chen, Meng et al., 2023)
Posterior medial frontal cortex	0 18 46	6mr	
**Cluster 4 (k=761)**			32 experiments (Bench et al., 1993; Ye et al., 2009; Brass et al., 2005; Polk et al., 2008; Grandjean et al., 2012/2013; Prakash et al., 2009B; Zhu et al., 2013; Silton et al., 2010; Taylor et al., 1997A; George et al, 1994; Steel et al., 2001; Potenza et al., 2003; Nakao et al., 2005; Zoccatelli et al., 2010; Chen et al., 2018; DeVito et al., 2012; Hough et al., 2016; Manard et al., 2017; Piai et al., 2013; Pompei et al., 2011; Schmidt et al., 2012; Veroude et al., 2013; Verstynen, 2014; Barkley-Levenson et al., 2018; Kühn et al., 2016; Jaspar et al., 2014; Overbeek et al., 2019; Portes et al., 2019B; Purmann & Pollmann, 2015; Peven et al., 2019; Ning et al., 2021; Li et al., 2019)
R anterior insula	36 22 -2	Id6, Id7	
**Cluster 5 (k=473)**			27 experiments (Pardo et al., 1990; Banich et al., 2001; Ye et al., 2009; Polk et al., 2008; Becker et al., 2008; Grandjean et al., 2012/2013; Mitchell, 2005; Prakash et al., 2009B; Silton et al., 2010; Taylor et al., 1997A; George et al., 1994; Steel et al., 2001; Ravnkilde et al., 2002; Potenza et al., 2003; Zoccatelli et al., 2010; DeVito et al., 2012; Hough et al., 2016; Manard et al., 2017; Schmidt et al., 2012; Veroude et al., 2013; Verstynen, 2014; Barkley-
L anterior insula	-34 20 -2	Id6, Id7	

			Levenson et al., 2018; Jaspar et al., 2014; Morgenroth et al., 2019; Purmann & Pollmann, 2015; Ning et al., 2021; Li et al., 2019)
**Cluster 6 (k=380)**			24 experiments (Kerns et al., 2005; Ye et al., 2009; Brass et al., 2005; Prakash et al., 2009B; Fan et al., 2003; Coderre et al., 2008; Taylor et al., 1997A; Milham et al., 2001; Potenza et al., 2003; Nakao et al., 2005; Zoccatelli et al., 2010; Huang et al., 2016; ; Lesh et al., 2013; Schmidt et al., 2012; Song et al., 2015; Van't Ent et al., 2014; Veroude et al., 2013; Verstynen, 2014; Barkley-Levenson et al., 2018; Jaspar et al., 2014; Overbeek et al., 2019; Purmann & Pollmann, 2015; Peven et al., 2019; Roberts & Hall, 2008)
R inferior/middle frontal gyrus	46 16 28		
**Cluster 7 (k=267)**			15 experiments (Ye et al., 2009; Brass et al., 2005; Grandjean et al., 2012/2013; Zhu et al., 2013; Silton et al., 2010; Hough et al., 2016; Manard et al., 2017; Schmidt et al., 2012; Van't Ent et al., 2014; Coderre et al., 2013; Jaspar et al., 2014; Peven et al., 2019; Papalini et al., 2019; Ning et al., 2021; Chen, Meng et al., 2023)
L fusiform gyrus	-46 -56 -16	FG4, FG2	
**Cluster 8 (k=255)**			19 experiments (Bench et al., 1993; Ye et al., 2009; Polk et al., 2008; Grandjean et al., 2012/2013; Silton et al., 2010; Coderre et al., 2008; Potenza et al., 2003; Nakao et al., 2005; Zoccatelli et al., 2010; DeVito et al., 2012; Manard et al., 2017; Pompei et al., 2011; Schmidt et al., 2012; Terry et al., 2012; Van't Ent et al., 2014; Veroude et al., 2013; Coderre et al., 2013; Peven et al., 2019; Li et al., 2019)
R intraparietal sulcus	38 -50 46	hIP1, hIP3	

Table S10

Clusters of significant convergence in the meta-analyses across experiments employing a color-word (please note that this is the same meta-analysis and therefore the same results as for I>C but presented here again for better comparisons between stimulus material types) , emotional or other type of Stroop task, their center of mass coordinate, histological assignments as well as experiments contributing.

Brain Structure	MNI Coordinates	Histological Assignments	Contributions
**Color-word Stroop**			
**Cluster 1 (k=1111)**			36 experiments (Kerns et al., 2005; Pardo et al., 1990; Ye et al., 2005; Brass et al., 2005; Becker et al., 2008; Grandjean et al., 2012/2013; Zhu et al., 2013; Fan et al., 2003; Coderre et al., 2008; Silton et al, 2010; Ravnkilde et al., 2002; Nakao et al., 2005; Zoccatelli et al., 2010; Mead et al., 2002; Chen et al., 2018; Fechir et al., 2010; Lesh et al., 2013; Schmidt et al., 2012; Van't Ent et al., 2014; Barkley-Levenson et al., 2018; Kühn et al., 2016; Morgenroth et al., 2019; Overbeek et al., 2019; Portes et al., 2019A; Portes et al., 2019B; Purmann & Pollmann, 2015; Peven et al., 2019; Kozasa et al, 2018; Ghavidel et al., 2020; Papalini et al., 2019; Coderre et al., 2013; Li et al., 2019; Ning et al., 2021; Salgado-Pineda et al., 2002; Puente et al., 2014; Chen, Meng et al., 2023)
L inferior/middle frontal gyrus	-44 12 28	44	
**Cluster 2 (k=1007)**			32 experiments (Kerns et al., 2005; Pardo et al., 1990; Carter et al., 1995; Ye et al., 2005; Brass et al., 2005; Becker et al., 2008; Zhu et al., 2003; Fan et al., 2003; Coderre et al., 2008; Silton et al, 2010; Ravnkilde et al., 2002; Potenza et al., 2003; Nakao et al., 2005; Zoccatelli et al., 2010; Chen et al., 2018; DeVito et al., 2012; Fechir et al., 2010; Lesh et al., 2013; Schmidt et al., 2012; Barkley-Levenson et al., 2018; Kühn et al., 2016; Morgenroth et al., 2019; Overbeek et al., 2019; Portes et al., 2019A; Purmann & Pollmann, 2015; Ghavidel et al., 2020; Papalini et al., 2019; Coderre et al., 2013; Li et al., 2019; Ning et al., 2021; Puente et al., 2014; Chen, Meng et al., 2023)
Posterior medial frontal cortex	0 18 46	6mr	
**Cluster 3 (k=637)**			30 experiments (Carter et al. 1995; Ye et al., 2009; Becker et al., 2008; Grandjean et al., 2012/2013; Zhu et al., 2013; Fan et al., 2003; Coderre et al., 2008; Silton et al, 2010; Potenza et al., 2003; Nakao et al., 2005; Zoccatelli et al., 2010; Chen et al., 2018; DeVito et al., 2012; Fechir et al., 2010; Lesh et al., 2013; Schmidt et al., 2012; Terry et al., 2012; Van't Ent et al., 2014;
L intraparietal sulcus	-32 -56 46	hIP3, hIP1, hIP6	

			Barkley-Levenson et al., 2018; Overbeek et al., 2019; Portes et al., 2019A; Purmann & Pollmann, 2015; Kozasa et al, 2018; Papalini et al., 2019; Coderre et al., 2013; Li et al., 2019; Ning et al., 2021, Salgado-Pineda et al., 2002; Puente et al., 2014; Chen, Meng et al., 2023)
**Cluster 4 (k=390)**			19 experiments (Ye et al., 2009; Brass et al., 2005; Zhu et al., 2013; Silton et al., 2010; Potenza et al., 2003; Nakao et al., 2005; Zoccatelli et al., 2010; Chen et al., 2018; DeVito et al., 2012; Hough et al.. 2016; Piai et al., 2013; Schmidt et al., 2012; Barkley-Levenson et al., 2018; Kühn et al., 2016; Overbeek et al., 2019; Portes et al., 2019B; Purmann & Pollmann, 2015; Li et al., 2019; Ning et al., 2021)
R anterior insula	38 22 -3	Id6; Id7	
**Cluster 5 (k=266)**			15 experiments (Pardo et al., 1990; Ye et al., 2009; Becker et al., 2008; Silton et al., 2010; Ravnkilde et al., 2002; Potenza et al., 2003; Zoccatelli et al., 2010; DeVito et al., 2012; Hough et al., 2016; Schmidt et al., 2012; Barkley-Levenson et al., 2018; Morgenroth et al., 2019; Purmann & Pollmann, 2015; Li et al., 2019; Ning et al., 2021)
L anterior insula	-34 20 -3	Id7, Id6	
**Cluster 6 (k=218)**			12 experiments (Ye et al., 2009; Brass et al., 2005; Grandjean et al., 2012/2013; Zhu et al, 2013; Silton et al., 2010; Hough et al., 2016; Schmidt et al., 2012; Van't Ent et al., 2014; Peven et al., 2018; Papalini et al., 2019; Ning et al., 2021; Chen, Meng et al., 2023)
L fusiform gyrus	-48 -56 -16	FG4, FG2	
**Cluster 7 (k=107)**			12 experiments (Kerns et al., 2005; Brass et al., 2005; Coderre et al., 2008; Potenza et al., 2003; Nakao et al., 2003; Zoccatelli et al., 2010; Lesh et al., 2013; Schmidt et al., 2012; Van't Ent et al., 2014; Barkley-Levenson et al., 2018; Overbeek et al., 2019; Purmann & Pollmann, 2015)
R inferior/middle frontal gyrus	48 22 26		
**Cluster 8 (k=98)**			7 experiments (Fan et al., 2003; Silton et al., 2010; Fechir et al., 2010, Terry et al., 2012; Purmann & Pollmann, 2015; Papalini et al., 2019; Puente et al., 2014)
Precuneus	-4 -60 45		
**Emotional Stroop**			
**Cluster 1 (k=241)**			10 experiments (Jarcho et al., 2013; Chechko et al., 2012; Chechko et al., 2009; Krug et al., 2012; Chechko et al., 2013; Godinez et al., 2016; Bayer et al., 2018; Hassel et al., 2020; Kohn et al., 2020; Papalini et al., 2019)
L intraparietal sulcus	-28 -60 46	hIP3, hIP6	
**Cluster 2 (k=226)**			

Posterior medial frontal gyrus	8 22 36		7 experiments (Chechko et al., 2012; Krug et al., 2012; Chechko et al., 2013; Bayer et al., 2018; Hassel et al., 2020; Kohn et al., 2020; Almdahl et al., 2021)
**Cluster 3 (k=224)**			9 experiments (Chechko et al., 2012; Chechko et al., 2009; Fleury et al., 2014; Krug et al., 2012; Chechko et al., 2013; Godinez et al., 2016; Kohn et al., 2020; Bang et al., 2016; Almdahl et al., 2021)
L inferior/middle frontal gyrus	-46 18 24		
**Cluster 4 (k=199)**			10 experiments (Ovaysikia et al., 2011; Chechko et al., 2012; Chechko et al., 2009; Krug et al., 2012; Chechko et al., 2013; Bayer et al., 2018; Kohn et al., 2020; Löffler et al., 2019; Bang et al., 2016; Chen et al., 2023)
Posterior medial frontal gyrus	-2 14 54	6mr	
**Cluster 5 (k=192)**			8 experiments (Jarcho et al., 2003; Chechko et al., 2012; Fleury et al., 2014; Krug et al., 2012; Chechko et al., 2013; Bayer et al., 2018; Bang et al., 2016; Papalini et al., 2019)
R anterior insula	34 20 2	Id6, Id7, OP8	
**Cluster 6 (k=146)**			9 experiments (Chechko et al., 2012; Chechko et al., 2009; Krug et al., 2012; Godinez et al., 2016; Hassel et al., 2020; Kohn et al., 2020; Löffler et al., 2019; Bang et al., 2016; Almdahl et al., 2021)
L dorsal premotor cortex	-40 -2 46		
**Cluster 7 (k=83)**			6 experiments (Krug et al., 2012; Chechko et al., 2013; Hassel et al., 2020; Kohn et al., 2020; Bang et al., 2016; Papalini et al., 2019)
R inferior middle frontal gyrus	50 22 24	44,45	
**Other types of Stroop**			
**Cluster 1 (k=215)**			11 experiments (Roth et al., 2006; Matthews et al., 2004; Ansari et al., 2006; Huang et al., 2012; Robertson et al., 2015; Chechko et al., 2012; Ramm et al., 2021; Hoogeveen et al., 2020; Fedelli et al., 2022; Wang et al., 2023; Bohle, 2016)
R anterior insula	36 20 0	Id6, Id7,OP8	
**Cluster 2 (k=152)**			9 experiments (Ansari et al., 2006, Huang et al.,2012; Kaufmann et al., 2005 Chechko et al., 2012; Chechko et al., 2013; Krönke et al., 2018; Fedelli et al., 2022; Verdolini et al., 2023; Bohle, 2016)
Posterior medial frontal cortex	6 22 36		
**Cluster 3 (k=103)**			7 experiments (Hart et al., 2010; Huang et al., 2012; Robertson et al., 2015; Krönke et al., 2018; Pinel et al., 2004; Hoogeveen et al., 2020; Fan et al., 2017)
L intraparietal sulcus	-42 -38 44	hIP2, hIP1	

**Cluster 4 (k=89)**			8 experiments (Matthews et al., 2004; Robertson et al., 2015; Krönke et al., 2018; Ramm et al., 2021; Hoogeveen et al., 2020; Salgado-Pineda et al., 2021; Fedelli et al., 2022; Bohle, 2016)
Pre supplementary motor area	-64 54	6mr	

Figure S1

Meta-analysis across experiments using words or symbols/letters as neutral control conditions (A) and contrast analyses of both meta-analyses to the analysis using congruent conditions as control (B).

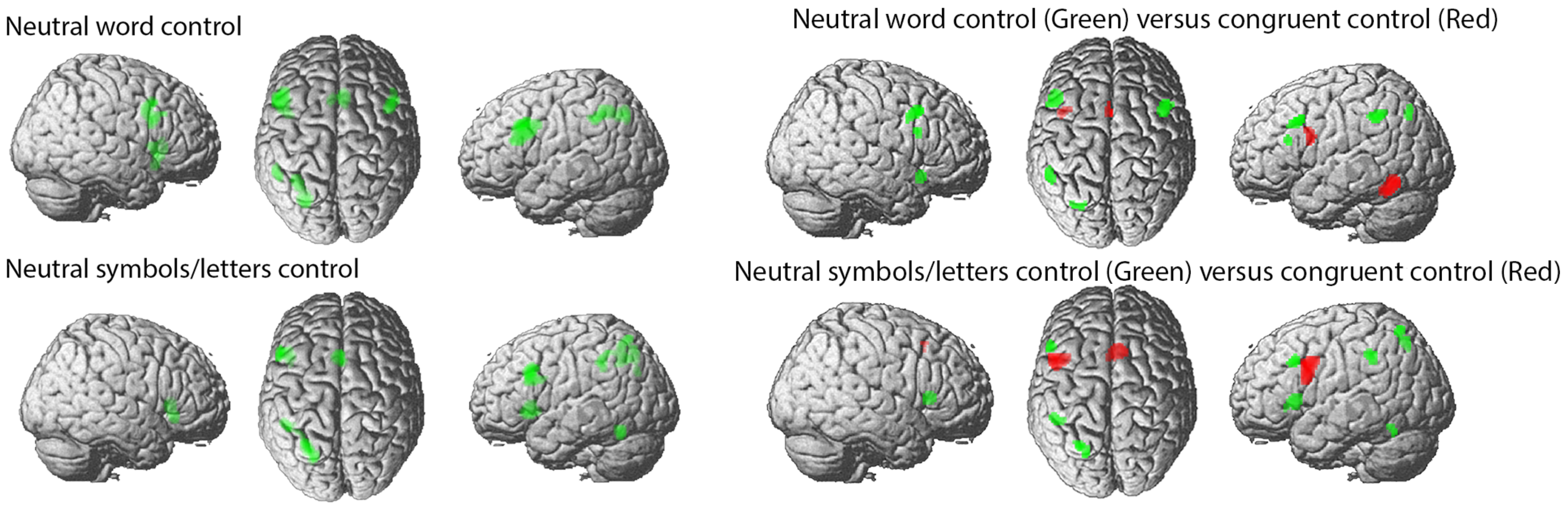

Reference list of all studies included in the meta-analyses

**included in neuroimaging and behavioral meta-analysis; *included in neuroimaging meta-analysis only

*Adleman, N. E., Menon, V., Blasey, C. M., White, C. D., Warsofsky, I. S., Glover, G. H., & Reiss, A. L. (2002, May). A developmental fMRI study of the Stroop color-word task. Neuroimage, 16(1), 61-75. https://doi.org/10.1006/nimg.2001.1046

**Agostini, A., Ballotta, D., Righi, S., Moretti, M., Bertani, A., Scarcelli, A., Sartini, A., Ercolani, M., Nichelli, P., Campieri, M., & Benuzzi, F. (2017, Oct). Stress and brain functional changes in patients with Crohn's disease: A functional magnetic resonance imaging study. Neurogastroenterology and Motility, 29(10), 1-10. https://doi.org/10.1111/nmo.13108

**Almdahl, I. S., Martinussen, L. J., Agartz, I., Hugdahl, K., & Korsnes, M. S. (2021, May). Inhibition of emotions in healthy aging: age-related differences in brain network connectivity. Brain and Behavior, 11(5), e02052. https://doi.org/10.1002/brb3.2052

**Ansari, D., Fugelsang, J. A., Dhital, B., & Venkatraman, V. (2006, Aug 15). Dissociating response conflict from numerical magnitude processing in the brain: an event-related fMRI study. Neuroimage, 32(2), 799-805. https://doi.org/10.1016/j.neuroimage.2006.04.184

**Bang, L., Ro, O., & Endestad, T. (2016, Feb 28). Amygdala alterations during an emotional conflict task in women recovered from anorexia nervosa. Psychiatry Research Neuroimaging, 248, 126-133. https://doi.org/10.1016/j.pscychresns.2015.12.008

*Banich, M. T., Milham, M. P., Jacobson, B. L., Webb, A., Wszalek, T., Cohen, N. J., & Kramer, A. F. (2001). Attentional selection and the processing of task-irrelevant information: insights from fMRI examinations of the Stroop task. Progress in Brain Research, 134, 459-470. https://doi.org/10.1016/s0079-6123(01)34030-x

*Barkley-Levenson, E., Xue, F., Droutman, V., Miller, L. C., Smith, B. J., Jeong, D., Lu, Z. L., Bechara, A., & Read, S. J. (2018, Apr 19). Prefrontal Cortical Activity During the Stroop Task: New Insights into the Why and the Who of Real-World Risky Sexual Behavior. Annals of Behavioral Medicine, 52(5), 367-379. https://doi.org/10.1093/abm/kax019

**Basten, U., Stelzel, C., & Fiebach, C. J. (2011, Oct). Trait anxiety modulates the neural efficiency of inhibitory control. Journal of Cognitive Neuroscience, 23(10), 3132-3145. https://doi.org/10.1162/jocn_a_00003

**Bayer, M., Rubens, M. T., & Johnstone, T. (2018, Feb). Simultaneous EEG-fMRI reveals attention-dependent coupling of early face processing with a distributed cortical network. Biological Psychology, 132, 133-142. https://doi.org/10.1016/j.biopsycho.2017.12.002

**Becker, T. M., Kerns, J. G., Macdonald, A. W., 3rd, & Carter, C. S. (2008, Oct). Prefrontal dysfunction in first-degree relatives of schizophrenia patients during a Stroop task. Neuropsychopharmacology, 33(11), 2619-2625. https://doi.org/10.1038/sj.npp.1301673

**Bench, C. J., Frith, C. D., Grasby, P. M., Friston, K. J., Paulesu, E., Frackowiak, R. S., & Dolan, R. J. (1993, Sep). Investigations of the functional anatomy of attention using the Stroop test. Neuropsychologia, 31(9), 907-922. https://doi.org/10.1016/0028-3932(93)90147-r

**Bohle, H. S. (2016, July). Cognitive conflicts in the Stroop paradigm: Information retrieval and response selection in the human cognitive system. [Dissertation, Humbold-Universtität zu Berlin]. Berlin. http://edoc.hu-berlin.de/18452/18238

*Brass, M., Derrfuss, J., & von Cramon, D. Y. (2005). The inhibition of imitative and overlearned responses: a functional double dissociation. Neuropsychologia, 43(1), 89-98. https://doi.org/10.1016/j.neuropsychologia.2004.06.018

**Carter, C. S., Mintun, M., & Cohen, J. D. (1995, Dec). Interference and facilitation effects during selective attention: an H215O PET study of Stroop task performance. Neuroimage, 2(4), 264-272. https://doi.org/10.1006/nimg.1995.1034

**Chechko, N., Augustin, M., Zvyagintsev, M., Schneider, F., Habel, U., & Kellermann, T. (2013, Jul). Brain circuitries involved in emotional interference task in major depression disorder. Journal of Affective Disorders, 149(1-3), 136-145. https://doi.org/10.1016/j.jad.2013.01.013

**Chechko, N., Kellermann, T., Zvyagintsev, M., Augustin, M., Schneider, F., & Habel, U. (2012). Brain circuitries involved in semantic interference by demands of emotional and non-emotional distractors. PloS One, 7(5), e38155. https://doi.org/10.1371/journal.pone.0038155

**Chechko, N., Wehrle, R., Erhardt, A., Holsboer, F., Czisch, M., & Samann, P. G. (2009, May 20). Unstable prefrontal response to emotional conflict and activation of lower limbic structures and brainstem in remitted panic disorder. PloS One, 4(5), e5537. https://doi.org/10.1371/journal.pone.0005537

*Chen, Z., Zhao, X., Fan, J., & Chen, A. (2018, Dec). Functional cerebral asymmetry analyses reveal how the control system implements its flexibility. Human Brain Mapping, 39(12), 4678-4688. https://doi.org/10.1002/hbm.24313

**Chen, Y. H., Chen, C., Jian, H. Y., Chen, Y. C., Fan, Y. T., Yang, C. Y., & Cheng, Y. (2023). The neural correlates of emotional conflict monitoring as an early manifestation of affective and cognitive declines in persons with Type 2 diabetes. Brain Commun, 5(1), fcad022. https://doi.org/10.1093/braincomms/fcad022

* Chen, Q., Meng, Z., Xu, L., Hou, Y., & Chen, A. (2023, Mar). Effective connectivity analysis reveals the time course of the Stroop effect in manual responding. Biological Psychology, 178, 108526. https://doi.org/10.1016/j.biopsycho.2023.108526

*Coderre, E. L., Filippi, C. G., Newhouse, P. A., & Dumas, J. A. (2008, Nov). The Stroop effect in kana and kanji scripts in native Japanese speakers: an fMRI study. Brain and Language, 107(2), 124-132. https://doi.org/10.1016/j.bandl.2008.01.011

**Coderre, E. L., & van Heuven, W. J. (2013, Jul 31). Modulations of the executive control network by stimulus onset asynchrony in a Stroop task. BMC Neuroscience, 14, 79. https://doi.org/10.1186/1471-2202-14-79

*DeVito, E. E., Worhunsky, P. D., Carroll, K. M., Rounsaville, B. J., Kober, H., & Potenza, M. N. (2012, May 1). A preliminary study of the neural effects of behavioral therapy for substance use disorders. Drug and Alcohol Dependence, 122(3), 228-235. https://doi.org/10.1016/j.drugalcdep.2011.10.002

*Fan, J., Flombaum, J. I., McCandliss, B. D., Thomas, K. M., & Posner, M. I. (2003, Jan). Cognitive and brain consequences of conflict. Neuroimage, 18(1), 42-57. https://doi.org/10.1006/nimg.2002.1319

** Fan, L. Y., Chou, T. L., & Gau, S. S. (2017, Oct). Neural correlates of atomoxetine improving inhibitory control and visual processing in Drug-naive adults with attention-deficit/hyperactivity disorder. Human Brain Mapping, 38(10), 4850-4864. https://doi.org/10.1002/hbm.23683

**Fechir, M., Gamer, M., Blasius, I., Bauermann, T., Breimhorst, M., Schlindwein, P., Schlereth, T., & Birklein, F. (2010, Apr 1). Functional imaging of sympathetic activation during mental stress. Neuroimage, 50(2), 847-854. https://doi.org/10.1016/j.neuroimage.2009.12.004

** Fedeli, D., Del Maschio, N., Del Mauro, G., Defendenti, F., Sulpizio, S., & Abutalebi, J. (2022, Aug 11). Cingulate cortex morphology impacts on neurofunctional activity and behavioral performance in interference tasks. Scientific Reports, 12(1), 13684. https://doi.org/10.1038/s41598-022-17557-6

**Fleury, V., Cousin, E., Czernecki, V., Schmitt, E., Lhommee, E., Poncet, A., Fraix, V., Tropres, I., Pollak, P., Krainik, A., & Krack, P. (2014). Dopaminergic modulation of emotional conflict in Parkinson's disease. Frontiers in Aging Neuroscience, 6, 164. https://doi.org/10.3389/fnagi.2014.00164

*George, M. S., Ketter, T. A., Parekh, P. I., Rosinsky, N., Ring, H., Casey, B. J., Trimble, M. R., Horwitz, B., Herscovitch, P., & Post, R. M. (1994). Regional brain activity when selecting a response despite interference: An H2 (15) O PET study of the stroop and an emotional stroop. Human Brain Mapping, 1(3), 194-209. https://doi.org/10.1002/hbm.460010305

*Ghavidel, N., Khodagholi, F., Ahmadiani, A., Khosrowabadi, R., Asadi, S., & Shams, J. (2020). Frontocingulate Dysfunction Is Associated with Depression and Decreased Serum PON1 in Methamphetamine-Dependent Patients. Neuropsychiatric Disease and Treatment, 16, 489-499. https://doi.org/10.2147/NDT.S237528

*Gianaros, P. J., Sheu, L. K., Matthews, K. A., Jennings, J. R., Manuck, S. B., & Hariri, A. R. (2008, Jan 23). Individual differences in stressor-evoked blood pressure reactivity vary with activation, volume, and functional connectivity of the amygdala. Journal of Neuroscience, 28(4), 990-999. https://doi.org/10.1523/JNEUROSCI.3606-07.2008

**Godinez, D. A., McRae, K., Andrews-Hanna, J. R., Smolker, H., & Banich, M. T. (2016, Dec). Differences in frontal and limbic brain activation in a small sample of monozygotic twin pairs discordant for severe stressful life events. Neurobiology of Stress, 5, 26-36. https://doi.org/10.1016/j.ynstr.2016.10.002

**Grandjean, J., D'Ostilio, K., Fias, W., Phillips, C., Balteau, E., Degueldre, C., Luxen, A., Maquet, P., Salmon, E., & Collette, F. (2013, May). Exploration of the mechanisms underlying the ISPC effect: evidence from behavioral and neuroimaging data. Neuropsychologia, 51(6), 1040-1049. https://doi.org/10.1016/j.neuropsychologia.2013.02.015

**Grandjean, J., D'Ostilio, K., Phillips, C., Balteau, E., Degueldre, C., Luxen, A., Maquet, P., Salmon, E., & Collette, F. (2012). Modulation of brain activity during a Stroop inhibitory task by the kind of cognitive control required. PloS One, 7(7), e41513. https://doi.org/10.1371/journal.pone.0041513

**Hart, S. J., Green, S. R., Casp, M., & Belger, A. (2010, Feb 1). Emotional priming effects during Stroop task performance. Neuroimage, 49(3), 2662-2670. https://doi.org/10.1016/j.neuroimage.2009.10.076

*Hassel, S., Sharma, G. B., Alders, G. L., Davis, A. D., Arnott, S. R., Frey, B. N., Hall, G. B., Harris, J. K., Lam, R. W., Milev, R., Muller, D. J., Rotzinger, S., Zamyadi, M., Kennedy, S. H., Strother, S. C., & MacQueen, G. M. (2020, Apr 15). Reliability of a functional magnetic resonance imaging task of emotional conflict in healthy participants. Human Brain Mapping, 41(6), 1400-1415. https://doi.org/10.1002/hbm.24883

**Hinault, T., Larcher, K., Zazubovits, N., Gotman, J., & Dagher, A. (2019, Jan). Spatio-temporal patterns of cognitive control revealed with simultaneous electroencephalography and functional magnetic resonance imaging. Human Brain Mapping, 40(1), 80-97. https://doi.org/10.1002/hbm.24356

**Hoogeveen, S., Snoek, L., & van Elk, M. (2020, Aug). Religious belief and cognitive conflict sensitivity: A preregistered fMRI study. Cortex, 129, 247-265. https://doi.org/10.1016/j.cortex.2020.04.011

**Hough, C. M., Luks, T. L., Lai, K., Vigil, O., Guillory, S., Nongpiur, A., Fekri, S. M., Kupferman, E., Mathalon, D. H., & Mathews, C. A. (2016, Sep 30). Comparison of brain activation patterns during executive function tasks in hoarding disorder and non-hoarding OCD. Psychiatry Research Neuroimaging, 255, 50-59. https://doi.org/10.1016/j.pscychresns.2016.07.007

**Huang, C. M., Polk, T. A., Goh, J. O., & Park, D. C. (2012, Jan). Both left and right posterior parietal activations contribute to compensatory processes in normal aging. Neuropsychologia, 50(1), 55-66. https://doi.org/10.1016/j.neuropsychologia.2011.10.022

**Huang, S., Zhu, Z., Zhang, W., Chen, Y., & Zhen, S. (2017). Trait impulsivity components correlate differently with proactive and reactive control. PloS One, 12(4), e0176102. https://doi.org/10.1371/journal.pone.0176102

**Jarcho, J. M., Fox, N. A., Pine, D. S., Etkin, A., Leibenluft, E., Shechner, T., & Ernst, M. (2013, Feb). The neural correlates of emotion-based cognitive control in adults with early childhood behavioral inhibition. Biological Psychology, 92(2), 306-314. https://doi.org/10.1016/j.biopsycho.2012.09.008

**Jaspar, M., Genon, S., Muto, V., Meyer, C., Manard, M., Dideberg, V., Bours, V., Salmon, E., Maquet, P., & Collette, F. (2014, Jan). Modulating effect of COMT genotype on the brain regions underlying proactive control process during inhibition. Cortex, 50, 148-161. https://doi.org/10.1016/j.cortex.2013.06.003

**Kaufmann, L., Koppelstaetter, F., Delazer, M., Siedentopf, C., Rhomberg, P., Golaszewski, S., Felber, S., & Ischebeck, A. (2005, Apr 15). Neural correlates of distance and congruity effects in a numerical Stroop task: an event-related fMRI study. Neuroimage, 25(3), 888-898. https://doi.org/10.1016/j.neuroimage.2004.12.041

**Kerns, J. G., Cohen, J. D., MacDonald, A. W., 3rd, Johnson, M. K., Stenger, V. A., Aizenstein, H., & Carter, C. S. (2005, Oct). Decreased conflict- and error-related activity in the anterior cingulate cortex in subjects with schizophrenia. American Journal of Psychiatry, 162(10), 1833-1839. https://doi.org/10.1176/appi.ajp.162.10.1833

**Kim, C., Johnson, N. F., & Gold, B. T. (2014, Jan). Conflict adaptation in prefrontal cortex: now you see it, now you don't. Cortex, 50, 76-85. https://doi.org/10.1016/j.cortex.2013.08.011

**Kim, C., Kroger, J. K., & Kim, J. (2011, Feb). A functional dissociation of conflict processing within anterior cingulate cortex. Human Brain Mapping, 32(2), 304-312. https://doi.org/10.1002/hbm.21020

*Kohler, S., Bar, K. J., & Wagner, G. (2016, Jun). Differential involvement of brainstem noradrenergic and midbrain dopaminergic nuclei in cognitive control. Human Brain Mapping, 37(6), 2305-2318. https://doi.org/10.1002/hbm.23173

**Kohn, N., & Fernandez, G. (2020, Aug). Emotion and sex of facial stimuli modulate conditional automaticity in behavioral and neuronal interference in healthy men. Neuropsychologia, 145, 106592. https://doi.org/10.1016/j.neuropsychologia.2017.12.001

**Kozasa, E. H., Balardin, J. B., Sato, J. R., Chaim, K. T., Lacerda, S. S., Radvany, J., Mello, L., & Amaro, E., Jr. (2018). Effects of a 7-Day Meditation Retreat on the Brain Function of Meditators and Non-Meditators During an Attention Task. Frontiers in Human Neuroscience, 12, 222. https://doi.org/10.3389/fnhum.2018.00222

**Kronhaus, D. M., Lawrence, N. S., Williams, A. M., Frangou, S., Brammer, M. J., Williams, S. C., Andrew, C. M., & Phillips, M. L. (2006, Feb). Stroop performance in bipolar disorder: further evidence for abnormalities in the ventral prefrontal cortex. Bipolar Disord, 8(1), 28-39. https://doi.org/10.1111/j.1399-5618.2006.00282.x

**Kronke, K. M., Wolff, M., Mohr, H., Kraplin, A., Smolka, M. N., Buhringer, G., & Goschke, T. (2018, Aug). Monitor yourself! Deficient error-related brain activity predicts real-life self-control failures. Cognitive, Affective & Behavioral Neuroscience, 18(4), 622-637. https://doi.org/10.3758/s13415-018-0593-5

**Krug, M. K., & Carter, C. S. (2012, Oct 24). Proactive and reactive control during emotional interference and its relationship to trait anxiety. Brain Research, 1481, 13-36. https://doi.org/10.1016/j.brainres.2012.08.045

*Kuhn, S., Schubert, F., Mekle, R., Wenger, E., Ittermann, B., Lindenberger, U., & Gallinat, J. (2016, Jun). Neurotransmitter changes during interference task in anterior cingulate cortex: evidence from fMRI-guided functional MRS at 3 T. Brain Structure & Function, 221(5), 2541-2551. https://doi.org/10.1007/s00429-015-1057-0

**Lesh, T. A., Westphal, A. J., Niendam, T. A., Yoon, J. H., Minzenberg, M. J., Ragland, J. D., Solomon, M., & Carter, C. S. (2013). Proactive and reactive cognitive control and dorsolateral prefrontal cortex dysfunction in first episode schizophrenia. Neuroimage. Clinical, 2, 590-599. https://doi.org/10.1016/j.nicl.2013.04.010

*Li, M., Newton, A. T., Anderson, A. W., Ding, Z., & Gore, J. C. (2019, Mar 8). Characterization of the hemodynamic response function in white matter tracts for event-related fMRI. Nature Communications, 10(1), 1140. https://doi.org/10.1038/s41467-019-09076-2

**Loeffler, L. A. K., Satterthwaite, T. D., Habel, U., Schneider, F., Radke, S., & Derntl, B. (2019, Dec). Attention control and its emotion-specific association with cognitive emotion regulation in depression. Brain Imaging and Behavior, 13(6), 1766-1779. https://doi.org/10.1007/s11682-019-00174-9

**Manard, M., Francois, S., Phillips, C., Salmon, E., & Collette, F. (2017, Mar 1). The neural bases of proactive and reactive control processes in normal aging. Behavioural Brain Research, 320, 504-516. https://doi.org/10.1016/j.bbr.2016.10.026

**Mathis, A., Schunck, T., Erb, G., Namer, I. J., & Luthringer, R. (2009, Oct). The effect of aging on the inhibitory function in middle-aged subjects: a functional MRI study coupled with a color-matched Stroop task. International Journal of Geriatric Psychiatry, 24(10), 1062-1071. https://doi.org/10.1002/gps.2222

**Matthews, S. C., Paulus, M. P., Simmons, A. N., Nelesen, R. A., & Dimsdale, J. E. (2004, Jul). Functional subdivisions within anterior cingulate cortex and their relationship to autonomic nervous system function. Neuroimage, 22(3), 1151-1156. https://doi.org/10.1016/j.neuroimage.2004.03.005

**Mead, L. A., Mayer, A. R., Bobholz, J. A., Woodley, S. J., Cunningham, J. M., Hammeke, T. A., & Rao, S. M. (2002, Sep). Neural basis of the Stroop interference task: response competition or selective attention? Journal of the International Neuropsychological Society, 8(6), 735-742. https://doi.org/10.1017/s1355617702860015

*Milham, M. P., Banich, M. T., Webb, A., Barad, V., Cohen, N. J., Wszalek, T., & Kramer, A. F. (2001, Dec). The relative involvement of anterior cingulate and prefrontal cortex in attentional control depends on nature of conflict. Brain Research: Cognitive Brain Research, 12(3), 467-473. https://doi.org/10.1016/s0926-6410(01)00076-3

*Mitchell, R. L. (2005, Oct). The BOLD response during Stroop task-like inhibition paradigms: Effects of task difficulty and task-relevant modality. Brain and Cognition, 59(1), 23-37. https://doi.org/10.1016/j.bandc.2005.04.001

**Morgenroth, E., Orlov, N., Lythgoe, D. J., Stone, J. M., Barker, H., Munro, J., Eysenck, M., & Allen, P. (2019, Aug). Altered relationship between prefrontal glutamate and activation during cognitive control in people with high trait anxiety. Cortex, 117, 53-63. https://doi.org/10.1016/j.cortex.2019.02.021

*Nakao, T., Nakagawa, A., Yoshiura, T., Nakatani, E., Nabeyama, M., Yoshizato, C., Kudoh, A., Tada, K., Yoshioka, K., & Kawamoto, M. (2005, Jul 30). A functional MRI comparison of patients with obsessive-compulsive disorder and normal controls during a Chinese character Stroop task. Psychiatry Research, 139(2), 101-114. https://doi.org/10.1016/j.pscychresns.2004.12.004

*Ning, R. P. (2021, Nov). How language proficiency influences stroop effect and reverse-stroop effect: A functional magnetic resonance imaging study. Journal of Neurolinguistics, 60. https://doi.org/1016/j.jneuroling.2021.101027

*Norris, D. G., Zysset, S., Mildner, T., & Wiggins, C. J. (2002, Mar). An investigation of the value of spin-echo-based fMRI using a Stroop color-word matching task and EPI at 3 T. Neuroimage, 15(3), 719-726. https://doi.org/10.1006/nimg.2001.1005

**Ovaysikia, S., Tahir, K. A., Chan, J. L., & DeSouza, J. F. (2011). Word wins over face: emotional Stroop effect activates the frontal cortical network. Frontiers in Human Neuroscience, 4, 234. https://doi.org/10.3389/fnhum.2010.00234

**Overbeek, G., Gawne, T. J., Reid, M. A., Salibi, N., Kraguljac, N. V., White, D. M., & Lahti, A. C. (2019, Feb). Relationship Between Cortical Excitation and Inhibition and Task-Induced Activation and Deactivation: A Combined Magnetic Resonance Spectroscopy and Functional Magnetic Resonance Imaging Study at 7T in First-Episode Psychosis. Biological Psychiatry: Cognitive Neuroscience and Neuroimaging, 4(2), 121-130. https://doi.org/10.1016/j.bpsc.2018.10.002

**Papalini, S., Michels, F., Kohn, N., Wegman, J., van Hemert, S., Roelofs, K., Arias-Vasquez, A., & Aarts, E. (2019, Feb). Stress matters: Randomized controlled trial on the effect of probiotics on neurocognition. Neurobiology of Stress, 10, 100141. https://doi.org/10.1016/j.ynstr.2018.100141

*Pardo, J. V., Pardo, P. J., Janer, K. W., & Raichle, M. E. (1990, Jan). The anterior cingulate cortex mediates processing selection in the Stroop attentional conflict paradigm. Proceedings of the National Academy of Sciences of the United States of America, 87(1), 256-259. https://doi.org/10.1073/pnas.87.1.256

**Park, I. H., Park, H. J., Chun, J. W., Kim, E. Y., & Kim, J. J. (2008, Aug 1). Dysfunctional modulation of emotional interference in the medial prefrontal cortex in patients with schizophrenia. Neuroscience Letters, 440(2), 119-124. https://doi.org/10.1016/j.neulet.2008.05.094

**Peven, J. C., Litz, G. A., Brown, B., Xie, X., Grove, G. A., Watt, J. C., & Erickson, K. I. (2019, Dec 26). Higher Cardiorespiratory Fitness is Associated with Reduced Functional Brain Connectivity During Performance of the Stroop Task. Brain plasticity, 5(1), 57-67. https://doi.org/10.3233/BPL-190085

**Piai, V., Roelofs, A., Acheson, D. J., & Takashima, A. (2013). Attention for speaking: domain-general control from the anterior cingulate cortex in spoken word production. Frontiers in Human Neuroscience, 7, 832. https://doi.org/10.3389/fnhum.2013.00832

*Pinel, P., Piazza, M., Le Bihan, D., & Dehaene, S. (2004, Mar 25). Distributed and overlapping cerebral representations of number, size, and luminance during comparative judgments. Neuron, 41(6), 983-993. https://doi.org/10.1016/s0896-6273(04)00107-2

*Polk, T. A., Drake, R. M., Jonides, J. J., Smith, M. R., & Smith, E. E. (2008, Dec 17). Attention enhances the neural processing of relevant features and suppresses the processing of irrelevant features in humans: a functional magnetic resonance imaging study of the Stroop task. Journal of Neuroscience, 28(51), 13786-13792. https://doi.org/10.1523/JNEUROSCI.1026-08.2008

**Pompei, F., Jogia, J., Tatarelli, R., Girardi, P., Rubia, K., Kumari, V., & Frangou, S. (2011, Jun 1). Familial and disease specific abnormalities in the neural correlates of the Stroop Task in Bipolar Disorder. Neuroimage, 56(3), 1677-1684. https://doi.org/10.1016/j.neuroimage.2011.02.052

**Portes, B., Balardin, J. B., Lacerda, S., Pires, F., Tobo, P., Barrichello, C., Peterson, J., Sanches, L. R., Sanches-Rocha, L., Amaro, E., Jr., & Kozasa, E. H. (2019, Jun). The effects of perceived chronic stress on the fMRI correlates of attentional control in women managers. Archives of Women's Mental Health, 22(3), 375-381. https://doi.org/10.1007/s00737-018-0902-6

*Potenza, M. N., Leung, H. C., Blumberg, H. P., Peterson, B. S., Fulbright, R. K., Lacadie, C. M., Skudlarski, P., & Gore, J. C. (2003, Nov). An FMRI Stroop task study of ventromedial prefrontal cortical function in pathological gamblers. American Journal of Psychiatry, 160(11), 1990-1994. https://doi.org/10.1176/appi.ajp.160.11.1990

**Prakash, R. S., Erickson, K. I., Colcombe, S. J., Kim, J. S., Voss, M. W., & Kramer, A. F. (2009, Dec). Age-related differences in the involvement of the prefrontal cortex in attentional control. Brain and Cognition, 71(3), 328-335. https://doi.org/10.1016/j.bandc.2009.07.005

** Puente, A. N., Faraco, C., Terry, D. P., Brown, C., & Miller, L. S. (2014). Minimal functional brain differences between older adults with and without mild cognitive impairment during the stroop. Neuropsychology, Development, and Cognition. Section B: Aging, Neuropsychology and Cognition, 21(3), 346-369. https://doi.org/10.1080/13825585.2013.824065

*Purmann, S., & Pollmann, S. (2015). Adaptation to recent conflict in the classical color-word Stroop-task mainly involves facilitation of processing of task-relevant information. Frontiers in Human Neuroscience, 9, 88. https://doi.org/10.3389/fnhum.2015.00088

**Ramm, M., Sundermann, B., Gomes, C. A., Moddel, G., Langenbruch, L., Nayyeri, M. D., Young, P., Pfleiderer, B., Krebs, R. M., & Axmacher, N. (2021, Feb 1). Probing the relevance of the hippocampus for conflict-induced memory improvement. Neuroimage, 226, 117563. https://doi.org/10.1016/j.neuroimage.2020.117563

*Ravnkilde, B., Videbech, P., Rosenberg, R., Gjedde, A., & Gade, A. (2002, Jun). Putative tests of frontal lobe function: a PET-study of brain activation during Stroop's Test and verbal fluency. Journal of Clinical and Experimental Neuropsychology, 24(4), 534-547. https://doi.org/10.1076/jcen.24.4.534.1033

*Roberts, K. L., & Hall, D. A. (2008, Jun). Examining a supramodal network for conflict processing: a systematic review and novel functional magnetic resonance imaging data for related visual and auditory stroop tasks. Journal of Cognitive Neuroscience, 20(6), 1063-1078. https://doi.org/10.1162/jocn.2008.20074

**Robertson, B. D., Hiebert, N. M., Seergobin, K. N., Owen, A. M., & MacDonald, P. A. (2015, Jul 1). Dorsal striatum mediates cognitive control, not cognitive effort per se, in decision-making: An event-related fMRI study. Neuroimage, 114, 170-184. https://doi.org/10.1016/j.neuroimage.2015.03.082

**Roth, R. M., Koven, N. S., Randolph, J. J., Flashman, L. A., Pixley, H. S., Ricketts, S. M., Wishart, H. A., & Saykin, A. J. (2006, Jul 31). Functional magnetic resonance imaging of executive control in bipolar disorder. Neuroreport, 17(11), 1085-1089. https://doi.org/10.1097/01.wnr.0000227979.06013.57

**Ruff, C. C., Woodward, T. S., Laurens, K. R., & Liddle, P. F. (2001, Nov). The role of the anterior cingulate cortex in conflict processing: evidence from reverse stroop interference. Neuroimage, 14(5), 1150-1158. https://doi.org/10.1006/nimg.2001.0893

*Salgado-Pineda, P., Rodriguez-Jimenez, R., Moreno-Ortega, M., Dompablo, M., Martinez de Aragon, A., Salvador, R., McKenna, P. J., Pomarol-Clotet, E., & Palomo, T. (2021, Dec). Activation and deactivation patterns in schizophrenia during performance of an fMRI adapted version of the stroop task. Journal of Psychiatric Research, 144, 1-7. https://doi.org/10.1016/j.jpsychires.2021.09.039

* Salgado-Pineda, P., Vendrell, P., Bargallo, N., Falco n, C., & Junque, C. (2002, Apr 1-15). [Functional magnetic resonance in the evaluation of the activity of the anterior cingulate cortex using Stroop's paradigm]. Revista de Neurología, 34(7), 607-611. https://www.ncbi.nlm.nih.gov/pubmed/12080508 (Resonancia magnetica funcional en la evaluacion de la actividad del cingulado anterior mediante el paradigma de Stroop.)

**Schmidt, C., Peigneux, P., Leclercq, Y., Sterpenich, V., Vandewalle, G., Phillips, C., Berthomier, P., Berthomier, C., Tinguely, G., Gais, S., Schabus, M., Desseilles, M., Dang-Vu, T., Salmon, E., Degueldre, C., Balteau, E., Luxen, A., Cajochen, C., Maquet, P., & Collette, F. (2012). Circadian preference modulates the neural substrate of conflict processing across the day. PloS One, 7(1), e29658. https://doi.org/10.1371/journal.pone.0029658

**Schulte, T., Muller-Oehring, E. M., Sullivan, E. V., & Pfefferbaum, A. (2012, Feb 1). Synchrony of corticostriatal-midbrain activation enables normal inhibitory control and conflict processing in recovering alcoholic men. Biological Psychiatry, 71(3), 269-278. https://doi.org/10.1016/j.biopsych.2011.10.022

**Schulte, T., Muller-Oehring, E. M., Vinco, S., Hoeft, F., Pfefferbaum, A., & Sullivan, E. V. (2009, Nov 1). Double dissociation between action-driven and perception-driven conflict resolution invoking anterior versus posterior brain systems. Neuroimage, 48(2), 381-390. https://doi.org/10.1016/j.neuroimage.2009.06.058

*Seok Jeong, B., Kwon, J. S., Yoon Kim, S., Lee, C., Youn, T., Moon, C. H., & Yoon Kim, C. (2005, Aug 30). Functional imaging evidence of the relationship between recurrent psychotic episodes and neurodegenerative course in schizophrenia. Psychiatry Research, 139(3), 219-228. https://doi.org/10.1016/j.pscychresns.2004.01.008

**Shashidhara, S., Spronkers, F. S., & Erez, Y. (2020, Jul). Individual-subject Functional Localization Increases Univariate Activation but Not Multivariate Pattern Discriminability in the "Multiple-demand" Frontoparietal Network. Journal of Cognitive Neuroscience, 32(7), 1348-1368. https://doi.org/10.1162/jocn_a_01554

*Sheu, L. K., Jennings, J. R., & Gianaros, P. J. (2012, Jul). Test-retest reliability of an fMRI paradigm for studies of cardiovascular reactivity. Psychophysiology, 49(7), 873-884. https://doi.org/10.1111/j.1469-8986.2012.01382.x

**Shin, G., & Kim, C. (2015, Jun). Neural correlates of cognitive style and flexible cognitive control. Neuroimage, 113, 78-85. https://doi.org/10.1016/j.neuroimage.2015.03.046

*Silton, R. L., Heller, W., Towers, D. N., Engels, A. S., Spielberg, J. M., Edgar, J. C., Sass, S. M., Stewart, J. L., Sutton, B. P., Banich, M. T., & Miller, G. A. (2010, Apr 15). The time course of activity in dorsolateral prefrontal cortex and anterior cingulate cortex during top-down attentional control. Neuroimage, 50(3), 1292-1302. https://doi.org/10.1016/j.neuroimage.2009.12.061

**Song, Y., & Hakoda, Y. (2015, Sep 1). An fMRI study of the functional mechanisms of Stroop/reverse-Stroop effects. Behavioural Brain Research, 290, 187-196. https://doi.org/10.1016/j.bbr.2015.04.047

*Steel, C., Haworth, E. J., Peters, E., Hemsley, D. R., Sharma, T., Gray, J. A., Pickering, A., Gregory, L., Simmons, A., Bullmore, E. T., & Williams, S. C. (2001, Nov 16). Neuroimaging correlates of negative priming. Neuroreport, 12(16), 3619-3624. https://doi.org/10.1097/00001756-200111160-00049

**Taylor, S. F., Kornblum, S., Lauber, E. J., Minoshima, S., & Koeppe, R. A. (1997, Aug). Isolation of specific interference processing in the Stroop task: PET activation studies. Neuroimage, 6(2), 81-92. https://doi.org/10.1006/nimg.1997.0285

**Terry, D. P., Faraco, C. C., Smith, D., Diddams, M. J., Puente, A. N., & Miller, L. S. (2012). Lack of long-term fMRI differences after multiple sports-related concussions. Brain Injury, 26(13-14), 1684-1696. https://doi.org/10.3109/02699052.2012.722259

**van 't Ent, D., den Braber, A., Rotgans, E., de Geus, E. J., & de Munck, J. C. (2014, May 30). The use of fMRI to detect neural responses to cognitive interference and planning: evidence for a contribution of task related changes in heart rate? Journal of Neuroscience Methods, 229, 97-107. https://doi.org/10.1016/j.jneumeth.2014.04.013

*van de Meerendonk, N., Rueschemeyer, S. A., & Kolk, H. H. (2013, Sep). Language comprehension interrupted: both language errors and word degradation activate Broca's area. Brain and Language, 126(3), 291-301. https://doi.org/10.1016/j.bandl.2013.07.004

*Verdolini, N., Moreno-Ortega, M., Salgado-Pineda, P., Monte, G., de Aragon, A. M., Dompablo, M., McKenna, P. J., Salvador, R., Palomo, T., Pomarol-Clotet, E., & Rodriguez-Jimenez, R. (2023, May 15). Failure of deactivation in bipolar disorder during performance of an fMRI adapted version of the Stroop task. Journal of Affective Disorders, 329, 307-314. https://doi.org/10.1016/j.jad.2023.02.132

**Veroude, K., Jolles, J., Croiset, G., & Krabbendam, L. (2013, Jul). Changes in neural mechanisms of cognitive control during the transition from late adolescence to young adulthood. Developmental Cognitive Neuroscience, 5, 63-70. https://doi.org/10.1016/j.dcn.2012.12.002

**Verstynen, T. D. (2014, Nov 15). The organization and dynamics of corticostriatal pathways link the medial orbitofrontal cortex to future behavioral responses. Journal of Neurophysiology, 112(10), 2457-2469. https://doi.org/10.1152/jn.00221.2014

*Wagner, G., De la Cruz, F., Schachtzabel, C., Gullmar, D., Schultz, C. C., Schlosser, R. G., Bar, K. J., & Koch, K. (2015, May). Structural and functional dysconnectivity of the fronto-thalamic system in schizophrenia: a DCM-DTI study. Cortex, 66, 35-45. https://doi.org/10.1016/j.cortex.2015.02.004

**Wallentin, M., Gravholt, C. H., & Skakkebaek, A. (2015, Dec). Broca's region and Visual Word Form Area activation differ during a predictive Stroop task. Cortex, 73, 257-270. https://doi.org/10.1016/j.cortex.2015.08.023

** Wang, J. X., Li, Y., Mu, Y., & Zhuang, J. Y. (2023, Mar 21). Common and unique neural mechanisms of social and nonsocial conflict resolving and adaptation. Cerebral Cortex, 33(7), 3773-3786. https://doi.org/10.1093/cercor/bhac306

**Ye, Z., & Zhou, X. (2009, Oct 15). Conflict control during sentence comprehension: fMRI evidence. Neuroimage, 48(1), 280-290. https://doi.org/10.1016/j.neuroimage.2009.06.032

*Zhu, Z., Feng, G., Zhang, J. X., Li, G., Li, H., & Wang, S. (2013, Aug 1). The role of the left prefrontal cortex in sentence-level semantic integration. Neuroimage, 76, 325-331. https://doi.org/10.1016/j.neuroimage.2013.02.060

**Zoccatelli, G., Beltramello, A., Alessandrini, F., Pizzini, F. B., & Tassinari, G. (2010, Nov). Word and position interference in stroop tasks: a behavioral and fMRI study. Experimental Brain Research, 207(1-2), 139-147. https://doi.org/10.1007/s00221-010-2433-x

**Zysset, S., Muller, K., Lohmann, G., & von Cramon, D. Y. (2001, Jan). Color-word matching stroop task: separating interference and response conflict. Neuroimage, 13(1), 29-36. https://doi.org/10.1006/nimg.2000.0665

**Zysset, S., Schroeter, M. L., Neumann, J., & von Cramon, D. Y. (2007, Jun). Stroop interference, hemodynamic response and aging: an event-related fMRI study. Neurobiology of Aging, 28(6), 937-946. https://doi.org/10.1016/j.neurobiolaging.2006.05.008
